# Colistin Dependence in Extensively Drug-Resistant *Acinetobacter baumannii* Strain Is Associated with IS*Ajo2* and IS*Aba13* Insertions and Multiple Cellular Responses

**DOI:** 10.3390/ijms22020576

**Published:** 2021-01-08

**Authors:** Sherley Chamoun, Jenny Welander, Mihaela-Maria Martis-Thiele, Maria Ntzouni, Carina Claesson, Elena Vikström, Maria V. Turkina

**Affiliations:** 1 Department of Biomedical and Clinical Sciences, Faculty of Medicine and Health Sciences, Linköping University, SE-58185 Linköping, Sweden; sherleychamoun@gmail.com (S.C.); martis.mihaela@gmail.com (M.-M.M.-T.); elena.vikstrom@liu.se (E.V.); 2 Department of Biomedical and Clinical Sciences, Linköping University and Department of Clinical Microbiology, University Hospital, SE-58185 Linköping, Sweden; Jenny.Welander@regionostergotland.se (J.W.); Carina.Claesson@regionostergotland.se (C.C.); 3 National Bioinformatics Infrastructure Sweden, Bioinformatics Platform, Science for Life Laboratory, SE-17165 Solna, Sweden; 4 Core Facility, Faculty of Medicine and Health Sciences, Linköping University, SE-58185 Linköping, Sweden; maria.ntzouni@liu.se

**Keywords:** *Acinetobacter baumannii*, colistin dependence, multidrug resistance, polymyxin, insertion sequence elements, proteomics, WGS, T6SS

## Abstract

The nosocomial opportunistic Gram-negative bacterial pathogen *Acinetobacter baumannii* is resistant to multiple antimicrobial agents and an emerging global health problem. The polymyxin antibiotic colistin, targeting the negatively charged lipid A component of the lipopolysaccharide on the bacterial cell surface, is often considered as the last-resort treatment, but resistance to colistin is unfortunately increasing worldwide. Notably, colistin-susceptible *A. baumannii* can also develop a colistin dependence after exposure to this drug in vitro. Colistin dependence might represent a stepping stone to resistance also in vivo. However, the mechanisms are far from clear. To address this issue, we combined proteogenomics, high-resolution microscopy, and lipid profiling to characterize and compare *A. baumannii* colistin-susceptible clinical isolate (Ab-S) of to its colistin-dependent subpopulation (Ab-D) obtained after subsequent passages in moderate colistin concentrations. Incidentally, in the colistin-dependent subpopulation the *lpxA* gene was disrupted by insertion of IS*Ajo2*, the lipid A biosynthesis terminated, and Ab-D cells displayed a lipooligosaccharide (LOS)-deficient phenotype. Moreover, both *mlaD* and *pldA* genes were perturbed by insertions of IS*Ajo2* and IS*Aba13*, and LOS-deficient bacteria displayed a capsule with decreased thickness as well as other surface imperfections. The major changes in relative protein abundance levels were detected in type 6 secretion system (T6SS) components, the resistance-nodulation-division (RND)-type efflux pumps, and in proteins involved in maintenance of outer membrane asymmetry. These findings suggest that colistin dependence in *A. baumannii* involves an ensemble of mechanisms seen in resistance development and accompanied by complex cellular events related to insertional sequences (ISs)-triggered LOS-deficiency. To our knowledge, this is the first study demonstrating the involvement of IS*Ajo2* and IS*Aba13* IS elements in the modulation of the lipid A biosynthesis and associated development of dependence on colistin.

## 1. Introduction

*Acinetobacter baumannii* is a nosocomial opportunistic Gram-negative pathogen displaying extensive resistance to many classes of antimicrobial agents. An arsenal of virulence factors, such as biofilm production, capsular polysaccharides, drug efflux pumps, release of outer membrane vesicles, and altered outer membrane protein composition, help *A. baumannii* to adapt to environmental stresses and promote an infection [1]. To cope with antibiotic exposure, it employs both intrinsic and acquired resistance mechanisms, including membrane impermeability, enzymatic modification of the drug, target alteration, and active drug efflux via several efflux systems. Carbapenem-resistant *A. baumannii* (CRAB) is the top-ranked pathogen in the priority list of the World Health Organization for research and development of new antibiotic treatments [2]. Carbapenem resistance in *A. baumannii* often involves co-resistance to other antibiotic classes [2,3], thereby causing life-threatening nosocomial infections. Colistin, or polymyxin E, is employed to treat CRAB infections, and it is considered as the last-resort drug against infections caused by multi-resistant bacteria. Colistin interacts with the lipid A component of the lipopolysaccharide (LPS) or lipooligosaccharide (LOS) in the outer membrane (OM) of the cell envelope, destabilizing and disrupting both their OM and inner membrane (IM) and killing bacteria [4].

The OM of Gram-negative bacteria is asymmetric with the inner leaflet comprised of phospholipids and the outer leaflet of LPS or LOS. LPS is composed of a lipid A anchor, a core oligosaccharide, and an O-polysaccharide, while *A. baumannii* LOS does not contain O-polysaccharide and instead have a short series of sugars attached to the core. LPS/LOS are synthetized on the cytoplasmic side of inner membrane and transported across the IM and periplasm before they reach the OM. The negatively charged hydrophobic glycolipid lipid A anchors the LPS/LOS to the OM. The LPS/LOS are essential for Gram-negative bacteria, providing a barrier structure and stringent permeability to benefit the survival of the pathogen under stress conditions [5]. Nevertheless, some strains of *A. baumannii* can survive without lipid A and hence without LOS [6,7].

Intrinsic colistin resistance emerges primarily through remodeling the drug target, the lipid A moiety, as a result of mutations, deletions, or insertions of mobile genetic elements in various genes involved in lipid A biosynthesis. Two major mechanisms of chromosomally encoded colistin resistance have been identified. The first includes covalent modifications of the lipid A phosphoester groups that reduce the net negative charge of lipid A and alters the affinity for polymyxins. It’s affects the PmrAB two-component signaling system and includes the addition of phosphoethanolamine (pETN) [8,9] or 4-amino-4-deoxy-l-arabinose in *Enterobacteriaceae* [4] to the lipid A. The second is a complete loss of LOS due to mutations in one of three first genes involved in the lipid A biosynthesis (lpxA, lpxC, and lpxD) [10,11], yielding a termination of lipid A production.

Colistin-susceptible *A. baumannii* strains may develop dependence after exposure to colistin in vitro [12,13,14]. Colistin dependence is not rare, as many as 32.9% of colistin-susceptible isolates were able to do so [14], most of them were otherwise multidrug-resistant and able to cause infection in mice [15]. Colistin dependence may represent a transition step to resistance and LOS-deficiency, with direct binding of colistin to phosphatidylglycerol-rich areas of the OM in LOS-deficient strains, has been proposed as a key step [15]. Still, the mechanisms underlying colistin dependence are far from clear.

To address this issue, we employed a strain-specific proteogenomic approach integrating high-throughput discovery-based genomics and proteomics. The interpretation of mass spectrometry (MS)-based proteomics data typically include peptides identification by matching the acquired MS data against a standard reference protein sequence database, such as NCBI or UniProt, assuming that all peptides are present therein [16]. This is, however, not always the case, particularly for bacteria due to significant intraspecies genomic variability and presence of mobile elements [17]. We, therefore, utilized whole genome sequencing (WGS) of susceptible *A. baumannii* (Ab-S) and a colistin-dependent subpopulation (Ab-D), not only to characterize possible differences in genomic features, but also to create in silico a comprehensive set of possible protein-coding sequences for each bacterial subpopulation. The tailored databases were then used for proteomic analyses and downstream bioinformatics. Moreover, we combined the proteogenomic data with high-resolution imaging and lipid profiling to provide a careful characterization of colistin dependence.

## 2. Results

### 2.1. Phenotypically Colistin-Dependent Subpopulation

**Figure 1:**
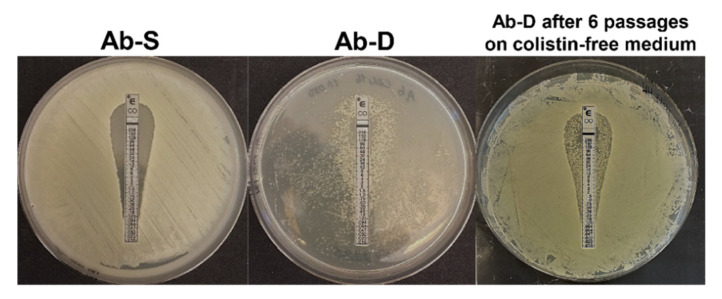
Diversity of the growth pattern of *A. baumannii* clinical isolate. Initially, this isolate was characterized as susceptible to colistin by Etest on MHA, S ≤ 2 µg/mL (Ab-S), on the left. A bacterial subpopulation displaying colistin-dependent growth was selected after 8-10 passages on agar containing 16 µg/mL colistin sulphate. This colistin-dependent population (Ab-D) grows only along the colistin Etest strip on a pear-like shape area between 0.125 and 256 µg/mL, (**middle panel**). When the Ab-D subpopulation was passed 6 times on agar base medium and further applied for colistin Etest, a phenotypic heterogeneity within this bacterial population was observed (Ab-D after 6 passages on colistin-free medium). Most cells were growing in a colistin-susceptible manner and a certain amount retained a colistin-dependent growth pattern appearing along the colistin Etest strip (**right panel**).

A colistin-dependent phenotypic subpopulation (Ab-D) evolved in vitro from the colistin-susceptible *A. baumannii* clinical isolate after only 8–10 subsequent passages in the presence of a moderate colistin concentration. The Ab-D subpopulation appeared as numerous colonies growing generally along the colistin Etest strip with a pear-like shape area between 0.125 and 256 µg/mL, whereas the original isolate Ab-S was susceptible to colistin with minimum inhibitory concentration (MIC) ≤ 2 µg/mL (Figure 1).

When the MICs of 17 antimicrobial agents, including colistin, were evaluated using the broth microdilution method, the colistin MIC for the dependent Ab-D phenotype exceeded 8 μg/mL in comparison to MIC ≤ 2 µg/mL for Ab-S (Table S1). The latter did, however, displayed a high degree of drug resistance, for instance, to carbapenems, and was accordingly classified as extensively drug-resistant (XDR) [18]. Interestingly, the Ab-D subpopulation displayed lower MIC-values for meropenem, imipenem, amikacin, tigecycline, piperacillin-tazobactam, and amoxicillin-clavulanic acid compared to Ab-S (Table S1). The stability of the dependent phenotype was limited, but the characteristics were maintained during a few passages in colistin-free medium. They were, however, only partially reversible, as subsequent growth on antibiotic-free medium after six subculture passages yielded phenotypic heterogeneity within this bacterial population. Most cells displayed a colistin-susceptible manner but some retained a colistin-dependent growth pattern appearing along the colistin Etest strip (Figure 1).

### 2.2. Ultrastructural Traits of Colistin-Dependent A. baumannii

**Figure 2:**
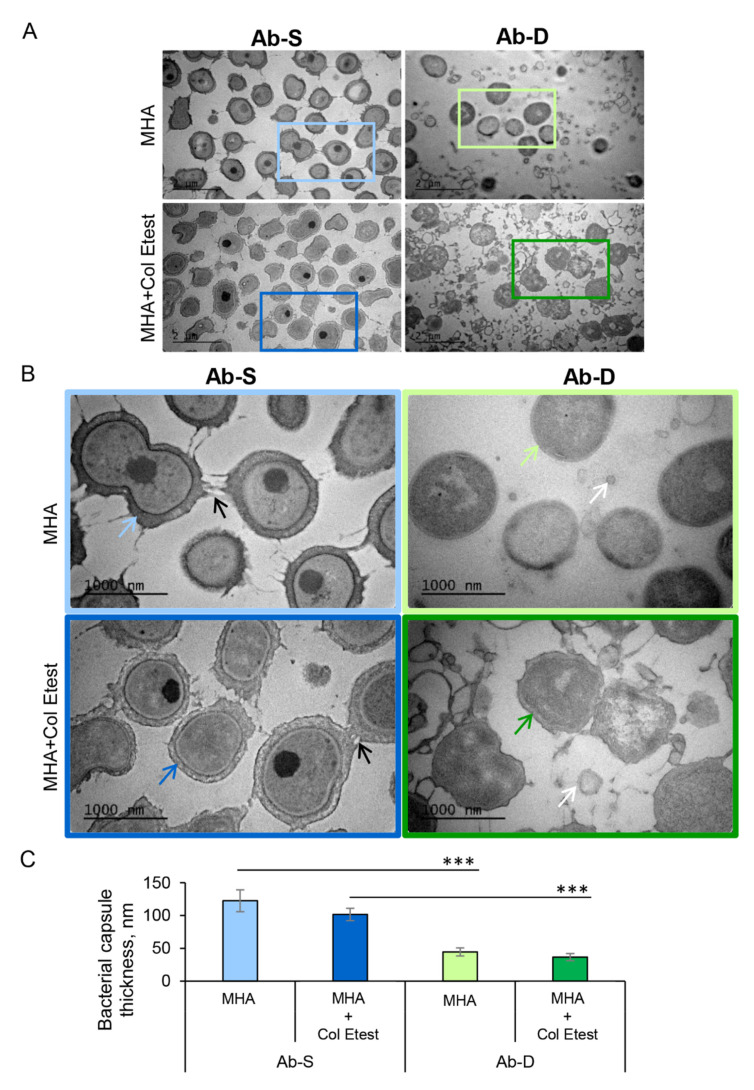
High-resolution visualization of *A. baumannii* populations and their capsular structure. Two *A. baumannii* populations, susceptible to colistin (Ab-S) and colistin-dependent (Ab-D) were grown on MHA plates (MHA) or MHA plates with colistin Etest strip (MHA + colistin Etest). Cells were collected, fixed, stained with uranyl acetate, and analyzed by TEM. The images are from one representative of three independent experiments. (**A**) Main panels, bars: 2 µm. (**B**) Inserts, bars: 1000 µm. Black arrows point on appendage-like structures such as pili and fimbriae. White arrows specify outer membrane vesicles. Colored blue and green arrows point on exopolysaccharide capsule layers. (**C**) Quantification of the bacterial capsule thickness, nm. Columns represent the mean ± SE. Significant differences are indicated with *** when *p* > 0.001, as analyzed by two-tailed Student’s *t*-test. Data from three different experiments, and 100 different capsule areas in at least 50 different cells per condition were analyzed (same color code as in insert images).

Development of resistance or dependence to colistin may occur via alterations in bacterial structures that are located outside or within the cell envelope, thereby affecting the bacterial fitness. To address this issue, we employed super resolution imaging by transmission electron microscopy (TEM) of the two *A. baumannii* subpopulations, Ab-S and Ab-D (Figure 2A,B). We observed that, under both conditions, Ab-S cells displayed a regular shape of the cell envelope with appendage-like structures, such as pili or fimbriae and a thick exopolysaccharide capsule layer. By contrast, Ab-D bacteria showed a cell envelope with increased membrane curvature, few appendages, and a thin capsule, especially when grown on MHA plates in close proximity of the colistin Etest strip. The Ab-D cells were furthermore surrounded by additional electron-dense material, like cell debris and/or outer membrane vesicles (Figure 2B). Ab-S bacteria showed a regular and thick capsule, reaching approximately 100 nm, while Ab-D cells displayed a disrupted capsule, as evidenced by an at least 2-fold decrease in thickness (Figure 2C). Thus, these high-resolution imaging demonstrates that Ab-D cells carry multiple surface imperfections.

### 2.3. Variations in the Lipid A Content

**Figure 3:**
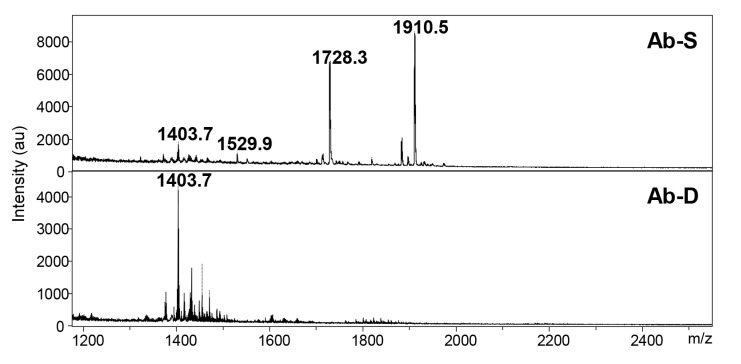
The mass spectra for lipid A in *A. baumannii* populations. Two *A. baumannii* populations, susceptible to colistin (Ab-S) and colistin-dependent (Ab-D) were grown on MHA plates with colistin Etest strip. Cells were collected, lipid A was extracted, and samples were subjected to MALDI-TOF MS analysis. In Ab-S, the mass spectrum of lipid A was dominated by peaks at *m*/*z* values of 1910.5, 1728.3 and 1529.9 (**upper panel**). The major peaks 1910.5 and 1728.3 were missing in the Ab-D mass spectrum (**lower panel**).

A possible mechanism for colistin dependence is attributed to defects in or modifications of the lipid A moiety of the LPS, which may result in an impaired cell membrane [13]. We utilized Matrix-Assisted Laser Desorption Ionization-Time of Flight (MALDI-TOF) MS to investigate the lipid A structure in Ab-S and Ab-D. This method allows a rapid and accurate detection of lipid A possible modifications, including a potential pETN addition [19,20]. In Ab-S, the mass spectrum of lipid A was attributed by peaks at *m*/*z* values of 1910.5, 1728.3, and 1529.9 (Figure 3). The two most intensive signals at *m*/*z* 1910.5 and *m*/*z* 1728.3 were produced by singly deprotonated bis-phosphorylated hepta-acyl and bis-phosphorylated hexa-acyl lipid A, which is consistent with previous studies [21,22]. The major peaks 1910.5, 1728.3, 1529.9, were missing in the mass spectrum for lipid A from the Ab-D cells (Figure 3). This likely reflects a lipid A deficiency in the Ab-D subpopulation. It is worth noting that no peaks were detected corresponding to previously described pETN-modified mono- and bis-phosphorylated hepta-acyl lipid A [19,20,21,23,24], indicating that colistin dependence in Ab-D is not associated with the addition of pETN. Taken together, MALDI-TOF MS analyses demonstrated structural deficiencies in lipid A, that most likely lead to structural defects also in the LOS and outer membrane.

### 2.4. Whole Genome Sequencing Reveals Genes Associated with Antibiotic Resistance

To investigate potential genetic differences, we performed whole genome sequencing (WGS) of Ab-S and Ab-D with downstream bioinformatics analyses. The whole genome size of *A. baumannii* was 4,111,821 and 4,125,623 base pairs (bp) for the Ab-S and Ab-D populations (Table 1, Supplementary files S1 and S2), whose genome data could be assembled into 121 and 186 contigs for Ab-S and Ab-D, respectively. As many as 109 contigs (59%) in Ab-D included less than 1000 bp, which is significantly more than the 35% in Ab-S. On the other hand, the number of sequences longer than 1000 bp was nearly the same for Ab-S and Ab-D (79 and 77 respectively). When the complete genome sequences were searched against the NCBI bacteria database, the reference genome with NCBI accession NC_011586 corresponding to *A. baumannii* MDR strain AB0057 was identified as closest in sequence with 95.1% and 90.9% sequence match for Ab-S and Ab-D, respectively. This indicates that the strain used here might have evolved from the AB0057, first isolated from a blood-stream infection patient at Walter Reed Army Medical Center [25,26,27]. Upon multi-locus sequence typing (MLST), Ab-S and Ab-D had sequence type 1 (ST1).

The assembled data were also searched for antimicrobial resistance genes using the ResFinder database [28]. This analysis of the resistome revealed presence of five groups of genes associated with antibiotic resistance identical in both Ab-S and Ab-D subpopulations: aminoglycoside-modifying enzymes (*aac(3)-IId*, *aph(3′)-VIa*, *aph(3′)-Ic*, *aac(3)-Ia*, *aadA1*); beta-lactamases (*bla_OXA-69_*, *bla_TEM-1D_*, *bla_OXA-23_*, *bla_ADC-25_*, *bla_NDM-1_*); phenicols (*floR*, *catA1*); sulphonamide (*sul1*) and tetracycline (*tetA)* (Table 2). This list of the identified resistance genes was rather similar to that for AB0057 [25,29]. No mobile plasmid-mediated resistance *mcr* genes were found even after manual verification and no differences between Ab-S and Ab-D in polymyxin resistance-associated genes *pmrA*, *pmrB* and *pmrC* were identified.

We further analyzed the plasmid content of susceptible and dependent *A. baumannii* using PLSDB, a resource of complete bacterial plasmids retrieved from the NCBI database [30]. PLSDB analysis identified 4 plasmids and no difference between Ab-S and Ab-D was observed (Table S2). SNPs analysis was performed, but did not find any differences between the genomes. However, as the variant call is based on read mapping, the SNP analysis cannot reveal differences in insertion sequences (ISs) elements, as these can be present in multiple copies and map to multiple different positions.

### 2.5. In Silico Genome Analysis Displays Multiple ISs

ISs mediate genome rearrangement, disturb coding sequences by insertional inactivation, and modulate gene expression enabling bacterial adaptability and resistance to antibacterial agents [31]. Some ISs are known to be involved in both colistin resistance [32,33] and dependence development [34]. Therefore, we further investigated the ISs content in genome sequences of Ab-S and Ab-D. Multiple ISs elements, both truncated and full-length, were identified using ISfinder [35]. ISfinder recognized 411 copies of IS-related events present in 73 contigs of the Ab-S dataset among all sequences and 652 IS-related events in 140 contigs of the Ab-D dataset (Table S2). Both subpopulations shared similar ISs composition from 13 families (IS*5*, IS*6*, IS*4*, IS*3*, ISNCY (IS Not Classified Yet), Tn3, IS*30*, IS*L3*, IS*1*, IS*66*, IS*256*, IS*1182* and IS*481*) with ISs elements located predominantly in the beginning and at the very end of contigs (Table 3). IS*Aba1*, IS*Ajo2*, and IS*Aba13* had more than 10 ISs copies per genome detected in both Ab-S and Ab-D (Table 4), but the number of full-length ISs was low. WGS identified two copies of IS*Ajo2* full-length gene and three copies of IS*Aba13* full-length gene. IS*Aba1* and IS*Aba123* were encoded by single full-length gene copy in either of them. WGS identified two copies of IS*Ajo2* full-length gene and three copies of IS*Aba13* full-length gene in both Ab-S and Ab-D. IS*Aba1* and IS*Aba123* were encoded by single full-length gene copy each in both Ab-S and Ab-D. No additional novel ISs were detected in Ab-D in comparison to Ab-S. In Ab-S, the IS*5* family showed the largest number of ISs and the most frequently detected IS element was IS*Aba1* (*n* = 26) belonging to the IS*4* family, and with a similar number for IS*Aba33* (Table S3). It should be noted, however, that IS*Aba33* shares 87% identity with IS*Aba1*, which is typical for ISs belonging to the same family; thus, many identified ISs are difficult to distinguish due to sequences overlap. The most substantial difference between Ab-S and Ab-D was a dramatic increase in the number of IS*Ajo2* from 18 in Ab-S to 84 in Ab-D (Table 4). *ISAjo2* was initially described for the *Acinetobacter johnsonii* genome and belongs to the ISNCY family [36]. Interestingly, less than 1000 bp short IS*Ajo2*-borne contigs were overrepresented (77%) in Ab-D compared to Ab-S (22%). This difference can likely be attributed to the high levels of DNA repetitiveness, i.e., the presence of multiple identical or highly homologous ISs flanked by short terminal inverted repeats creating ambiguities in alignment and assembly, resulting in the presence of multiple short contigs [37].

### 2.6. Proteome Alterations

Genomics alone is not enough for understanding the biology and phenotypic capabilities of the organisms, such as responses to environmental perturbations. Thus, to provide a more complete characterization we utilized a comprehensive strain-specific combination of genomics and proteomics. First, to generate a complete set of possible protein-coding sequences, the acquired WGSs of Ab-D and Ab-S were translated in silico in six reading frames. Based on detected open reading frames (ORFs), phenotype-specific Ab-D and Ab-S databases, containing 60,560 and 60,210 potential protein sequences, respectively, were built. (Supplementary files S3 and S4). Second, we used the resulting protein databases in FASTA-format for analyses of high-resolution mass spectrometry data. This approach allowed the identification and relative quantification of around 2000 proteins and protein clusters; 196 proteins were expressed differentially, out of which 67 proteins were upregulated (Table S4) and 129 proteins were downregulated (Table S5). The major groups of differentially expressed proteins were secretion systems proteins, i.e., efflux pumps and proteins, ABC transporters and porins, but also other outer membrane components and proteins associated with antibiotic resistance (Table 5). We also identified 30 hypothetical differentially expressed proteins.

### 2.7. Upregulation of the Type VI Secretion System and RND-Type Efflux Proteins

The structural components of the type VI secretion system (T6SS), i.e., valine-glycine repeat protein G (VgrG or TssI), hemolysin-coregulated proteins (Hcp or TssD) and type VI secretion system ATPase TssH (ClpV) were dramatically upregulated in the Ab-D subpopulation (Tables S4 and S5). Interestingly, all the three out of a possible three (according to our WGS results) isoforms of the tip protein VgrG were upregulated. Likewise, the relative abundance of proteins belonging to type IV secretion system (T4SS) and Sec protein translocase complex were increased significantly in Ab-D. Additionally, the levels of proteins belonging to AdeABC and AdeIJK bacterial efflux pumps of the Resistance-nodulation-cell division (RND) superfamily transporters increased two-fold in Ab-D, while the nucleotide sequences of the corresponding contigs encoding AdeABC, AdeIJK, and MacA were identical in Ab-S and Ab-D.

### 2.8. Downregulation of Proteins Involved in Maintenance of Outer Membrane Asymmetry

All proteins identified as involved in the maintenance of the asymmetric lipid distribution, with lipopolysaccharides at the outer leaflet and phospholipids at the inner leaflet of the outer membrane, were downregulated or missing in Ab-D. These included MlaA, MlaD, and PldA involved in retrograde glycerophospholipid transport and lipid degradation [38,39], acyl-ACP-UDP-N-acetylglucosamine O-acyltransferase LpxA catalyzing the first step of lipid A biosynthesis [6], LptA and LptD lipopolysaccharide transport (Lpt) [40,41] and periplasmic carrier protein LolA [42,43]. Almost all differentially expressed proteins identified as transporters (see “Other transporters” in Table 5, Tables S4 and S5) were downregulated as well as many other outer membrane proteins (Table S5). The only exception was the C4-dicarboxylate transporter (DctA) involved in carbon metabolism, which was upregulated more than seven times in the Ab-D population. Interestingly, the ATP-dependent tetradecameric serine protease ATP-binding subunit ClpX and Lon protease (ATP-dependent protease La) were upregulated in the Ab-D subpopulation (Table 5).

### 2.9. Alterations of Other Proteins Associated with Antibiotic Resistance

We identified changes in the levels of several other proteins, earlier known to be involved in antibiotic resistance mechanisms (Table 5). We observed a significant increase in CarO (the carbapenem resistance-associated outer membrane protein) and di-hydro-pteroate synthase DHPS (the sulfonamides target [44]) expression levels in Ab-D. Three beta-lactamases, encoded by *bla*_NDM-1_, *bla*_ADC-25_, and *bla*_OXA-23_ genes (and detected by ResFinder in both Ab-S and Ab-D (Table 2)), were downregulated in the Ab-D subpopulation (Table 5). In summary, we found significant proteome alterations in Ab-D, indicating that colistin dependence is a result of a complex cellular response.

### 2.10. ISAjo2 and ISAba13 Disruption of lpxA, mlaD and pldA Genes in Colistin-Dependent A. baumannii

**Figure 4:**
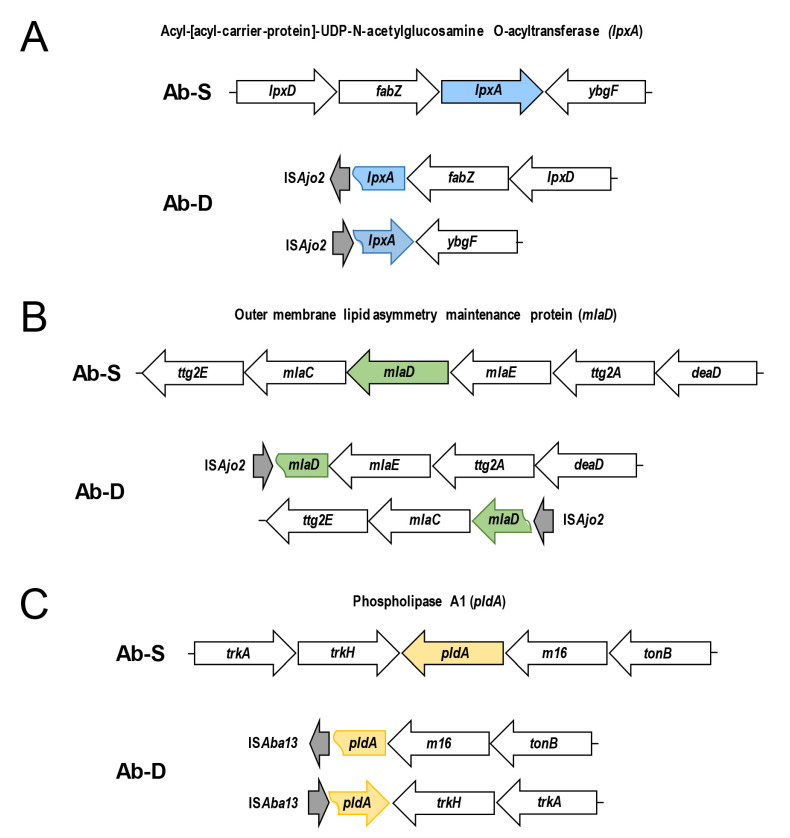
Schematic representation of IS*Ajo2* and IS*Aba13* disrupting *lpxA*, *mlaD*, and *pldA* genes in colistin-dependent *A. baumannii*. The arrangement of the gene clusters is illustrated for colistin-susceptible (Ab-S) and colistin -dependent (Ab-D) *A. baumannii*. IS*Ajo2* disrupting the *lpxA* gene (**A**) and *mlaD* gene (**B**); IS*Aba13* disrupting *pldA* (**C**). Gene clusters in Ab-S (top) are compared with corresponding disrupted Ab-D clusters (bottom). The identified insertion sequences *ISAjo2* and IS*Aba13* are shown with gray arrows. Arrows designate transcription directions of genes.

Next, we evaluated if the observed protein expression alterations were consistent with the genetic differences between Ab-S and Ab-D. Out of all significantly downregulated proteins, sequence analyses of the *lpxA*, *mlaD* and *pldA* genes in Ab-D revealed ISs elements. Thus, the IS*Ajo2*, belonging to the IS*1202* group of the ISNCY family, was inserted into both *lpxA* and *mlaD* genes, and IS*Aba13* from IS*5* family group IS*903* into the *pldA* gene, acting as gene knockouts. Figure 4 illustrates the organization of the three gene clusters containing *lpxA*, *mlaD* and *pldA* in Ab-S and Ab-D. Interestingly, that two phenotypically distinct populations, obtained from Ab-D after six subculture passages on antibiotic-free medium, contained the same ISs inserted in *mlaD* and *pldA*, but cells that returned to colistin-sensitive growth pattern (Figure 1, right) have lost the IS*Ajo2* interrupting *lpxA*.

Is elements may appear both within coding and intergenic regions of genes, where they can control neighboring genes and affecting their expression. Still, no ISs were detected in close proximity of genes encoding the three most upregulated proteins, i.e., the isoforms of the type VI secretion system tip proteins VgrG and Hcp. Pairwise sequence alignment with Stretcher (EMBL-EBI) [45] disclosed 100% identity of the VgrG- and Hcp-encoding contigs from Ab-S and Ab-D. According to Zhu et al., mutations in *katG*, encoding a catalase involved in reactive oxygen species scavenging, affected polymyxin dependence in MRD *A. baumannii* AB5075 [15] and inactivation of *mrcA*, encoding the penicillin-binding protein A1, has been suggested to promote LOS loss [7]. Nevertheless, no mutations/substitutions/insertions in *katG* or *mrcA* were present in Ab-S and Ab-D.

## 3. Discussion

### 3.1. Lipid A Modifications and LOS Deficiency in Colistin-Dependent A. baumannii

**Figure 5:**
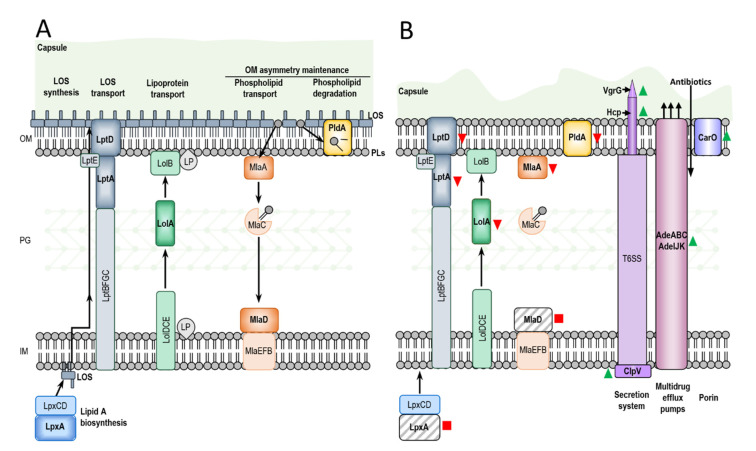
Schematic comparison of Ab-S and Ab-D illustrating a major changes occurring during the dependence process. The *A. baumannii* envelope consists of the inner membrane (IM), an aqueous periplasm containing a thin layer of peptidoglycan (PG), the outer membrane (OM) and a polysaccharide capsule. In Ab-S (**A**) LpxA catalyzes the first step of lipid A biosynthesis in the cytoplasm, and the completed lipooligosaccharide (LOS) is transported to the cell surface by the Lpt molecular machinery. The Lol system translocate lipoproteins (LP) from IM to innermost leaflet of the OM, and Mla and PldA help establish OM asymmetry, removing wrongly localized phospholipids (PLs) from the outer leaflet of OM. The capsule in Ab-D (**B**) cells is thin with surface imperfections. Ab-D cells exhibit a LOS-deficient phenotype (**B**), where PLs occupy both leaflets of the OM, and lipid A is missing due to the IS disruption of *lpxA* gene. The lipopolysaccharide transporters LptA and LptD, OM asymmetry-maintaining proteins MlaA, MlaD, and PldA and periplasmic chaperone LolA downregulated in comparison to Ab-S or missing. The VgrG, Hcp and ClpV, components of the type 6 secretion system (T6SS) and several Ade components of the RND-type efflux pumps, as well as the carbapenem susceptibility porin or CarO, were significantly upregulated in Ab-D. Red squares, red arrows pointing down, green arrows pointing up indicate lost, downregulated and upregulated proteins, respectively.

The present study showed insertion of IS*Ajo2*, which belongs to the IS*1202* group of the ISNCY family, into *lpxA* gene in the colistin-dependent subpopulation Ab-D (Figure 4A). Insertional disruption of the *lpxA* gene knocked it out and abolished acyl-ACP-UDP-N-acetylglucosamine O-acyltransferase expression in Ab-D (Table 5 and Table S5), which catalyzes the first step of lipid A biosynthesis. This event entirely terminates lipid A biosynthesis and results in a LOS-deficient *A. baumannii* phenotype [6] (Figure 5), which we confirmed here *(*Figure 2 and Figure 3). Indeed, ISs and other defects in the *lpxACD* gene system are known to be associated with both colistin resistance [32,33] and dependence [34]. In the latter case, IS*Aba1* was disrupting the *lpxC* gene encoding UDP-3-O-acyl-N-acetylglucosamine deacetylase [34]. IS*Ajo2* was earlier identified in MDR *A. johnsonii*, where two copies of IS*Ajo2* flanked the region containing the carbapenemase gene *bla*_OXA-58_ [36]. We also noted that IS*Ajo2* was much more abundant in the Ab-D than in Ab-S (Table 4). This difference might be explained either by a redundancy of the genome assembly, or by IS*Ajo2* transposition triggered by subinhibitory concentrations of colistin [31]. In addition, both Ab-S and Ab-D displayed a high number of ISs elements accumulated in their genomes (Table 3), which likely reflects the evolutionary history of the parent strain.

We identified two other genes inactivated by insertions, i.e., *mlaD* and *pldA*, encoding proteins involved in maintenance of OM asymmetry [38] (Figure 5). In Ab-D, the IS*Ajo2* insertion interrupted the *mlaD* gene (Figure 4B), encoding a membrane-anchored periplasmic protein MlaD of the Mla pathway, mediating retrograde transport of glycerophospholipids [46]. IS*Aba13* from the IS5 family group IS*903* was located in the *pldA* gene (Figure 4C). IS*Aba13* has previously been found in carbapenem-resistant *A. baumannii* [47,48] upstream from *bla*_OXA-94_ [48]. PldA is an outer membrane phospholipase catalyzing the hydrolysis of acyl ester bonds in phospholipids and degradation of mislocalized phospholipids from the surface-exposed outer leaflet. It was hypothesized that Mla and PldA systems function independently to prevent accumulation of phospholipids at the cell surface through distinct, yet related mechanisms [49] (Figure 5). We also verified *mlaD* and *pldA* inactivation and downregulation of MlaA in Ab-D by comparative proteomic analysis (Table 5 and Table S5). Our results are in agreement with the findings by Powers and Trent [38] on how LOS-deficient and highly polymyxin B-resistant *A. baumannii* can improve their fitness by elimination of the two OM asymmetry-maintenance systems, Mla and PldA. This was discovered, however, in colistin-resistant *A. baumannii* obtained after 120 generations of passaging on 10 µg/mL polymyxin B, while we observed the emergence of phenotypically colistin-dependent subpopulation already after 8 passages on colistin [38]. Recently, a transcriptomic analysis of a colistin-dependent isolate in relation to its parent colistin-susceptible MRD *A. baumannii* AB5075 revealed 1.7-fold increased expression of *mlaD* and 2.0-fold decreased expression of *pldA* in colistin-dependent bacteria [15]. We believe that this discrepancy can be attributed to the difference between strains.

Various studies have indicated that, beside downregulation of glycerophospholipid transport and lipid degradation, lack of LOS can also lead to altered expression of critical transport and biosynthesis systems, modulating the composition and structure of the bacterial envelope [50], which is in agreement with our observations (Figure 2), and reducing the capacity for adhesion and formation of biofilms [51,52]. We found that in LOS-deficient Ab-D, many other proteins involved in maintenance of the asymmetric lipid distribution were downregulated or missing. This included proteins LolA from the Lol lipoprotein transport system, LptA and LptD lipopolysaccharide transport proteins responsible for the transport and assembly of LPS [40,41] and many other transporters and outer membrane proteins (Table 5 and Table S5).

### 3.2. Upregulation of the Type VI Secretion System, Efflux Proteins and Proteases in Colistin-Dependent A. baumannii

The most dramatic protein upregulation in LOS-deficient Ab-D population of *A. baumannii* was attributed to secretion system and efflux proteins (Figure 5). These proteins help bacteria to export specific bacterial products to the cell surface, the extracellular environment and to other bacteria or eukaryotic cells, thereby promoting bacterial virulence [53]. Gram-negative bacteria harbor multiple types of secretion systems and T6SS is the most prevalent. T6SS is a macromolecular envelope-spanning apparatus that injects toxic bacterial effector proteins directly into eukaryotic target host or other prokaryotic cells, which is important for virulence and inter-bacterial competition. It mimics inverted phage tail and tube [54].

The inner tube of this structure is comprised of hemolysin-coregulated protein (Hcp or TssD) assembled into stacked hexameric rings, permitting effectors to pass through the tube center. This inner tube is capped by the valine-glycine repeat protein G (VgrG or TssI) trimer, forming the needle-like structure on the top of the inner tube allowing it to penetrate the host membrane. It is surrounded by a contractile sheath, which is disassembled and recycled by an AAA+-type ATPase TssH (ClpV) after the delivery of toxic effectors into a recipient cell. The perturbation of the cell envelope caused by membrane-targeting antibiotics, such as polymyxin B, was earlier reported to serve as the signal triggering the T6SS activation in Gram-negative bacteria [55]. Nevertheless, a repressed expression of T6SS genes observed for LOS-deficient colistin-resistant *A. baumannii* [50], which is opposite to our findings. We suggest that the upregulation of T6SS in the LOS-deficient colistin-dependent population of *A. baumannii* may be a part of a cellular response to the LOS-deficiency caused by exposure to colistin.

In addition, we observed an upregulation of RND superfamily transporters: AdeABC and AdeIJK bacterial efflux pumps (Figure 5). Overproduction of these in clinical isolates is associated to MDR due to their very broad substrate profiles [56,57,58,59,60,61]. In particular, they play a major role in tigecycline resistance in *A. baumannii*, where AdeIJK is able to efflux β-lactams. Nevertheless, we observed that the Ab-D subpopulation displayed increased sensitivity to carbapenems, tigecycline, piperacillin-tazobactam, and amoxicillin-clavulanic acid compared to the Ab-S, which could be due to increased OM permeability.

Overexpression of AdeABC or AdeIJK may also affect the expression of various proteins involved in adhesion and biofilm formation (72). In LOS-deficient *A. baumannii* increased expression of *adeIJK* and *macAB-tolC* has been attributed to an intracellular accumulation of toxic substances [50]. Moreover, in *E. coli* and *Salmonella enterica* cells with higher *acrAB* expression (AcrA corresponds to AdeI, in *A. baumannii*), higher spontaneous mutation frequencies were observed in response to ciprofloxacin, tetracycline, and chloramphenicol, indicating involvement of AcrAB efflux pump in initial stages of permanent antibiotic resistance [62].

Interestingly, the ATP-dependent tetradecameric serine protease ATP-binding subunit ClpX and the Lon protease (ATP-dependent protease La) were found among proteins upregulated in the colistin-dependent subpopulation (Table S1). These energy-dependent proteases, also functioning as chaperones, are involved in degradation of misfolded, damaged and short-lived regulatory proteins playing a significant role in bacterial stress response [63] and virulence regulation [64,65].

## 4. Materials and Methods

### 4.1. Bacterial Cultivation and Antibiotic Susceptibility Testing

The *A. baumannii* clinical isolate was obtained from the abdominal cavity of a patient during 2013 at the Department of Clinical Microbiology, Linköping University Hospital, Sweden and the susceptibility profile of this parental strain named AB1 was earlier characterized by Nordqvist et al. [66]. To test for colistin resistance, bacterial suspensions with 5 × 10^5^ CFU/mL (colony forming units/mL), or 0.5 McFarland units in 0.9% NaCl were grown for 24 h at 37 °C on Mueller-Hinton agar (MHA) (Becton Dickinsson, Franklin Lakes, NJ, USA) with Etest strips (BioMérieux, Marcy l’Etoile, France) covering colistin concentrations between 0.016 and 256 μg/mL. The *A. baumannii* CCUG 19,096 strain (Culture Collection, Göteborg University, Sweden) was used as a control for the susceptibility test [67]. For selection of a colistin-dependent subpopulation, the isolate was cultured for 8-10 passages on agar containing 16 µg/mL colistin sulphate according to [66]. (Merk Sigma Aldrich, St. Louis, MO, USA) and parallel colistin-Etests to follow the growth pattern. Two *A. baumannii* subpopulations, susceptible to colistin (Ab-S) and colistin-dependent (Ab-D), were grown on MHA plates or MHA plates with a colistin Etest strip, collected and further processed for analyses on transmission electron microscopy (TEM). For lipid analyses with matrix-assisted matrix desorption-ionization (MALDI-TOF) MS, WGS and proteomics, Ab-S and Ab-D subpopulation were grown on MHA plates with colistin Etest strips. In addition, Ab-D subpopulation was cultured for 6 passages on Columbia agar base medium and further applied for colistin Etest to monitor the growth pattern.

The minimum inhibitory concentrations (MICs) were measured for 17 antimicrobial agents, including colistin, meropenem, ertapenem, amikacin, gentamicin, tobramycin, ciprofloxacin, trimethoprim-sulfamethoxazole, ertapenem, cefotaxime, ceftazidime, ceftazidime-avibactam, ceftolozane-tazobactam, piperacillin-tazobactam, amoxicillin-clavulanic acid, aztreonam, and tigecycline were evaluated using the broth microdilution method with Sensititre Gram-negative MIC DKMGN plates (Thermo Fisher Scientific, Waltham, MA, USA). The MIC values were interpreted according to the current European Committee on Antimicrobial Susceptibility Testing (EUCAST) clinical breakpoints.

### 4.2. Transmission Electron Microscopy

Bacteria were collected, washed, fixed in 3% glutaraldehyde (Polysciences, Warrington, PA, USA) in 0.1 M Na cacodylate buffer, pH 7.4 for 30 min at room temperature (RT), centrifuged and embedded in 4% gelatin. Then followed 3% glutaraldehyde fixation in sodium cacodylate buffer pH 7.4 for 2 h at RT, washing with the same buffer and post-fixation in 1% osmium tetroxide (Polysciences) for 1 h at 4 °C, rinsing and staining with 2% uranyl acetate (Polysciences in 50% ethanol and dehydration in a series of ascending concentrations of ethanol and acetone. Prior to embedding in Durcupan ACM epoxy embedding medium kit (Merk Sigma Aldrich) two-step infiltration was done. Ultrathin sections of 70-nm thickness were prepared using a Leica EM UC7 ultramicrotome (Leica Microsystems GmbH, Wetzlar, Germany), collected onto formvar-coated slot grids, and counter-stained with uranyl acetate and lead citrate. The specimens were examined in a JEM 1230 transmission electron microscope operated at 100 kV (JEOL Ltd., Tokyo, Japan), and the images were taken with a Orius SC1000 CCD camera using Digital Micrograph software (Gatan, Pleasanton, CA, USA).

For measuring the bacterial capsule thickness, images of the cells were analyzed with the ImageJ software (NIH, Bethesda, MD, USA), yielding graphs with means (±SE) and statistical analyses based on paired two-tailed Student’s *t*-tests. *p*-values < 0.05 (*), < 0.01 (**), and < 0.001 (***) were considered significant. At least 3 independent experiments were performed on separate days on different cell passages.

### 4.3. Lipid A Extraction

Bacteria were collected and lipid A was extracted by an improved rapid microextraction method previously described [34,68], with some modifications. Briefly, 10 mg of cells suspended in 100 μL of isobutyric acid −1 M ammonium hydroxide mixture (5:3, *v*/*v*) were incubated in a microwave oven for 60 s (400 W, 2450 MHz) and then centrifuged at 8000 g for 15 min. Supernatants were transferred to new tubes, mixed with an equal volume of water and vacuum dried. The obtained pellets were washed twice in 400 μL of methanol, and centrifuged at 5000 rpm for 15 min. Finally, the insoluble lipid A was solubilized in 100 μL chloroform-methanol-water mixture (3:1.5:0.25, *v*/*v*/*v*) and subjected to a mass spectrometry analysis.

### 4.4. Matrix-Assisted Laser Desorption and Ionization Mass Spectrometry

1 μL of lipid A sample was diluted with an equal volume of 5-chloro-2-mercaptobenzothiazole (CMBT)-EDTA matrix (20 mg/mL of CMBT in chloroform-methanol-water [4:4:1, *v*/*v*/*v*], 20 mM EDTA ammonium salt) as described in [69]. 0.5 μL of this mixture was loaded onto a MALDI target plate. Data were acquired with an ultrafleXtreme MALDI TOF mass spectrometer (Bruker Daltonics, Billerica, MA, USA) operated with the FlexControl software (version 3.4, Bruker Daltonics) in negative reflector mode.

### 4.5. Whole-Genome Sequencing and Data Analysis

DNA was extracted from the isolates using EZ1 DNA Tissue Kit (Qiagen, Hilden, Germany). After quantification with Qubit dsDNA High Sensitivity kit (Thermo Fisher Scientific), 20 ng of DNA was used for library preparation using QIAseq FX DNA Library Kit (Qiagen). DNA libraries were sequenced on the MiSeq platform (Illumina, San Diego, CA, USA) with 2 × 300 bp paired-end reads, and the samples obtained an average sequencing depth of 103×. Data analysis was performed in CLC Genomics Workbench v. 9.5.4 with the Microbial Genomics Module v. 1.6.2 (Qiagen). The analysis included determination of and mapping to the closest NCBI reference genome (NC_011586), multi locus sequence typing (MLST) with the Pasteur scheme [70] and *de novo* assembly. Assembled data were searched for antimicrobial resistance genes against the ResFinder database (Centre for Genomic Epidemiology, Technical University of Denmark) with a threshold of 98% for identity and 60% for length [28]. A bacterial plasmid database PLSDB (https://ccb-microbe.cs.uni-saarland.de/plsdb/, accessed on 23 June 2020) version v0.4.1-2-g1b893f22b9 [30] with maximal *p*-value set to 0.1 and minimal identity set to 0.99 was used for identification of plasmids. Winner-takes-all strategy that removes redundancy from the output was employed (hashes found in multiple queries were removed except for the query with highest identity). ISs were annotated using ISFinder (https://isfinder.biotoul.fr/, accessed on 4 May 2020) [35] with E-value threshold below 1 × 10^−4^ for *Blast* hits. The global alignment tools Needle and Stretcher (EMBL-EBI, https://www.ebi.ac.uk/Tools/psa/, accessed on 8 March 2018) [45] were used for pairwise sequence alignment. Variant calling was first performed against the closest reference genome, and single nucleotide polymorphisms (SNPs) with a depth of coverage ≥ 20× and a frequency ≥90% were compared between the two subpopulations.

### 4.6. Database

The WGS nucleotide sequences of *A. baumannii* were translated in silico. The open reading frames (ORFs) prediction for potential protein encoding segments has been carried out using ORF Finder [71] from the National Center for Biotechnology Information (NCBI) with the following settings: bacterial, archaeal & plant plastid code; ATG and alternative start codons, minimal length 75 nucleotides. The generated FASTA-style databases had genomic position and frame information embedded into each header tag for a given sequence.

### 4.7. Protein Extraction

Bacterial cells collected from the agar plates were suspended in 50 mL phosphate buffered saline (PBS) pH 7.4, and washed twice by centrifugation at 3500× *g* for 20 minat 4 °C. The obtained pellets were resuspended with 0.5 mL lysis buffer (1 mM EDTA, pH 7.4, 2% SDS, 40 mM DTT in PBS) with 0.5 μL Pierce Universal Nuclease (Thermo Fisher Scientific, Waltham, MA, USA) and 5 μL Halt Protease Inhibitor Cocktail (Thermo Fisher Scientific) added. The homogenates were sonicated in an Ultrasonic disintegrator, Soniprep 150 (MSE, London, UK), operating at 20% duty cycle and 3−4 output for 2 min. Lysates were clarified by centrifugation at 15,000× *g* for 30 min at 4 °C. Protein samples were processed by a filter-aided sample preparation (FASP) method according to Wisniewski [72]. The digested peptide samples were vacuum dried, dissolved in 0.1% formic acid and the peptide concentration was estimated by absorbance measurements at 280 nm by NanoDrop (Thermo Fisher Scientific) prior to liquid chromatography-tandem mass spectrometry (LC–MS/MS) analyses. The proteome assessments were repeated at least 10 times.

### 4.8. Proteomic Analysis by LC-MS/MS

Peptides were separated by reverse phase chromatography on a 20 mm × 100 μm C18 pre-column followed by a 100 mm × 75 μm C18 column with particle size 5 μm (NanoSeparations, Nieuwkoop, Netherlands) at a flow rate of 300 nL/min on EASY-nLC II (Thermo Fisher Scientific) by a gradient of 0.1% formic acid in water (A) and 0.1% formic acid in acetonitrile (B) as follows: from 2% B to 30% B in 60 min; from 30% B to 100% B in 60 min. Automated online analyses were performed in positive mode by LTQ Orbitrap Velos Pro hybrid mass spectrometer (Thermo Fisher Scientific) equipped with a nano-electrospray source with Xcalibur software (v.2.6, Thermo Fisher Scientific). Full MS scans were collected with a range of 350–1800 *m*/*z*, a resolution of 30,000 (*m*/*z* 200), the top 20 most intense multiple charged ions were selected with an isolation window of 2.0 and fragmented in the linear ion trap by collision-induced dissociation with normalized collision energy of 30%. Dynamic exclusion was enabled ensuring peaks selected for fragmentation were excluded for 60 s.

### 4.9. Database Searching

The generated raw files were analyzed using Sequest HT in Proteome Discoverer (Thermo Fisher Scientific, San Jose, CA, USA, CS version 1.4.0.288) and the translated genome sequences based on WGS. The following search parameters were used: trypsin as a digestion enzyme; maximum number of missed cleavages 2; fragment ion mass tolerance 0.50 Da; parent ion mass tolerance 10.0 ppm; carbamidomethylation of cysteine as fixed modification and methionine oxidation as variable modifications.

### 4.10. Data Evaluation and Label-Free Quantification

Identified proteins were validated using SCAFFOLD software (Version 4.4.8; Proteome Software Inc., Portland, OR, USA). Identifications were based on a minimum of 2 peptides, minimum 95% peptide identification probability (using the Scaffold Local FDR algorithm), and minimum 99% protein identification probability using the Protein Prophet algorithm [73]. Proteins, which contained similar peptides, and which could not be differentiated based on LC-MS/MS analysis alone were grouped to satisfy the principles of parsimony. The label-free quantitative analysis was performed using total number of spectral counts; normalization was performed to account for variations between samples. Quantitative differences were statistically analyzed by Student’s *t*-test with the Benjamini-Hochberg correction. Differences with *p*-values ≤ 0.05 were considered statistically significant.

### 4.11. Protein Homology Search

Protein sequences were annotated with search in the NCBI database using the Basic Local Alignment Search Tool (BLAST, National Center for Biotechnology Information, Bethesda, MD, USA) blastp algorithm and Uniprot BLAST (EMBI-EBI, Cambridgeshire, UK) software (https://www.uniprot.org/, accessed on 20 May 2020).

### 4.12. Availability of Data and Materials

The raw WGS data has been deposited in the NCBI Sequence Read Archive (SRA) (http://www.ncbi.nlm.nih.gov/sra) and can be accessed through the accession PRJNA657148.

The mass spectrometry data has been deposited to the ProteomeXchange Consortium (www.proteomexchange.org) via the PRIDE [74] partner repository with the dataset identifier PXD020218.

## 5. Conclusions

We here performed molecular and cellular profiling of colistin-susceptible *A. baumannii* isolate, harboring a variety of resistance genes, and its colistin-dependent subpopulation, providing new insights into colistin dependence. We also demonstrated, for the first time, the potential involvement of IS*Ajo2* and IS*Aba13* insertion sequences in colistin dependence, confirming a role for ISs in colistin treatment. Our results suggest that colistin dependence in *A. baumannii* is likely the result of complex cellular events that occur concurrently with ISs-triggered LOS-deficiency.

## Figures and Tables

**Table 1:** Whole genome sequencing (WGS) of Ab-S and Ab-D subpopulations of *A. baumannii*.

Subpopulation	Ab-S	Ab-D
Genome length	4,111,821 bp	4,125,623 bp
Reference *	NC_011586	NC_011586
Mapped reads *	95.1%	90.9%
Unmapped **	4.9%	9.1%
Contigs	121	186

* Complete genome sequences were searched against the NCBI bacteria database in order to determine the species. The reference genome with the best match is gathered and mapped reads refers to the percentage of the genome that have been matched against the reference genome. ** Unmapped percentage are reads that did not map against any previous reference genomes in NCBI.

**Table 2:** Antimicrobial resistance genes revealed by ResFinder analysis in *A. baumannii*.

Resistance Genes *	Predicted Resistance Phenotype	Enzyme Class/Family	Resistance Mechanism
*aac(3)-IId*	Aminoglycosides	Aminoglycoside-modifying enzymes	Enzymatic inactivation of antibiotic
*aph(3′)-VIa*			
*aph(3′)-Ic*			
*aac(3)-Ia*			
*aadA1*			
*bla_OXA-69_*	Beta-lactams	Beta-lactamases	Enzymatic inactivation of antibiotic
*bla_TEM-1D_*			
*bla_OXA-23_*			
*bla_ADC-25_*			
*bla_NDM-1_*			
*floR*	Phenicols	Major facilitator superfamily antibiotic efflux pump	Antibiotic efflux
*catA1*		chloramphenicol acetyltransferase	Enzymatic inactivation of antibiotic
*sul1*	Sulphonamides	Dihydropteroate synthase	Antibiotic target replacement
*tetA*	Tetracycline	Antibiotic efflux pump	Antibiotic efflux

* The found resistance genes are all the matches against Resfinder, meaning all the known resistance genes in the database of the Centre of Epidemilogy. The threshold for a hit of the resistance genes are 98% similarity and 60% length.

**Table 3:** Number of IS families found by ISFinder in Ab-S and Ab-D.

ISs Family	ISs Number	
	Ab-S	Ab-D
IS*5*	108	129
IS*6*	97	97
IS*4*	52	61
IS*3*	36	36
ISNCY	33	219
Tn3	33	31
IS*30*	24	25
IS*L3*	22	19
IS*1*	14	14
IS*66*	12	11
IS*256*	4	4
IS*1182*	3	3
IS*481*	3	3

**Table 4:** Most abundant IS elements found by ISFinder in Ab-S and Ab-D.

IS	IS Family	Group	Origin	ISs Number	
				Ab-S	Ab-D
IS*Ajo2*	ISNCY	IS*1202*	*Acinetobacter johnsonii*	18	84
IS*Aba1*	IS*4*	IS*10*	*Acinetobacter baumannii*	26	31
IS*Aba13*	IS*5*	IS*903*	*Acinetobacter baumannii*	16	19
IS*Aba125*	IS*30*		*Acinetobacter baumannii*	9	10
Tn*As3*	Tn*3*		*Aeromonas salmonicida*	7	7
IS*1008*	IS*6*		*Acinetobacter calcoaceticus*	6	7
IS*Alw4*	IS*3*	IS*51*	*Acinetobacter lwoffii*	2	2

**Table 5:** Selected groups of proteins differentially expressed in Ab-S and Ab-D. Fold changes were calculated with Ab-S as the reference category. Fold change value 0 means that protein was detected in Ab-S, but not in Ab-D.

Proteins	Accession Number (NCBI)	Fold Change	*p*-Value
**Secretion systems proteins**			
type VI secretion system tip protein VgrG	WP_161283681.1	12	0.0055
type VI secretion system tip protein VgrG	WP_000935013.1	8.3	0.00025
type VI secretion system tip protein VgrG	WP_057691008.1	6.3	0.0008
type VI secretion system tube protein Hcp	WP_000653195.1	5.9	0.0035
type VI secretion system ATPase TssH (ClpV)	WP_000987834.1	2.8	<0.00010
type IV secretion protein Rhs	WP_000081475.1	4.9	<0.00010
type IV secretion protein Rhs	ACJ40183.1	3	0.00018
type III secretion system protein EcsC	EXE73502.1	0.5	0.00019
protein translocase subunit SecF	WP_001985897.1	2.3	0.0036
rhombotarget A	WP_000920020.1	0.1	0.0012
Adhesin/BapA prefix-like domain-containing protein	KRJ95188.1/WP_000196831.1	0.5	0.00023
**Efflux proteins**			
AdeC/AdeK/OprM family multidrug efflux complex outer membrane factor	WP_000010517.1	4.3	0.0035
AdeB family multidrug efflux RND transporter permease subunit	WP_000046678.1	2.7	<0.00010
multidrug efflux RND transporter periplasmic adaptor subunit AdeI (AcrA)	WP_000986589.1	2.1	<0.00010
multidrug efflux RND transporter permease subunit AdeB	WP_000987602.1	2	0.0011
multidrug efflux RND transporter AdeIJK outer membrane channel subunit AdeK	WP_001174793.1	2	0.0021
multidrug efflux RND transporter periplasmic adaptor subunit AdeA	WP_001169096.1	1.9	0.0026
AdeT RND type efflux pump	ADX01640.1	0.4	0.00021
MacA family efflux pump subunit	WP_001124213.1	INF	<0.00010
putative RND family drug transporter	CAJ77853.1	0.3	0.00013
putative RND family drug transporter (outer membrane efflux protein)	CAJ77861.1	0	0.006
**Outer membrane asymmetry maintenance**			
phospholipase A1 (PldA)	EGJ62971.1	0.04	<0.00010
outer membrane lipid asymmetry maintenance protein MlaD	WP_098732046.1	0	<0.00010
acyl-ACP-UDP-N-acetylglucosamine O-acyltransferase (LpxA)	WP_031976200.1	0	<0.00010
putative lipopolysaccharide transport protein A (ABC superfamily peri_bind) (LptA)	CAM87332.1	0.4	0.0049
LPS-assembly protein LptD	WP_045544211.1	0,6	0.0046
outer membrane lipoprotein chaperone LolA	WP_001056757.1	0.5	0.00024
VacJ family lipoprotein (MlaA)	WP_001109851.1	0.4	0.0026
**Other transporters**			
TonB-dependent receptor	WP_000413997.1	0.5	<0.00010
putative TonB-dependent Outer membrane receptor for vitamin B12/cobalamin transport (Btub)	CAM85573.1	0.5	<0.00010
dicarboxylate/amino acid:cation symporter (C4-dicarboxylate transporter DctA)	WP_000347180.1	7.4	0.00023
autotransporter domain-containing protein	WP_001260880.1	0.1	0.00025
amino acid ABC transporter substrate-binding protein	WP_052137106.1	0.3	<0.00010
phosphate ABC transporter	EGJ58385.1	0.1	<0.00010
molybdate ABC transporter substrate-binding protein	WP_000253153.1	0	0.0018
toluene tolerance protein Ttg2A/ABC transporter ATP-binding protein	EEX02911.1/WP_002135589.1	0.3	0.0059
**Proteases**			
ATP-dependent Clp protease ATP-binding subunit ClpX	WP_001289250.1	2.9	<0.00010
Lon protease	AEP05596.2	1.6	0.00027
**Proteins associated with drug resistance**			
carbapenem susceptibility porin CarO	WP_000733010.1	3.9	0.002
dihydropteroate synthase DHPS	AFB76381.1	2.5	0.0042
beta-lactamase (*bla_ADC-25_*)	AEP07218.1	0.4	<0.00010
MBL fold metallo-hydrolase (*bla_NDM-1_*)	WP_000732912.1	0.3	<0.00010
OXA-23 carbapenemase (*bla_OXA-23_*)	VCZ51052.1	0.7	0.00053

## Data Availability

The raw WGS data has been deposited in the NCBI Sequence Read Archive (SRA) (http://www.ncbi.nlm.nih.gov/sra) and can be accessed through the accession PRJNA657148. The mass spectrometry data has been deposited to the ProteomeXchange Consortium (www.proteomexchange.org) via the PRIDE [74] partner repository with the dataset identifier PXD020218.

## Supplementary Materials

The following are available online at https://www.mdpi.com/1422-0067/22/2/576/s1, Table S1. Minimum inhibitory concentrations (μg/mL) of Ab-S and Ab-D subpopulations. Table S2. PLSDB analysis for plasmids in Ab-S and Ab-D subpopulations of *A. baumannii*. Table S3. ISs annotation by ISFinder Ab-S and Ab-D subpopulations of *A. baumannii*. Table S4. Proteins upregulated in Ab-D subpopulation in comparison to Ab-S. Fold changes were calculated with Ab-S as the reference category, which implies that values over 1 mean that a protein is upregulated in Ab-D. Table S5. Proteins downregulated in Ab-D in comparison to Ab-S. Fold changes were calculated with Ab-S as the reference category and values below 1 indicate protein downregulation in Ab-D. Supplementary Data File S1. The file contains Ab-S nucleotide sequence database. Supplementary Data File S2. The file contains Ab-D nucleotide sequence database. Supplementary Data File S3. The file contains Ab-S protein sequence database. Supplementary Data File S4. The file contains Ab-D protein sequence database.

## Abbreviations

WGS	Whole genome sequencing
LC	liquid chromatography
MS/MS	tandem mass spectrometry
TEM	transmission electron microscope
MALDI-TOF	Matrix Assisted Laser Desorption Ionization-Time Of Flight
OM	outer membrane
MDR	multidrug resistance
XDR	extensively drug-resistant
LPS	lipopolysaccharide
LOS	lipooligosaccharide
T6SS	type VI secretion system
ISs	insertion sequence
RND	resistance-nodulation-division
MIC	minimum inhibitory concentration
ORFs	open reading frames
