# Supplementary material for: Colistin Dependence in Extensively Drug-Resistant Acinetobacter baumannii Strain Is Associated with ISAjo2 and ISAba13 Insertions and Multiple Cellular Responses
Source: Int J Mol Sci. 2021 Jan 8;22(2):576. doi: 10.3390/ijms22020576 (PMC7827689; doi:10.3390/ijms22020576)
Supplement: Supplementary file 1 [file ijms-22-00576-s001.zip › Table S1.docx]

Table S1

Minimum inhibitory concentrations (mg/L) of Ab-S and Ab-D subpopulations.

|  | Ab-S  MIC mg/L | Ab-S  S/I/R | Ab-D  MIC mg/L | Ab-D  S/I/R | EUCAST species related breakpoint  S/R mg/L | EUCAST PK/PD breakpoint  S/R mg/L |
| --- | --- | --- | --- | --- | --- | --- |
| **Antibiotic** |  |  |  |  |  |  |
| **Polymyxins** |  |  |  |  |  |  |
| Colistin | ≤ 2 | S | >8 | R | ≤2/>2 | x |
|  |  |  |  |  |  |  |
| **Carbapenems** |  |  |  |  |  |  |
| Meropenem | >16 | R | 8 | I | ≤2/>8 | x |
| Imipenem | >16 | R | 4 | I | ≤2/>4 | x |
| Ertapenem | >2 | R | 2 | R | x | ≤0,5/>0,5 |
|  |  |  |  |  |  |  |
| **Aminoglycoside** |  |  |  |  |  |  |
| Amikacin | >32 | R | 16 | R | ≤8/>8 | x |
| Gentamicin | >8 | R | >8 | R | ≤4/>4 | x |
| Tobramycin | >8 | R | >8 | R | ≤4/>4 | x |
|  |  |  |  |  |  |  |
| **Extended spectrum cephalosporins/**  **β-lactamase inhibitors** |  |  |  |  |  |  |
| Cefotaxime | >8 | R | >8 | R | x | ≤1/>2 |
| Ceftazidime | >16 | R | >16 | R | x | ≤4/>8 |
| Ceftazidime-avibactam | >16 | R | 16 | R | x | ≤8/>8 |
| Ceftolozane-tazobactam | >32 | R | >32 | R | x | ≤4/>4 |
|  |  |  |  |  |  |  |
| **Penicillins/β-lactamase inhibitors** |  |  |  |  |  |  |
| Piperacillin-tazobactam | >32 | R | <1 | S | x | ≤4/>16 |
| Amoxicillin- clavulanic acid | >64 | R | 32 | R | x | ≤2/>8 |
|  |  |  |  |  |  |  |
| **Monobactam** |  |  |  |  |  |  |
| Aztreonam | >32 | R | 32 | R | x | ≤4/>8 |
|  |  |  |  |  |  |  |
| **Antipseudomonal fluoroquinolones** |  |  |  |  |  |  |
| Ciprofloxacin | >2 | R | >2 | R | ≤0,001/>1 | x |
|  |  |  |  |  |  |  |
| **Glycylcycline/tetracyclines** |  |  |  |  |  |  |
| Tigecycline | 1 | R | 0,5 | S | x | ≤0,5/>0,5 |
|  |  |  |  |  |  |  |
| **Folate pathway inhibitors** |  |  |  |  |  |  |
| Trimethoprim- sulfamethoxazole | >8 | R | >8 | R | ≤2/>4 | x |
