# Supplementary material for: Colistin Dependence in Extensively Drug-Resistant Acinetobacter baumannii Strain Is Associated with ISAjo2 and ISAba13 Insertions and Multiple Cellular Responses
Source: Int J Mol Sci. 2021 Jan 8;22(2):576. doi: 10.3390/ijms22020576 (PMC7827689; doi:10.3390/ijms22020576)
Supplement: Supplementary file 1 [file ijms-22-00576-s001.zip › Table S2.pdf]

Plasmids in Ab-S

identity	shared_hashes	median_multiplicity	pvalue	ACC_NUCCORE	Topology_NUCCORE	Location_BIOSAMPLE	loc_lat	loc_lng	IsolationSource_BIOSAMPLE	Host_BIOSAMPLE	SamplType_BIOSAMPLE	Length_NUCCORE	GC_NUCCORE	taxon_name	taxon_genus_name	taxon_family_name	taxon_order_name	taxon_class_name	taxon_phylum_name	D1	D2
1.0	1000	1	0	JQ739158.1	circular							4797	65.66604127579737	*Acinetobacter lwoffii*	*Acinetobacter (469)*	*Moraxellaceae (468)*	*Pseudomonadales (72274)*	Gammaproteobacteria (1236)	Proteobacteria (1224)	-2.5375497000000005	3.3951879999999997
0.9996659999999999	993	1	0	NZ_CP027244.1	circular	China: Sichuan: Chengdu	30.6765553	104.0612783		*Homo sapiens*	Pure Culture	8731	34.37177871950521	*Acinetobacter baumannii*	*Acinetobacter (469)*	*Moraxellaceae (468)*	*Pseudomonadales (72274)*	Gammaproteobacteria (1236)	Proteobacteria (1224)	-3.7209535000000002	3.5790975
0.9995700000000001	991	1	0	NZ_CP031382.1	circular	Italy: Rome	41.8857	12.5024	cerebrospinal fluid	*Homo sapiens*		70101	33.48311721658749	*Acinetobacter baumannii ACICU*	*Acinetobacter (469)*	*Moraxellaceae (468)*	*Pseudomonadales (72274)*	Gammaproteobacteria (1236)	Proteobacteria (1224)	-8.030564	-7.0007199999999999
0.994943	899	1	0	KF220658.1	circular							1634	57.28274173806609	*Klebsiella pneumoniae*	*Klebsiella (570)*	*Enterobacteriaceae (543)*	*Enterobacterales (91347)*	Gammaproteobacteria (1236)	Proteobacteria (1224)	-2.5569785	3.3618129999999997

Plasmids in Ab-D

identity	shared_hashes	median_multiplicity	pvalue	ACC_NUCCORE	Topology_NUCCORE	Location_BIOSAMPLE	loc_lat	loc_lng	IsolationSource_BIOSAMPLE	Host_BIOSAMPLE	SamplType_BIOSAMPLE	Length_NUCCORE	GC_NUCCORE	taxon_name	taxon_genus_name	taxon_family_name	taxon_order_name	taxon_class_name	taxon_phylum_name	D1	D2
1.0	1000	1	0	JQ739158.1	circular							4797	65.66604127579737	*Acinetobacter lwoffii*	*Acinetobacter (469)*	*Moraxellaceae (468)*	*Pseudomonadales (72274)*	Gammaproteobacteria (1236)	Proteobacteria (1224)	-2.5375497000000005	3.3951879999999997
0.9996659999999999	993	1	0	NZ_CP027244.1	circular	China: Sichuan: Chengdu	30.6765553	104.0612783		*Homo sapiens*	Pure Culture	8731	34.37177871950521	*Acinetobacter baumannii*	*Acinetobacter (469)*	*Moraxellaceae (468)*	*Pseudomonadales (72274)*	Gammaproteobacteria (1236)	Proteobacteria (1224)	-3.7209535000000002	3.5790975
0.9995700000000001	991	1	0	NZ_CP031382.1	circular	Italy: Rome	41.8857	12.5024	cerebrospinal fluid	*Homo sapiens*		70101	33.48311721658749	*Acinetobacter baumannii ACICU*	*Acinetobacter (469)*	*Moraxellaceae (468)*	*Pseudomonadales (72274)*	Gammaproteobacteria (1236)	Proteobacteria (1224)	-8.030564	-7.0007199999999999
0.994943	899	1	0	KF220658.1	circular							1634	57.28274173806609	*Klebsiella pneumoniae*	*Klebsiella (570)*	*Enterobacteriaceae (543)*	*Enterobacterales (91347)*	Gammaproteobacteria (1236)	Proteobacteria (1224)	-2.5569785	3.3618129999999997
