# Supplementary material for: Colistin Dependence in Extensively Drug-Resistant Acinetobacter baumannii Strain Is Associated with ISAjo2 and ISAba13 Insertions and Multiple Cellular Responses
Source: Int J Mol Sci. 2021 Jan 8;22(2):576. doi: 10.3390/ijms22020576 (PMC7827689; doi:10.3390/ijms22020576)
Supplement: Supplementary file 1 [file ijms-22-00576-s001.zip › Table S4.DOCX]

| **Table S4.** Proteins upregulated in colistin-dependent subpopulation in comparison to colistin susceptible | | | |
| --- | --- | --- | --- |
| Protein name | NCBI accession number | Fold change | p-value |
| hypothetical protein | WP_002046159.1 | INF | 0.0041 |
| NAD(P)/FAD-dependent oxidoreductase | WP_057691010.1 | INF | 0.00036 |
| conserved hypothetical protein; putative exported protein | CAM86312.1 | INF | 0.00033 |
| hypothetical protein | WP_000917295.1 | INF | < 0.00010 |
| MacA family efflux pump subunit | WP_001124213.1 | INF | < 0.00010 |
| conserved hypothetical protein; putative exported protein | CAM86313.1 | 38 | < 0.00010 |
| hypothetical protein | WP_000738603.1 | 20 | < 0.00010 |
| conserved hypothetical protein; putative signal peptide | CAM85333.1 | 14 | 0.00042 |
| tRNA (N6-isopentenyl adenosine(37)-C2)-methylthiotransferase MiaB | WP_000218141.1 | 12 | 0.00061 |
| type VI secretion system tip protein VgrG | WP_161283681.1 | 12 | 0.0055 |
| putative aspartate ammonia-lyase | ELW83348.1 | 11 | < 0.00010 |
| succinate CoA transferase | WP_000059344.1 | 11 | < 0.00010 |
| ATP-dependent RNA helicase | BAN86335.1 | 10 | 0.0013 |
| hypothetical protein HMPREF0010_01233 | EEX03839.1 | 8.4 | < 0.00010 |
| type VI secretion system tip protein VgrG | WP_000935013.1 | 8.3 | 0.00025 |
| integrase | WP_057690988.1 | 8.2 | 0.0016 |
| dicarboxylate/amino acid:cation symporter | WP_000347180.1 | 7.4 | 0.00023 |
| type VI secretion system tip protein VgrG | WP_057691008.1 | 6.3 | 0.0008 |
| type VI secretion system tube protein Hcp | WP_000653195.1 | 5.9 | 0.0035 |
| phosphomethylpyrimidine synthase ThiC | WP_071217137.1 | 5.2 | 0.0004 |
| rhodanese domain-containing protein | WP_000994655.1 | 5.2 | 0.004 |
| type IV secretion protein Rhs | WP_000081475.1 | 4.9 | < 0.00010 |
| hypothetical protein | WP_001009738.1 | 4.7 | < 0.00010 |
| Acetate kinase | CQR66899.1 | 4.7 | 0.00034 |
| AdeC/AdeK/OprM family multidrug efflux complex outer membrane factor | WP_000010517.1 | 4.3 | 0.0035 |
| carbapenem susceptibility porin CarO | WP_000733010.1 | 3.9 | 0.002 |
| undecaprenyl-diphosphate phosphatase | WP_000426935.1 | 3.9 | 0.0022 |
| pyruvate decarboxylase | WP_000469453.1 | 3.7 | < 0.00010 |
| hypothetical protein | WP_000357153.1 | 3.5 | 0.0025 |
| type IV secretion protein Rhs (ACJ40183.1) | ACJ40183.1 | 3 | 0.00018 |
| putative Isochorismatase hydrolase | CAM86638.1 | 2.9 | 0.00051 |
| ATP-dependent Clp protease ATP-binding subunit ClpX | WP_001289250.1 | 2.9 | < 0.00010 |
| type VI secretion system ATPase TssH | WP_000987834.1 | 2.8 | < 0.00010 |
| AdeB family multidrug efflux RND transporter permease subunit | WP_000046678.1 | 2.7 | < 0.00010 |
| acetyltransferase | WP_000066535.1 | 2.7 | 0.0041 |
| methionine adenosyltransferase | WP_001209545.1 | 2.7 | 0.0014 |
| tRNA uridine-5-carboxymethylaminomethyl(34) synthesis enzyme MnmG | WP_000559187.1 | 2.7 | 0.00053 |
| 2-isopropylmalate synthase | BAN88908.1 | 2.6 | 0.0017 |
| hypothetical protein | WP_000784270.1 | 2.5 | 0.0021 |
| Dihydropteroate synthase | AFB76381.1 | 2.5 | 0.0042 |
| NAD-dependent epimerase/dehydratase family protein | WP_000697856.1 | 2.4 | 0.0014 |
| putative signal peptide peptidase SppA [Acinetobacter baumannii 6013150] | EGJ58389.1 | 2.4 | 0.0022 |
| catalase peroxidase | WP_000064284.1 | 2.3 | 0.00028 |
| methionine synthase | WP_000735356.1 | 2.3 | < 0.00010 |
| sorbosone dehydrogenase family protein | WP_000849751.1 | 2.3 | 0.0016 |
| protein translocase subunit SecF | WP_001985897.1 | 2.3 | 0.0036 |
| AdeA/AdeI family multidrug efflux RND transporter periplasmic adaptor subunit | WP_000986589.1 | 2.1 | < 0.00010 |
| DUF1508 domain-containing protein | WP_000929502.1 | 2 | 0.0024 |
| multidrug efflux RND transporter permease subunit AdeB | WP_000987602.1 | 2 | 0.0011 |
| multidrug efflux RND transporter AdeIJK outer membrane channel subunit AdeK | WP_001174793.1 | 2 | 0.0021 |
| ribonucleoside-diphosphate reductase. beta subunit | EGJ60866.1 | 1.9 | 0.00018 |
| multidrug efflux RND transporter periplasmic adaptor subunit AdeA | WP_001169096.1 | 1.9 | 0.0026 |
| Glycine/D-amino acid oxidase (deaminating) | ACC55435.1 | 1.9 | 0.0017 |
| polysaccharide biosynthesis protein | WP_000721143.1 | 1.9 | < 0.00010 |
| polysaccharide pyruvyl transferase family protein | WP_000877637.1 | 1.9 | 0.0043 |
| trifunctional transcriptional regulator/proline dehydrogenase/L-glutamate gamma-semialdehyde dehydrogenase | WP_103236474.1 | 1.7 | < 0.00010 |
| aromatic amino acid aminotransferase | BAN89270.1 | 1.7 | 0.00066 |
| RNA-binding transcriptional accessory protein | WP_000131198.1 | 1.7 | 0.0003 |
| Lon protease | AEP05596.2 | 1.6 | 0.00027 |
| UDP-N-acetylglucosamine diphosphorylase/glucosamine-1-phosphate N-acetyltransferase | WP_057052289.1 | 1.6 | 0.00077 |
| leucine--tRNA ligase | WP_000155773.1 | 1.6 | < 0.00010 |
| aspartate kinase | WP_001185176.1 | 1.6 | 0.0016 |
| glutamine--fructose-6-phosphate transaminase (isomerizing) | WP_000334179.1 | 1.5 | < 0.00010 |
| aldehyde dehydrogenase (NAD) family protein | EFF85880.1 | 1.4 | 0.0035 |
| ribonucleoside-diphosphate reductase subunit alpha | WP_000111540.1 | 1.4 | 0.0007 |
| 4-hydroxyphenylpyruvate dioxygenase | WP_000353163.1 | 1.4 | 0.0041 |
| aconitate hydratase 2 | EGJ61840.1 | 1.4 | 0.00017 |
