# Supplementary material for: Colistin Dependence in Extensively Drug-Resistant Acinetobacter baumannii Strain Is Associated with ISAjo2 and ISAba13 Insertions and Multiple Cellular Responses
Source: Int J Mol Sci. 2021 Jan 8;22(2):576. doi: 10.3390/ijms22020576 (PMC7827689; doi:10.3390/ijms22020576)
Supplement: Supplementary file 1 [file ijms-22-00576-s001.zip › Table S5.DOCX]

| **Table S5**. Proteins downregulated in colistin-dependent subpopulation in comparison to colistin susceptible | | | |
| --- | --- | --- | --- |
| **Protein name** | **NCBI accesion number** | **Fold change** | **p-value** |
| tryptophan synthase subunit alpha | WP_000088559.1 | 0.8 | 0.0058 |
| two-component system response regulator OmpR | WP_000060753.1 | 0.7 | 0.00011 |
| pyridoxal phosphate-dependent aminotransferase | WP_000383202.1 | 0.7 | < 0.00010 |
| blaOXA-23 (OXA-23 carbapenemase) | VCZ51052.1 | 0.7 | 0.00053 |
| long-chain-fatty-acid--CoA ligase | WP_000411991.1 | 0.7 | 0.00048 |
| NADH-dependent enoyl-ACP reductase | SSP50919.1 | 0.7 | 0.0045 |
| TonB-dependent siderophore receptor | WP_000364482.1 | 0.7 | 0.00095 |
| 3-hydroxybutyrate dehydrogenase | WP_000163854.1 | 0.6 | 0.0035 |
| peptide deformylase | WP_001196539.1 | 0.6 | 0.0013 |
| fabD  (acyl-carrier-protein) S-malonyltransferase | ADX02448.1 | 0.6 | 0.0023 |
| LPS-assembly protein LptD | WP_045544211.1 | 0.6 | 0.0046 |
| beta-ketoacyl-ACP synthase II | WP_000832710.1 | 0.6 | 0.0024 |
| BON domain-containing protein | WP_000919363.1 | 0.6 | 0.00078 |
| hypothetical protein AB237_0320 | BAN86292.1 | 0.6 | 0.0016 |
| FAD-binding protein | WP_000584426.1 | 0.6 | 0.00033 |
| ferric uptake regulation protein | BAN88541.1 | 0.6 | 0.0033 |
| alkyl hydroperoxide reductase subunit F | WP_000886809.1 | 0.6 | < 0.00010 |
| type III secretion system protein EcsC | WP_032038602.1 | 0.5 | 0.00019 |
| putative hydro-lyase | WP_065717966.1 | 0.5 | 0.0023 |
| Adhesin/BapA prefix-like domain-containing protein | KRJ95188.1/WP_000196831.1 | 0.5 | 0.00023 |
| TonB-dependent receptor | WP_000413997.1 | 0.5 | < 0.00010 |
| outer membrane lipoprotein chaperone LolA | WP_001056757.1 | 0.5 | 0.00024 |
| IscS subfamily cysteine desulfurase | WP_000828390.1 | 0.5 | 0.0026 |
| superoxide dismutase precursor (Cu-Zn) | CAM85318.1 | 0.5 | < 0.00010 |
| acetyl-CoA C-acyltransferase FadA | WP_000212712.1 | 0.5 | 0.0057 |
| putative serine protease | CAM85881.1 | 0.5 | < 0.00010 |
| LysM peptidoglycan-binding domain-containing protein | WP_000550750.1 | 0.5 | 0.00036 |
| outer membrane protein assembly factor/  outer membrane protein. OMP85 family | WP_001139432.1/EGJ61843.1 | 0.5 | 0.00036 |
| hypothetical protein | WP_000251635.1 | 0.5 | 0.0014 |
| glutamate synthase subunit beta | WP_000840834.1 | 0.5 | 0.0026 |
| putative TonB-dependent Outer membrane receptor for vitamin B12/cobalamin transport (Btub) | CAM85573.1 | 0.5 | < 0.00010 |
| enoyl-CoA hydratase | WP_001198691.1 | 0.4 | 0.0042 |
| 3-hydroxyacyl-CoA dehydrogenase | WP_000701535.1 | 0.4 | 0.00034 |
| AdeT. RND type efflux pump | ADX01640.1 | 0.4 | 0.00021 |
| hydroxymethylglutaryl-CoA lyase | WP_001288910.1 | 0.4 | 0.00031 |
| outer membrane lipoprotein Blc | BAN86810.1 | 0.4 | 0.00012 |
| peroxiredoxin | WP_000175332.1 | 0.4 | < 0.00010 |
| alpha/beta hydrolase | WP_000830596.1 | 0.4 | < 0.00010 |
| hypothetical protein | WP_001178068.1 | 0.4 | 0.00015 |
| VacJ family lipoprotein | WP_001109851.1 | 0.4 | 0.0026 |
| KR domain-containing protein | WP_001035486.1 | 0.4 | < 0.00010 |
| beta-lactamase | AEP07218.1 | 0.4 | < 0.00010 |
| ErfK/YbiS/YcfS/YnhG (murein L.D-transpeptidase) | EEX03271.1 /WP_000498093.1 | 0.4 | 0.00033 |
| putative lipopolysaccharide transport protein A (ABC superfamily. peri_bind) (LptA) | CAM87332.1 | 0.4 | 0.0049 |
| acyl-CoA dehydrogenase | WP_000576648.1 | 0.4 | 0.0054 |
| DUF3108 domain-containing protein | WP_000201547.1 | 0.4 | < 0.00010 |
| hypothetical protein | WP_000161254.1 | 0.4 | 0.00018 |
| methylmalonate-semialdehyde dehydrogenase. oxidoreductase protein | CAM88529.1 | 0.4 | < 0.00010 |
| catechol 1.2-dioxygenase | WP_001081618.1 | 0.4 | < 0.00010 |
| hypothetical protein | WP_000701849.1 | 0.4 | 0.00013 |
| type II asparaginase | WP_000956445.1 | 0.3 | < 0.00010 |
| putative RND family drug transporter | CAJ77853.1 | 0.3 | 0.00013 |
| nitroreductase | WP_002046928.1 | 0.3 | 0.0013 |
| erythronate-4-phosphate dehydrogenase | WP_000706080.1 | 0.3 | 0.0023 |
| porin oprB | WP_000848134.1 | 0.3 | < 0.00010 |
| amino acid ABC transporter substrate-binding protein | WP_052137106.1 | 0.3 | < 0.00010 |
| quinoprotein glucose dehydrogenase | WP_001032848.1 | 0.3 | < 0.00010 |
| MBL fold metallo-hydrolase | WP_000732912.1 | 0.3 | < 0.00010 |
| DUF1442 domain-containing protein | WP_001133993.1 | 0.3 | < 0.00010 |
| Fe-S cluster assembly scaffold IscU | WP_057690997.1 | 0.3 | < 0.00010 |
| polyisoprenoid-binding protein | WP_000550334.1 | 0.3 | < 0.00010 |
| tail-specific protease | WP_050676158.1 | 0.3 | < 0.00010 |
| DUF2171 domain-containing protein | WP_001094391.1 | 0.3 | < 0.00010 |
| copper chaperone | WP_078210158.1 | 0.3 | 0.00014 |
| glycine cleavage system protein GcvH | WP_001016343.1 | 0.3 | 0.00026 |
| toluene tolerance protein Ttg2A/ ABC transporter ATP-binding protein | EEX02911.1/ WP_002135589.1 | 0.3 | 0.0059 |
| hypothetical protein | WP_000721434.1 | 0.2 | 0.0022 |
| conserved hypothetical protein; putative exported protein | CAM85913.1 | 0.2 | < 0.00010 |
| Quinoprotein glucose dehydrogenase-B precursor | CAM86640.1 | 0.2 | < 0.00010 |
| hypothetical protein | WP_000983536.1 | 0.2 | 0.0047 |
| type 1 glutamine amidotransferase domain-containing protein | WP_000044208.1 | 0.2 | 0.0006 |
| membrane protein | WP_000180179.1 | 0.2 | 0.00012 |
| fatty acyl-CoA reductase (hexadecanal dehydrogenase.acylating) | CAO99653.1 | 0.2 | < 0.00010 |
| catalase | WP_001061472.1 | 0.2 | 0.0057 |
| butyryl-CoA dehydrogenase | WP_000602467.1 | 0.2 | 0.00021 |
| hypothetical protein | WP_001046928.1 | 0.2 | < 0.00010 |
| FklB | AIT56340.1 | 0.2 | 0.00013 |
| lytic murein transglycosylase (MltB-like) | WP_001252448.1 | 0.2 | < 0.00010 |
| lactonase | ERH68036.1 | 0.2 | 0.004 |
| CYTH and CHAD domain-containing protein | WP_000230489.1 | 0.2 | 0.0021 |
| glutamate synthase subunit alpha | BAN86260.1 | 0.2 | 0.0048 |
| hypothetical protein | WP_000720299.1 | 0.2 | 0.0049 |
| hypothetical protein | WP_000846679.1 | 0.2 | < 0.00010 |
| hypothetical protein | WP_000719146.1 | 0.2 | 0.00013 |
| 3-hydroxyisobutyrate dehydrogenase | WP_001017291.1 | 0.2 | < 0.00010 |
| conserved hypothetical protein; putative metal-dependent hydrolase | CAM87067.1 | 0.2 | 0.0034 |
| lytic murein transglycosylase B (MltB-like) | WP_000951088.1 | 0.2 | < 0.00010 |
| putative acyl-CoA dehydrogenase-related protein | CAM86218.1 | 0.2 | 0.0006 |
| galactose-1-epimerase | WP_000731756.1 | 0.2 | < 0.00010 |
| conserved hypothetical protein; putative exported protein | CAM88269.1 | 0.2 | < 0.00010 |
| CoA transferase subunit B | WP_000121760.1 | 0.1 | 0.0018 |
| hypothetical protein | WP_000132046.1 | 0.1 | < 0.00010 |
| acyl-CoA dehydrogenase | WP_001160766.1 | 0.1 | < 0.00010 |
| phosphate ABC transporter. phosphate-binding protein | EGJ58385.1 | 0.1 | < 0.00010 |
| conserved hypothetical protein; putative outer membrane protein | CAM86400.1 | 0.1 | < 0.00010 |
| GNAT family N-acetyltransferase | WP_000637089.1 | 0.1 | 0.0046 |
| TIGR04219 family outer membrane beta-barrel protein | WP_001256691.1 | 0.1 | < 0.00010 |
| PspC domain-containing protein | WP_000013717.1 | 0.1 | < 0.00010 |
| autotransporter domain-containing protein | WP_001260880.1 | 0.1 | 0.00025 |
| rhombotarget A | WP_000920020.1 | 0.1 | 0.0012 |
| NirD/YgiW/YdeI family stress tolerance protein | WP_000721827.1 | 0.1 | 0.0016 |
| YARHG domain-containing protein | WP_001037927.1 | 0.1 | 0.00046 |
| NAD(P)-dependent oxidoreductase | WP_000432325.1 | 0.1 | < 0.00010 |
| stress-induced protein | WP_000024222.1 | 0.09 | < 0.00010 |
| aldehyde reductase | CAM85028.1 | 0.09 | 0.00055 |
| DUF4142 domain-containing protein | WP_000644336.1 | 0.09 | < 0.00010 |
| AMP-binding protein | WP_000969576.1 | 0.08 | < 0.00010 |
| DsbC family protein | WP_000850475.1 | 0.08 | 0.00033 |
| trehalose-6-phosphate phophatase. biosynthetic | CAM87826.1 | 0.07 | 0.0013 |
| Zn-dependent oligopeptidase | WP_000719397.1 | 0.07 | 0.0025 |
| serine hydrolase family protein | WP_002018090.1 | 0.07 | 0.00024 |
| conserved hypothetical protein | CAP00957.1 | 0.07 | < 0.00010 |
| hypothetical protein | WP_002000780.1 | 0.07 | < 0.00010 |
| glutathione-dependent formaldehyde dehydrogenase | WP_001202393.1 | 0.06 | < 0.00010 |
| transglycosylase SLT domain protein | EGJ58788.1 | 0.05 | 0.0028 |
| phospholipase A1 (pldA) | EGJ62971.1 | 0.04 | < 0.00010 |
| putative PQQ-dependent aldose sugar dehydrogenase precursor | CAM85041.1 | 0.04 | < 0.00010 |
| DUF1311 domain-containing protein | WP_000724608.1 | 0.02 | < 0.00010 |
| hypothetical protein | WP_000247196.1 | 0.01 | < 0.00010 |
| Putative RND family drug transporter (outer membrane efflux protein) | CAJ77861.1 | 0 | 0.006 |
| class I SAM-dependent methyltransferase | WP_000581927.1 | 0 | 0.003 |
| head morphogenesis protein | WP_000790401.1 | 0 | 0.0019 |
| molybdate ABC transporter substrate-binding protein | WP_000253153.1 | 0 | 0.0018 |
| amidohydrolase | WP_032061616.1 | 0 | 0.00089 |
| acyl-[acyl-carrier-protein]--UDP-N-acetylglucosamine O-acyltransferase. partial **^*^** | OFD23753.1 | 0 | 0.00018 |
| DUF4882 domain-containing protein | WP_001991976.1 | 0 | < 0.00010 |
| hypothetical protein AB237_0628 | BAN86579.1 | 0 | < 0.00010 |
| acyl-ACP--UDP-N-acetylglucosamine O-acyltransferase **^**^** | WP_ 031976200.1 | 0 | < 0.00010 |
| outer membrane lipid asymmetry maintenance protein MlaD | WP_098732046.1 | 0 | < 0.00010 |
| ^*^ N-terminal part of LpxA  ^**^ C-terminal part of LpxA | | | |
