# Supplementary material for: Colistin Dependence in Extensively Drug-Resistant Acinetobacter baumannii Strain Is Associated with ISAjo2 and ISAba13 Insertions and Multiple Cellular Responses
Source: Int J Mol Sci. 2021 Jan 8;22(2):576. doi: 10.3390/ijms22020576 (PMC7827689; doi:10.3390/ijms22020576)
Supplement: Supplementary file 1 [file Table S3.pdf › Table S3.pdf]

Database: ISfinder_Nucl
5,559 sequences; 8,208,285 total letters

Query= MT-1_S8_L001_R1_001_(paired)_trimmed_(paired)_contig_1
Length=308325

Sequences producing significant alignments	IS Family	Group	Origin	Score (bits)	E. value
ISAjo2	ISNCY	IS1202	Acinetobacter johnsonii	131	7E-28
ISAba1	IS4	IS10	Acinetobacter baumannii	131	7E-28
ISAba33	IS4	IS10	Acinetobacter baumannii	69.9	0,000000002

Query= MT-1_S8_L001_R1_001_(paired)_trimmed_(paired)_contig_2
Length=289160

Sequences producing significant alignments	IS Family	Group	Origin	Score (bits)	E. value
ISAba13	IS5	IS903	Acinetobacter baumannii	119	3E-24
ISPpu12	ISL3		Pseudomonas putida	119	3E-24
IS1396	ISL3		Serratia marcescens	119	3E-24
ISPst2	ISL3		Pseudomonas stutzeri	85.7	4E-14
ISPst9	ISL3		Pseudomonas stutzeri	63.9	0,0000001

Query= MT-1_S8_L001_R1_001_(paired)_trimmed_(paired)_contig_3
Length=237801

Sequences producing significant alignments	IS Family	Group	Origin	Score (bits)	E. value
ISAba1	IS4	IS10	Acinetobacter baumannii	180	7E-43
ISAba33	IS4	IS10	Acinetobacter baumannii	111	5E-22

Query= MT-1_S8_L001_R1_001_(paired)_trimmed_(paired)_contig_4
Length=202703

Sequences producing significant alignments	IS Family	Group	Origin	Score (bits)	E. value
ISAba1	IS4	IS10	Acinetobacter baumannii	180	6E-43
ISAba13	IS5	IS903	Acinetobacter baumannii	119	2E-24
ISAba33	IS4	IS10	Acinetobacter baumannii	111	4E-22
ISAha1	IS5	IS903	Acinetobacter haemolyticus	111	4E-22
ISAba53	IS5	IS903	Acinetobacter baumannii	103	1E-19
IS17	IS5	IS903	Acinetobacter haemolyticus	77.8	6E-12
ISAba12	IS5	IS903	Acinetobacter baumannii	69.9	0,000000002
ISAba40	IS5	IS903	Acinetobacter baumannii	56.0	0,00002
ISAjo1	IS5	IS903	Acinetobacter johnsonii	54.0	0,00009
ISAba5	IS5	IS903	Acinetobacter baumannii	54.0	0,00009

Query= MT-1_S8_L001_R1_001_(paired)_trimmed_(paired)_contig_6
Length=170838

Sequences producing significant alignments	IS Family	Group	Origin	Score (bits)	E. value
ISAba1	IS4	IS10	Acinetobacter baumannii	131	4E-28
ISAba33	IS4	IS10	Acinetobacter baumannii	69.9	0,000000001

Query= MT-1_S8_L001_R1_001_(paired)_trimmed_(paired)_contig_7
Length=155325

Sequences producing significant alignments	IS Family	Group	Origin	Score (bits)	E. value
ISAba1	IS4	IS10	Acinetobacter baumannii	180	4E-43
ISAba33	IS4	IS10	Acinetobacter baumannii	111	3E-22

Query= MT-1_S8_L001_R1_001_(paired)_trimmed_(paired)_contig_8
Length=153568

Sequences producing significant alignments	IS Family	Group	Origin	Score (bits)	E. value
ISAjo2	ISNCY	IS1202	Acinetobacter johnsonii	131	4E-28
ISAba13	IS5	IS903	Acinetobacter baumannii	119	1E-24
ISAlw34	IS66		Acinetobacter lwoffii	58.0	0,000004
ISAlw16	IS66		Acinetobacter lwoffii	58.0	0,000004
ISAba16	IS66		Acinetobacter baumannii	58.0	0,000004
ISAba17	IS66		Acinetobacter baumannii	58.0	0,000004

Query= MT-1_S8_L001_R1_001_(paired)_trimmed_(paired)_contig_9
Length=148232

Sequences producing significant alignments	IS Family	Group	Origin	Score (bits)	E. value
ISAba1	IS4	IS10	Acinetobacter baumannii	180	4E-43
ISAba13	IS5	IS903	Acinetobacter baumannii	119	1E-24
ISAba33	IS4	IS10	Acinetobacter baumannii	111	3E-22
ISAha1	IS5	IS903	Acinetobacter haemolyticus	111	3E-22
ISAba53	IS5	IS903	Acinetobacter baumannii	103	8E-20
IS17	IS5	IS903	Acinetobacter haemolyticus	77.8	5E-12
ISAba12	IS5	IS903	Acinetobacter baumannii	69.9	0,000000001
ISAba40	IS5	IS903	Acinetobacter baumannii	56.0	0,00002
ISAjo1	IS5	IS903	Acinetobacter johnsonii	54.0	0,00007

ISAba5	*IS5*	*IS903*	*Acinetobacter baumannii*	54.0	0,00007

Query= MT-1_S8_L001_R1_001_(paired)_trimmed_(paired)_contig_10
Length=148131

Sequences producing significant alignments	*IS Family*	*Group*	*Origin*	*Score (bits)*	*E. value*
ISAjo2	*ISNCY*	*IS1202*	*Acinetobacter johnsonii*	131	4E-28

Query= MT-1_S8_L001_R1_001_(paired)_trimmed_(paired)_contig_11
Length=140828

Sequences producing significant alignments	*IS Family*	*Group*	*Origin*	*Score (bits)*	*E. value*
ISAba1	*IS4*	*IS10*	*Acinetobacter baumannii*	180	4E-43
ISAjo2	*ISNCY*	*IS1202*	*Acinetobacter johnsonii*	131	3E-28
ISAba33	*IS4*	*IS10*	*Acinetobacter baumannii*	111	3E-22

Query= MT-1_S8_L001_R1_001_(paired)_trimmed_(paired)_contig_12
Length=138277

Sequences producing significant alignments	*IS Family*	*Group*	*Origin*	*Score (bits)*	*E. value*
ISAba1	*IS4*	*IS10*	*Acinetobacter baumannii*	180	4E-43
ISAba33	*IS4*	*IS10*	*Acinetobacter baumannii*	111	3E-22

Query= MT-1_S8_L001_R1_001_(paired)_trimmed_(paired)_contig_13
Length=125408

Sequences producing significant alignments	*IS Family*	*Group*	*Origin*	*Score (bits)*	*E. value*
ISAba1	*IS4*	*IS10*	*Acinetobacter baumannii*	180	4E-43
ISAba33	*IS4*	*IS10*	*Acinetobacter baumannii*	111	3E-22

Query= MT-1_S8_L001_R1_001_(paired)_trimmed_(paired)_contig_14
Length=120407

Sequences producing significant alignments	*IS Family*	*Group*	*Origin*	*Score (bits)*	*E. value*
ISAba13	*IS5*	*IS903*	*Acinetobacter baumannii*	2060	0.0
ISAba12	*IS5*	*IS903*	*Acinetobacter baumannii*	848	0.0
IS17	*IS5*	*IS903*	*Acinetobacter haemolyticus*	835	0.0
ISAba40	*IS5*	*IS903*	*Acinetobacter baumannii*	803	0.0
ISAha1	*IS5*	*IS903*	*Acinetobacter haemolyticus*	801	0.0
ISAlw1	*IS5*	*IS903*	*Acinetobacter lwoffii*	704	0.0
ISAjo1	*IS5*	*IS903*	*Acinetobacter johnsonii*	636	2E-180
ISAba53	*IS5*	*IS903*	*Acinetobacter baumannii*	617	2E-174
ISAha3	*IS5*	*IS903*	*Acinetobacter haemolyticus*	517	1E-144
ISAha2	*IS5*	*IS903*	*Acinetobacter haemolyticus*	428	9E-118
ISAba7	*IS5*	*IS903*	*Acinetobacter baumannii*	420	2E-115
ISAba5	*IS5*	*IS903*	*Acinetobacter baumannii*	387	3E-105

Query= MT-1_S8_L001_R1_001_(paired)_trimmed_(paired)_contig_15
Length=120157

Sequences producing significant alignments	*IS Family*	*Group*	*Origin*	*Score (bits)*	*E. value*
ISAba1	*IS4*	*IS10*	*Acinetobacter baumannii*	131	3E-28
ISAba33	*IS4*	*IS10*	*Acinetobacter baumannii*	69.9	9E-10

Query= MT-1_S8_L001_R1_001_(paired)_trimmed_(paired)_contig_16
Length=117829

Sequences producing significant alignments	*IS Family*	*Group*	*Origin*	*Score (bits)*	*E. value*
ISAjo2	*ISNCY*	*IS1202*	*Acinetobacter johnsonii*	119	1E-24

Query= MT-1_S8_L001_R1_001_(paired)_trimmed_(paired)_contig_17
Length=105540

Sequences producing significant alignments	*IS Family*	*Group*	*Origin*	*Score (bits)*	*E. value*
ISAba1	*IS4*	*IS10*	*Acinetobacter baumannii*	180	3E-43
ISAba33	*IS4*	*IS10*	*Acinetobacter baumannii*	111	2E-22

Query= MT-1_S8_L001_R1_001_(paired)_trimmed_(paired)_contig_18
Length=87980

Sequences producing significant alignments	*IS Family*	*Group*	*Origin*	*Score (bits)*	*E. value*
ISAba26	*IS256*		*Acinetobacter baumannii*	2605	0.0
ISAba13	*IS5*	*IS903*	*Acinetobacter baumannii*	119	8E-25
ISAbe10	*IS256*		*Acinetobacter bereziniae*	111	2E-22
ISAba42	*IS256*		*Acinetobacter baumannii*	83.8	4E-14
ISAba50	*IS256*		*Acinetobacter baumannii*	56.0	0,00001

Query= MT-1_S8_L001_R1_001_(paired)_trimmed_(paired)_contig_19
Length=75938

Sequences producing significant alignments	IS Family	Group	Origin	Score (bits)	E. value
ISAba13	*IS5*	*IS903*	*Acinetobacter baumannii*	119	7E-25
ISAha1	*IS5*	*IS903*	*Acinetobacter haemolyticus*	111	2E-22
ISAba53	*IS5*	*IS903*	*Acinetobacter baumannii*	103	4E-20
IS17	*IS5*	*IS903*	*Acinetobacter haemolyticus*	77.8	2E-12
ISAba12	*IS5*	*IS903*	*Acinetobacter baumannii*	69.9	6E-10
ISAba40	*IS5*	*IS903*	*Acinetobacter baumannii*	56.0	0,000009
ISAjo1	*IS5*	*IS903*	*Acinetobacter johnsonii*	54.0	0,00003
ISAba5	*IS5*	*IS903*	*Acinetobacter baumannii*	54.0	0,00003

Query= MT-1_S8_L001_R1_001_(paired)_trimmed_(paired)_contig_20
Length=73065

Sequences producing significant alignments	IS Family	Group	Origin	Score (bits)	E. value
ISAba1	*IS4*	*IS10*	*Acinetobacter baumannii*	131	2E-28
ISAba33	*IS4*	*IS10*	*Acinetobacter baumannii*	69.9	5E-10

Query= MT-1_S8_L001_R1_001_(paired)_trimmed_(paired)_contig_21
Length=67943

Sequences producing significant alignments	IS Family	Group	Origin	Score (bits)	E. value
ISAba13	*IS5*	*IS903*	*Acinetobacter baumannii*	119	6E-25
ISAha1	*IS5*	*IS903*	*Acinetobacter haemolyticus*	111	1E-22
ISAba53	*IS5*	*IS903*	*Acinetobacter baumannii*	103	4E-20
IS17	*IS5*	*IS903*	*Acinetobacter haemolyticus*	77.8	2E-12
ISAba12	*IS5*	*IS903*	*Acinetobacter baumannii*	69.9	5E-10
ISAba40	*IS5*	*IS903*	*Acinetobacter baumannii*	56.0	0,000008
ISAjo1	*IS5*	*IS903*	*Acinetobacter johnsonii*	54.0	0,00003
ISAba5	*IS5*	*IS903*	*Acinetobacter baumannii*	54.0	0,00003

Query= MT-1_S8_L001_R1_001_(paired)_trimmed_(paired)_contig_22
Length=66290

Sequences producing significant alignments	IS Family	Group	Origin	Score (bits)	E. value
ISAba1	*IS4*	*IS10*	*Acinetobacter baumannii*	131	2E-28
ISAba33	*IS4*	*IS10*	*Acinetobacter baumannii*	69.9	5E-10

Query= MT-1_S8_L001_R1_001_(paired)_trimmed_(paired)_contig_23
Length=61070

Sequences producing significant alignments	IS Family	Group	Origin	Score (bits)	E. value
ISPpu12	*ISL3*		*Pseudomonas putida*	119	6E-25
IS1396	*ISL3*		*Serratia marcescens*	119	6E-25
ISPst2	*ISL3*		*Pseudomonas stutzeri*	85.7	8E-15
ISPst9	*ISL3*		*Pseudomonas stutzeri*	63.9	0,00000003

Query= MT-1_S8_L001_R1_001_(paired)_trimmed_(paired)_contig_24
Length=55919

Sequences producing significant alignments	IS Family	Group	Origin	Score (bits)	E. value
ISAba1	*IS4*	*IS10*	*Acinetobacter baumannii*	131	1E-28
ISAba33	*IS4*	*IS10*	*Acinetobacter baumannii*	69.9	4E-10

Query= MT-1_S8_L001_R1_001_(paired)_trimmed_(paired)_contig_25
Length=53672

Sequences producing significant alignments	IS Family	Group	Origin	Score (bits)	E. value
ISAba1	*IS4*	*IS10*	*Acinetobacter baumannii*	131	1E-28
ISAba33	*IS4*	*IS10*	*Acinetobacter baumannii*	69.9	4E-10

Query= MT-1_S8_L001_R1_001_(paired)_trimmed_(paired)_contig_26
Length=50216

Sequences producing significant alignments	IS Family	Group	Origin	Score (bits)	E. value
ISAba125	*IS30*		*Acinetobacter baumannii*	119	5E-25
ISC1041	*IS30*		*Sulfolobus solfataricus*	61.9	0,00000009

Query= MT-1_S8_L001_R1_001_(paired)_trimmed_(paired)_contig_27
Length=50075

Sequences producing significant alignments	IS Family	Group	Origin	Score (bits)	E. value
ISAba1	*IS4*	*IS10*	*Acinetobacter baumannii*	131	1E-28
ISAba33	*IS4*	*IS10*	*Acinetobacter baumannii*	69.9	4E-10

Query= MT-1_S8_L001_R1_001_(paired)_trimmed_(paired)_contig_29
Length=40833

Sequences producing significant alignments	IS Family	Group	Origin	Score (bits)	E. value
ISAba1	*IS4*	*IS10*	*Acinetobacter baumannii*	180	1E-43
ISAba13	*IS5*	*IS903*	*Acinetobacter baumannii*	119	4E-25

ISAba33	IS4	IS10	*Acinetobacter baumannii*	111	9E-23
ISAjo1	IS5	IS903	*Acinetobacter johnsonii*	52.0	0,00007
ISAha2	IS5	IS903	*Acinetobacter haemolyticus*	52.0	0,00007

Query= MT-1_S8_L001_R1_001_(paired)_trimmed_(paired)_contig_31
Length=36859

Sequences producing significant alignments	IS Family	Group	Origin	Score (bits)	E. value
ISAba1	IS4	IS10	*Acinetobacter baumannii*	131	9E-29
ISAba13	IS5	IS903	*Acinetobacter baumannii*	119	3E-25
ISAha1	IS5	IS903	*Acinetobacter haemolyticus*	111	8E-23
ISAba53	IS5	IS903	*Acinetobacter baumannii*	103	2E-20
IS17	IS5	IS903	*Acinetobacter haemolyticus*	77.8	1E-12
ISAba33	IS4	IS10	*Acinetobacter baumannii*	69.9	3E-10
ISAba12	IS5	IS903	*Acinetobacter baumannii*	69.9	3E-10
ISAba40	IS5	IS903	*Acinetobacter baumannii*	56.0	0,000004
ISAjo1	IS5	IS903	*Acinetobacter johnsonii*	54.0	0,00002
ISAba5	IS5	IS903	*Acinetobacter baumannii*	54.0	0,00002

Query= MT-1_S8_L001_R1_001_(paired)_trimmed_(paired)_contig_32
Length=34444

Sequences producing significant alignments	IS Family	Group	Origin	Score (bits)	E. value
ISAjo2	ISNCY	IS1202	*Acinetobacter johnsonii*	119	3E-25

Query= MT-1_S8_L001_R1_001_(paired)_trimmed_(paired)_contig_34
Length=30872

Sequences producing significant alignments	IS Family	Group	Origin	Score (bits)	E. value
ISAlw4	IS3	IS51	*Acinetobacter lwoffii*	2044	0.0
ISAlw5	IS3	IS51	*Acinetobacter lwoffii*	1941	0.0
ISAlw23	IS3	IS51	*Acinetobacter lwoffii*	1885	0.0
ISAba2	IS3	IS51	*Acinetobacter baumannii*	1721	0.0
ISAba29	IS3	IS51	*Acinetobacter baumannii*	1552	0.0
ISAba34	IS3	IS51	*Acinetobacter baumannii*	959	0.0
ISAba19	IS3	IS51	*Acinetobacter baumannii*	910	0.0
ISAba18	IS3	IS51	*Acinetobacter baumannii*	739	0.0
ISAba27	IS5	ISL2	*Acinetobacter baumannii*	345	3E-93
ISAlw1	IS5	IS903	*Acinetobacter lwoffii*	161	8E-38
ISAba13	IS5	IS903	*Acinetobacter baumannii*	153	2E-35
ISAha1	IS5	IS903	*Acinetobacter haemolyticus*	153	2E-35
ISAjo2	ISNCY	IS1202	*Acinetobacter johnsonii*	131	7E-29
ISAjo1	IS5	IS903	*Acinetobacter johnsonii*	127	1E-27
ISAlw35	IS481		*Acinetobacter lwoffii*	107	1E-21
ISAba40	IS5	IS903	*Acinetobacter baumannii*	107	1E-21
ISAlw24	IS481		*Acinetobacter lwoffii*	99.6	3E-19
ISAba53	IS5	IS903	*Acinetobacter baumannii*	95.6	4E-18
ISSde2	IS481		*Shewanella denitrificans*	83.8	2E-14
ISAba7	IS5	IS903	*Acinetobacter baumannii*	83.8	2E-14
ISAha3	IS5	IS903	*Acinetobacter haemolyticus*	69.9	2E-10
ISAba12	IS5	IS903	*Acinetobacter baumannii*	69.9	2E-10
IS1006	IS6		*Acinetobacter junii*	63.9	0,00000001
ISRtr2	IS3	IS51	*Rhizobium tropici*	58.0	0,0000009
ISAba52	IS6		*Acinetobacter baumannii*	56.0	0,000003
ISPpu33	IS3	IS51	*Pseudomonas putida*	56.0	0,000003
ISStma17	IS3	IS51	*Stenotrophomonas maltophilia*	56.0	0,000003
ISMca3	IS3	IS51	*Methylococcus capsulatus*	56.0	0,000003
ISOur1	IS6		*Oligella urethralis*	56.0	0,000003
IS17	IS5	IS903	*Acinetobacter haemolyticus*	56.0	0,000003
IS1416	IS3	IS51	*Burkholderia glumae*	56.0	0,000003
ISAli4	IS3	IS51	*Azospirillum lipoferum*	54.0	0,00001
ISRme15	IS3	IS51	*Ralstonia metallidurans*	54.0	0,00001
ISPmi1	IS3	IS51	*Proteus mirabilis*	52.0	0,00005

Query= MT-1_S8_L001_R1_001_(paired)_trimmed_(paired)_contig_35
Length=30125

Sequences producing significant alignments	IS Family	Group	Origin	Score (bits)	E. value
ISAba13	IS5	IS903	*Acinetobacter baumannii*	119	3E-25
ISAjo1	IS5	IS903	*Acinetobacter johnsonii*	52.0	0,00005
ISAha2	IS5	IS903	*Acinetobacter haemolyticus*	52.0	0,00005
ISAlw1	IS5	IS903	*Acinetobacter lwoffii*	50.1	0,0002

Query= MT-1_S8_L001_R1_001_(paired)_trimmed_(paired)_contig_37
Length=18461

Sequences producing significant alignments	IS Family	Group	Origin	Score (bits)	E. value
Tn2	Tn3		*Escherichia coli*	8851	0.0
Tn3	Tn3		*Salmonella enterica*	8806	0.0
TnAs1	Tn3		*Aeromonas salmonicida*	2962	0.0
TnAs2	Tn3		*Aeromonas salmonicida*	1838	0.0
ISPa40	Tn3		*Pseudomonas aeruginosa*	1758	0.0
IS6100	IS6		*Mycobacterium fortuitum*	1744	0.0
IS1R	IS1		*Escherichia coli*	1451	0.0

IS1B	*IS1*		*Escherichia coli*	1435	0.0
IS1D	*IS1*		*Escherichia coli*	1419	0.0
IS1G	*IS1*		*Escherichia coli*	1396	0.0
IS1A	*IS1*		*Escherichia coli*	1396	0.0
IS1SD	*IS1*		*Shigella dysenteriae*	1388	0.0
IS1S	*IS1*		*Shigella sonnei*	1372	0.0
TnAs3	*Tn3*		*Aeromonas salmonicida*	1338	0.0
ISShes11	*Tn3*		*Shewanella sp.*	1322	0.0
ISPa38	*Tn3*		*Pseudomonas aeruginosa*	1164	0.0
ISSen9	*IS1*		*Salmonella enterica*	1150	0.0
IS1X2	*IS1*		*Escherichia vulneris*	1102	0.0
IS1X4	*IS1*		*Escherichia hermannii*	991	0.0
IS1X3	*IS1*		*Escherichia fergusonii*	912	0.0
IS1X1	*IS1*		*Shigella flexneri*	864	0.0
IS1F	*IS1*		*Escherichia coli*	864	0.0
ISKpn14	*IS1*		*Klebsiella pneumoniae*	729	0.0
TnShfr1	*Tn3*		*Shewanella frigidimarina*	549	5E-155
ISStma11	*ISL3*		*Stenotrophomonas maltophilia*	492	1E-137
ISEc63	*Tn3*		*Escherichia coli*	486	7E-136
ISSod9	*Tn3*		*Shewanella oneidensis*	371	3E-101
IS15	*IS6*		*Salmonella panama*	121	4E-26
IS15DI	*IS6*		*Salmonella panama*	119	2E-25
IS26	*IS6*		*Proteus vulgaris*	119	2E-25
ISArsp6	*Tn3*		*Arthrobacter sp.*	117	7E-25
IS15DIV	*IS6*		*Salmonella typhimurium*	117	7E-25
IS15DII	*IS6*		*Salmonella panama*	111	4E-23
ISAcsp1	*Tn3*		*Acinetobacter species*	75.8	2E-12
ISStag1	*IS6*		*Stappia aggregata*	71.9	3E-11
Tn5393	*Tn3*		*Erwinia amylovora*	65.9	0,000000002
ISSf1	*IS5*	*IS1031*	*Shigella flexneri*	61.9	0,00000003
ISAcr2	*IS6*		*Acidiphilium cryptum*	54.0	0,000008

Query= MT-1_S8_L001_R1_001_(paired)_trimmed_(paired)_contig_38
Length=17961

Sequences producing significant alignments	IS Family	Group	Origin	Score (bits)	E. value
ISAba13	*IS5*	*IS903*	*Acinetobacter baumannii*	119	2E-25
ISAha1	*IS5*	*IS903*	*Acinetobacter haemolyticus*	111	4E-23
ISAba53	*IS5*	*IS903*	*Acinetobacter baumannii*	103	1E-20
IS17	*IS5*	*IS903*	*Acinetobacter haemolyticus*	77.8	6E-13
ISAba12	*IS5*	*IS903*	*Acinetobacter baumannii*	69.9	1E-10
ISAba40	*IS5*	*IS903*	*Acinetobacter baumannii*	56.0	0,000002
ISAjo1	*IS5*	*IS903*	*Acinetobacter johnsonii*	54.0	0,000008
ISAba5	*IS5*	*IS903*	*Acinetobacter baumannii*	54.0	0,000008
ISAha2	*IS5*	*IS903*	*Acinetobacter haemolyticus*	52.0	0,00003
ISAlw1	*IS5*	*IS903*	*Acinetobacter lwoffii*	50.1	0,0001

Query= MT-1_S8_L001_R1_001_(paired)_trimmed_(paired)_contig_40
Length=16539

Sequences producing significant alignments	IS Family	Group	Origin	Score (bits)	E. value
ISAjo2	*ISNCY*	*IS1202*	*Acinetobacter johnsonii*	119	1E-25
ISAba13	*IS5*	*IS903*	*Acinetobacter baumannii*	119	1E-25
ISAha1	*IS5*	*IS903*	*Acinetobacter haemolyticus*	111	4E-23
ISAba53	*IS5*	*IS903*	*Acinetobacter baumannii*	103	9E-21
IS17	*IS5*	*IS903*	*Acinetobacter haemolyticus*	77.8	5E-13
ISAba12	*IS5*	*IS903*	*Acinetobacter baumannii*	69.9	1E-10
ISAba40	*IS5*	*IS903*	*Acinetobacter baumannii*	56.0	0,000002
ISAjo1	*IS5*	*IS903*	*Acinetobacter johnsonii*	54.0	0,000007
ISAba5	*IS5*	*IS903*	*Acinetobacter baumannii*	54.0	0,000007

Query= MT-1_S8_L001_R1_001_(paired)_trimmed_(paired)_contig_41
Length=16124

Sequences producing significant alignments	IS Family	Group	Origin	Score (bits)	E. value
ISAba1	*IS4*	*IS10*	*Acinetobacter baumannii*	180	5E-44
ISAba33	*IS4*	*IS10*	*Acinetobacter baumannii*	111	4E-23
ISPpu12	*ISL3*		*Pseudomonas putida*	103	9E-21

Query= MT-1_S8_L001_R1_001_(paired)_trimmed_(paired)_contig_43
Length=11589

Sequences producing significant alignments	IS Family	Group	Origin	Score (bits)	E. value
ISAba125	*IS30*		*Acinetobacter baumannii*	119	1E-25
ISC1041	*IS30*		*Sulfolobus solfataricus*	103	6E-21

Query= MT-1_S8_L001_R1_001_(paired)_trimmed_(paired)_contig_44
Length=10415

Sequences producing significant alignments	IS Family	Group	Origin	Score (bits)	E. value
ISAba13	*IS5*	*IS903*	*Acinetobacter baumannii*	119	9E-26
ISAjo1	*IS5*	*IS903*	*Acinetobacter johnsonii*	52.0	0,00002
ISAha2	*IS5*	*IS903*	*Acinetobacter haemolyticus*	52.0	0,00002
ISAlw1	*IS5*	*IS903*	*Acinetobacter lwoffii*	50.1	0,00007

Query= MT-1_S8_L001_R1_001_(paired)_trimmed_(paired)_contig_45
Length=10272

Sequences producing significant alignments	IS Family	Group	Origin	Score (bits)	E. value
ISPpu12	*ISL3*		*Pseudomonas putida*	103	6E-21

Query= MT-1_S8_L001_R1_001_(paired)_trimmed_(paired)_contig_47
Length=7773

Sequences producing significant alignments	IS Family	Group	Origin	Score (bits)	E. value
ISAba1	*IS4*	*IS10*	*Acinetobacter baumannii*	131	2E-29
ISAjo2	*ISNCY*	*IS1202*	*Acinetobacter johnsonii*	119	7E-26
ISAba33	*IS4*	*IS10*	*Acinetobacter baumannii*	69.9	6E-11

Query= MT-1_S8_L001_R1_001_(paired)_trimmed_(paired)_contig_48
Length=7724

Sequences producing significant alignments	IS Family	Group	Origin	Score (bits)	E. value
ISAba1	*IS4*	*IS10*	*Acinetobacter baumannii*	111	2E-23
ISAba33	*IS4*	*IS10*	*Acinetobacter baumannii*	63.9	0,000000004

Query= MT-1_S8_L001_R1_001_(paired)_trimmed_(paired)_contig_49
Length=7630

Sequences producing significant alignments	IS Family	Group	Origin	Score (bits)	E. value
ISAba125	*IS30*		*Acinetobacter baumannii*	1826	0.0
TnAs3	*Tn3*		*Aeromonas salmonicida*	1364	0.0
ISC1041	*IS30*		*Sulfolobus solfataricus*	668	0.0
ISAbe8	*IS30*		*Acinetobacter bereziniae*	317	1E-85
IS15DI	*IS6*		*Salmonella panama*	119	7E-26
IS15	*IS6*		*Salmonella panama*	119	7E-26
IS26	*IS6*		*Proteus vulgaris*	119	7E-26
IS15DIV	*IS6*		*Salmonella typhimurium*	117	3E-25
IS15DII	*IS6*		*Salmonella panama*	111	2E-23

Query= MT-1_S8_L001_R1_001_(paired)_trimmed_(paired)_contig_52
Length=5288

Sequences producing significant alignments	IS Family	Group	Origin	Score (bits)	E. value
ISAlw34	*IS66*		*Acinetobacter lwoffii*	198	6E-50
ISAba46	*IS66*		*Acinetobacter baumannii*	198	6E-50
ISAba16	*IS66*		*Acinetobacter baumannii*	192	4E-48
ISAlw16	*IS66*		*Acinetobacter lwoffii*	190	2E-47
ISAba25	*IS66*		*Acinetobacter baumannii*	167	2E-40
ISAjo2	*ISNCY*	*IS1202*	*Acinetobacter johnsonii*	131	1E-29
ISAba49	*IS66*		*Acinetobacter baumannii*	121	1E-26
ISAba17	*IS66*		*Acinetobacter baumannii*	58.0	0,0000002
ISSod17	*IS3*	*IS51*	*Shewanella oneidensis*	52.0	0,000009

Query= MT-1_S8_L001_R1_001_(paired)_trimmed_(paired)_contig_53
Length=5191

Sequences producing significant alignments	IS Family	Group	Origin	Score (bits)	E. value
ISAlw22	*ISNCY*	*IS1202*	*Acinetobacter lwoffii*	1891	0.0
ISAba32	*ISNCY*	*IS1202*	*Acinetobacter baumannii*	1891	0.0
ISAjo2	*ISNCY*	*IS1202*	*Acinetobacter johnsonii*	194	1E-48
ISEsa1	*ISNCY*	*IS1202*	*Enterobacter sakazakii*	60.0	0,00000004
ISV ISp7	*ISNCY*	*IS1202*	*Vibrio splendidus*	50.1	0,00004

Query= MT-1_S8_L001_R1_001_(paired)_trimmed_(paired)_contig_55
Length=4589

Sequences producing significant alignments	IS Family	Group	Origin	Score (bits)	E. value
TnAs3	*Tn3*		*Aeromonas salmonicida*	2575	0.0
TnAs2	*Tn3*		*Aeromonas salmonicida*	270	2E-71
ISShes11	*Tn3*		*Shewanella sp.*	182	3E-45
IS15DI	*IS6*		*Salmonella panama*	119	4E-26
IS15	*IS6*		*Salmonella panama*	119	4E-26
IS26	*IS6*		*Proteus vulgaris*	119	4E-26
IS15DIV	*IS6*		*Salmonella typhimurium*	117	2E-25
IS15DII	*IS6*		*Salmonella panama*	111	1E-23

Query= MT-1_S8_L001_R1_001_(paired)_trimmed_(paired)_contig_57
Length=4540

Sequences producing significant alignments	IS Family	Group	Origin	Score (bits)	E. value
TnAs3	*Tn3*		*Aeromonas salmonicida*	119	4E-26
ISPpu12	*ISL3*		*Pseudomonas putida*	119	4E-26
IS1396	*ISL3*		*Serratia marcescens*	119	4E-26
ISPst2	*ISL3*		*Pseudomonas stutzeri*	85.7	6E-16

ISPst9	ISL3		Pseudomonas stutzeri	63.9	0,000000002

Query= MT-1_S8_L001_R1_001_(paired)_trimmed_(paired)_contig_58
Length=4354

Sequences producing significant alignments	IS Family	Group	Origin	Score (bits)	E. value
ISAba14	IS3	IS150	Acinetobacter baumannii	605	2E-172
ISCfr1	IS1182		Citrobacter freundii	577	6E-164
ISPsp5	IS1182		Pseudomonas sp.	454	6E-127
ISAlw27	IS3	IS150	Acinetobacter lwoffii	383	2E-105
ISPa94	IS1182		Pseudomonas aeruginosa	309	2E-83
IS15DI	IS6		Salmonella panama	119	4E-26
IS15	IS6		Salmonella panama	119	4E-26
IS26	IS6		Proteus vulgaris	119	4E-26
ISAba52	IS6		Acinetobacter baumannii	117	2E-25
IS1008	IS6		Acinetobacter calcoaceticus	117	2E-25
ISOur1	IS6		Oligella urethralis	117	2E-25
IS15DIV	IS6		Salmonella typhimurium	117	2E-25
IS1007	IS6		Acinetobacter sp.	117	2E-25
IS15DII	IS6		Salmonella panama	111	1E-23
ISAcsp5	IS3	IS150	Acinetobacter sp.	58.0	0,0000001

Query= MT-1_S8_L001_R1_001_(paired)_trimmed_(paired)_contig_59
Length=3953

Sequences producing significant alignments	IS Family	Group	Origin	Score (bits)	E. value
ISAlw4	IS3	IS51	Acinetobacter lwoffii	1875	0.0
ISAba2	IS3	IS51	Acinetobacter baumannii	1574	0.0
ISAlw23	IS3	IS51	Acinetobacter lwoffii	1538	0.0
ISAba19	IS3	IS51	Acinetobacter baumannii	1463	0.0
ISAlw5	IS3	IS51	Acinetobacter lwoffii	1439	0.0
ISAba29	IS3	IS51	Acinetobacter baumannii	1352	0.0
ISAba34	IS3	IS51	Acinetobacter baumannii	1308	0.0
ISAba18	IS3	IS51	Acinetobacter baumannii	1304	0.0
IS1008	IS6		Acinetobacter calcoaceticus	504	6E-142
ISOur1	IS6		Oligella urethralis	448	3E-125
ISAba52	IS6		Acinetobacter baumannii	432	2E-120
IS1006	IS6		Acinetobacter junii	424	4E-118
IS1007	IS6		Acinetobacter sp.	129	4E-29
IS15DIV	IS6		Salmonella typhimurium	95.6	5E-19
IS15DII	IS6		Salmonella panama	95.6	5E-19
IS15DI	IS6		Salmonella panama	95.6	5E-19
IS15	IS6		Salmonella panama	95.6	5E-19
IS26	IS6		Proteus vulgaris	95.6	5E-19
IS1489v1	ISL3		Pseudomonas putida	83.8	2E-15
ISPpu33	IS3	IS51	Pseudomonas putida	56.0	0,0000004
ISStma17	IS3	IS51	Stenotrophomonas maltophilia	56.0	0,0000004
ISMca3	IS3	IS51	Methylococcus capsulatus	56.0	0,0000004
IS1416	IS3	IS51	Burkholderia glumae	56.0	0,0000004
ISAli4	IS3	IS51	Azospirillum lipoferum	54.0	0,000002
ISRme15	IS3	IS51	Ralstonia metallidurans	54.0	0,000002
ISPmi1	IS3	IS51	Proteus mirabilis	52.0	0,000007
ISRtr2	IS3	IS51	Rhizobium tropici	52.0	0,000007

Query= MT-1_S8_L001_R1_001_(paired)_trimmed_(paired)_contig_60
Length=3945

Sequences producing significant alignments	IS Family	Group	Origin	Score (bits)	E. value
ISAba1	IS4	IS10	Acinetobacter baumannii	180	1E-44
ISAba125	IS30		Acinetobacter baumannii	119	4E-26
ISAba33	IS4	IS10	Acinetobacter baumannii	111	9E-24
ISC1041	IS30		Sulfolobus solfataricus	103	2E-21

Query= MT-1_S8_L001_R1_001_(paired)_trimmed_(paired)_contig_61
Length=3688

Sequences producing significant alignments	IS Family	Group	Origin	Score (bits)	E. value
Tn5393	Tn3		Erwinia amylovora	1358	0.0
ISEc63	Tn3		Escherichia coli	307	7E-83
Tn3	Tn3		Salmonella enterica	121	8E-27
IS15DIV	IS6		Salmonella typhimurium	119	3E-26
IS15DII	IS6		Salmonella panama	119	3E-26
IS15DI	IS6		Salmonella panama	119	3E-26
IS15	IS6		Salmonella panama	119	3E-26
IS26	IS6		Proteus vulgaris	119	3E-26
Tn2	Tn3		Escherichia coli	105	5E-22
ISMex22	Tn3		Methylobacterium extorquens	54.0	0,000002

Query= MT-1_S8_L001_R1_001_(paired)_trimmed_(paired)_contig_62
Length=3425

Sequences producing significant alignments	IS Family	Group	Origin	Score (bits)	E. value
ISAjo2	ISNCY	IS1202	Acinetobacter johnsonii	119	3E-26

Query= MT-1_S8_L001_R1_001_(paired)_trimmed_(paired)_contig_63
Length=3231

Sequences producing significant alignments	IS Family	Group	Origin	Score (bits)	E. value
ISPpu12	*ISL3*		*Pseudomonas putida*	5525	0.0
IS1396	*ISL3*		*Serratia marcescens*	2603	0.0
ISPst2	*ISL3*		*Pseudomonas stutzeri*	2325	0.0
ISPst9	*ISL3*		*Pseudomonas stutzeri*	1626	0.0
IS1489v1	*ISL3*		*Pseudomonas putida*	46.1	0,0003

Query= MT-1_S8_L001_R1_001_(paired)_trimmed_(paired)_contig_64
Length=3053

Sequences producing significant alignments	IS Family	Group	Origin	Score (bits)	E. value
ISAjo2	*ISNCY*	*IS1202*	*Acinetobacter johnsonii*	119	3E-26
IS15DIV	*IS6*		*Salmonella typhimurium*	119	3E-26
IS15DII	*IS6*		*Salmonella panama*	119	3E-26
IS15DI	*IS6*		*Salmonella panama*	119	3E-26
IS15	*IS6*		*Salmonella panama*	119	3E-26
IS26	*IS6*		*Proteus vulgaris*	119	3E-26

Query= MT-1_S8_L001_R1_001_(paired)_trimmed_(paired)_contig_66
Length=2786

Sequences producing significant alignments	IS Family	Group	Origin	Score (bits)	E. value
IS1008	*IS6*		*Acinetobacter calcoaceticus*	519	7E-147
ISOur1	*IS6*		*Oligella urethralis*	464	4E-130
ISAba52	*IS6*		*Acinetobacter baumannii*	448	2E-125
IS1006	*IS6*		*Acinetobacter junii*	440	5E-123
IS1007	*IS6*		*Acinetobacter sp.*	145	4E-34
IS15DIV	*IS6*		*Salmonella typhimurium*	87.7	9E-17
IS15DII	*IS6*		*Salmonella panama*	87.7	9E-17
IS15DI	*IS6*		*Salmonella panama*	87.7	9E-17
IS15	*IS6*		*Salmonella panama*	87.7	9E-17
IS26	*IS6*		*Proteus vulgaris*	87.7	9E-17
IS1489v1	*ISL3*		*Pseudomonas putida*	83.8	1E-15

Query= MT-1_S8_L001_R1_001_(paired)_trimmed_(paired)_contig_68
Length=2286

Sequences producing significant alignments	IS Family	Group	Origin	Score (bits)	E. value
ISPpu12	*ISL3*		*Pseudomonas putida*	361	3E-99
TnAs3	*Tn3*		*Aeromonas salmonicida*	119	2E-26
ISXc4	*Tn3*		*Xanthomonas campestris*	73.8	1E-12
ISPsy42	*Tn3*		*Pseudomonas syringae*	63.9	0,000000001
ISThsp9	*Tn3*		*Thiomonas sp.*	54.0	0,000001
ISMex22	*Tn3*		*Methylobacterium extorquens*	52.0	0,000004

Query= MT-1_S8_L001_R1_001_(paired)_trimmed_(paired)_contig_71
Length=1597

Sequences producing significant alignments	IS Family	Group	Origin	Score (bits)	E. value
TnAs3	*Tn3*		*Aeromonas salmonicida*	3166	0.0

Query= MT-1_S8_L001_R1_001_(paired)_trimmed_(paired)_contig_72
Length=1570

Sequences producing significant alignments	IS Family	Group	Origin	Score (bits)	E. value
TnAs3	*Tn3*		*Aeromonas salmonicida*	860	0.0
ISAba125	*IS30*		*Acinetobacter baumannii*	432	7E-121
ISC1041	*IS30*		*Sulfolobus solfataricus*	335	1E-91
IS15DIV	*IS6*		*Salmonella typhimurium*	119	1E-26
IS15DII	*IS6*		*Salmonella panama*	119	1E-26
IS15DI	*IS6*		*Salmonella panama*	119	1E-26
IS15	*IS6*		*Salmonella panama*	119	1E-26
IS26	*IS6*		*Proteus vulgaris*	119	1E-26
ISAbe8	*IS30*		*Acinetobacter bereziniae*	58.0	0,00000004

Query= MT-1_S8_L001_R1_001_(paired)_trimmed_(paired)_contig_73
Length=1549

Sequences producing significant alignments	IS Family	Group	Origin	Score (bits)	E. value
IS15	*IS6*		*Salmonella panama*	466	5E-131
IS15DII	*IS6*		*Salmonella panama*	347	3E-95
IS15DI	*IS6*		*Salmonella panama*	339	8E-93
IS26	*IS6*		*Proteus vulgaris*	339	8E-93
IS15DIV	*IS6*		*Salmonella typhimurium*	337	3E-92
ISAba52	*IS6*		*Acinetobacter baumannii*	61.9	0,000000003
IS1008	*IS6*		*Acinetobacter calcoaceticus*	61.9	0,000000003
ISOur1	*IS6*		*Oligella urethralis*	61.9	0,000000003

IS1006	IS6		Acinetobacter junii	61.9	0,000000003
IS1007	IS6		Acinetobacter sp.	52.0	0,000003

Query= MT-1_S8_L001_R1_001_(paired)_trimmed_(paired)_contig_74
Length=1476

Sequences producing significant alignments	IS Family	Group	Origin	Score (bits)	E. value
ISAjo2	ISNCY	IS1202	Acinetobacter johnsonii	2268	0.0
ISAlw22	ISNCY	IS1202	Acinetobacter lwoffii	170	4E-42
ISAba32	ISNCY	IS1202	Acinetobacter baumannii	159	2E-38
ISEsa1	ISNCY	IS1202	Enterobacter sakazakii	65.9	2E-10
ISLad2	ISNCY	IS1202	Leclercia adecarboxylata	65.9	2E-10
ISCARN112	ISNCY	IS1202	Metagenomic data	48.1	0,00004

Query= MT-1_S8_L001_R1_001_(paired)_trimmed_(paired)_contig_76
Length=1139

Sequences producing significant alignments	IS Family	Group	Origin	Score (bits)	E. value
ISAba1	IS4	IS10	Acinetobacter baumannii	2250	0.0
ISAba33	IS4	IS10	Acinetobacter baumannii	1219	0.0

Query= MT-1_S8_L001_R1_001_(paired)_trimmed_(paired)_contig_78
Length=1039

Sequences producing significant alignments	IS Family	Group	Origin	Score (bits)	E. value
ISAba13	IS5	IS903	Acinetobacter baumannii	2060	0.0
ISAba12	IS5	IS903	Acinetobacter baumannii	848	0.0
IS17	IS5	IS903	Acinetobacter haemolyticus	835	0.0
ISAba40	IS5	IS903	Acinetobacter baumannii	803	0.0
ISAha1	IS5	IS903	Acinetobacter haemolyticus	801	0.0
ISAlw1	IS5	IS903	Acinetobacter lwoffii	704	0.0
ISAjo1	IS5	IS903	Acinetobacter johnsonii	636	0.0
ISAba53	IS5	IS903	Acinetobacter baumannii	617	2E-176
ISAha3	IS5	IS903	Acinetobacter haemolyticus	517	1E-146
ISAha2	IS5	IS903	Acinetobacter haemolyticus	428	7E-120
ISAba7	IS5	IS903	Acinetobacter baumannii	420	2E-117
ISAba5	IS5	IS903	Acinetobacter baumannii	387	3E-107

Query= MT-1_S8_L001_R1_001_(paired)_trimmed_(paired)_contig_79
Length=905

Sequences producing significant alignments	IS Family	Group	Origin	Score (bits)	E. value
ISAba125	IS30		Acinetobacter baumannii	119	8E-27
ISC1041	IS30		Sulfolobus solfataricus	103	5E-22
ISAbe8	IS30		Acinetobacter bereziniae	44.1	0,0004

Query= MT-1_S8_L001_R1_001_(paired)_trimmed_(paired)_contig_81
Length=820

Sequences producing significant alignments	IS Family	Group	Origin	Score (bits)	E. value
IS15DI	IS6		Salmonella panama	1626	0.0
IS15	IS6		Salmonella panama	1626	0.0
IS15DIV	IS6		Salmonella typhimurium	1608	0.0
IS26	IS6		Proteus vulgaris	1602	0.0
IS15DII	IS6		Salmonella panama	1594	0.0
IS1006	IS6		Acinetobacter junii	180	2E-45
ISAba52	IS6		Acinetobacter baumannii	137	3E-32
IS1007	IS6		Acinetobacter sp.	137	3E-32
ISOur1	IS6		Oligella urethralis	121	2E-27
IS1008	IS6		Acinetobacter calcoaceticus	113	4E-25
ISStag1	IS6		Stappia aggregata	65.9	9E-11
ISAcr2	IS6		Acidiphilium cryptum	58.0	0,00000002

Query= MT-1_S8_L001_R1_001_(paired)_trimmed_(paired)_contig_82
Length=662

Sequences producing significant alignments	IS Family	Group	Origin	Score (bits)	E. value
ISAba1	IS4	IS10	Acinetobacter baumannii	180	2E-45
ISAjo2	ISNCY	IS1202	Acinetobacter johnsonii	131	2E-30
ISAba33	IS4	IS10	Acinetobacter baumannii	111	1E-24
ISCARN112	ISNCY	IS1202	Metagenomic data	48.1	0,00002

Query= MT-1_S8_L001_R1_001_(paired)_trimmed_(paired)_contig_83
Length=619

Sequences producing significant alignments	IS Family	Group	Origin	Score (bits)	E. value
IS1008	IS6		Acinetobacter calcoaceticus	1209	0.0
ISOur1	IS6		Oligella urethralis	1162	0.0
ISAba52	IS6		Acinetobacter baumannii	662	0.0
IS1007	IS6		Acinetobacter sp.	525	3E-149
IS1006	IS6		Acinetobacter junii	502	4E-142

IS15DIV	IS6		*Salmonella typhimurium*	113	3E-25
IS15DII	IS6		*Salmonella panama*	113	3E-25
IS15DI	IS6		*Salmonella panama*	113	3E-25
IS15	IS6		*Salmonella panama*	113	3E-25
IS26	IS6		*Proteus vulgaris*	113	3E-25

Query= MT-1_S8_L001_R1_001_(paired)_trimmed_(paired)_contig_86
Length=550

Sequences producing significant alignments	*IS Family*	*Group*	*Origin*	Score (bits)	E. value
ISAba125	IS30		*Acinetobacter baumannii*	119	5E-27
ISC1041	IS30		*Sulfolobus solfataricus*	103	3E-22
ISAbe8	IS30		*Acinetobacter bereziniae*	46.1	0,00006

Query= MT-1_S8_L001_R1_001_(paired)_trimmed_(paired)_contig_88
Length=450

Sequences producing significant alignments	*IS Family*	*Group*	*Origin*	Score (bits)	E. value
ISAba125	IS30		*Acinetobacter baumannii*	876	0.0
ISC1041	IS30		*Sulfolobus solfataricus*	581	4E-166
ISAbe8	IS30		*Acinetobacter bereziniae*	196	2E-50

Query= MT-1_S8_L001_R1_001_(paired)_trimmed_(paired)_contig_92
Length=326

Sequences producing significant alignments	*IS Family*	*Group*	*Origin*	Score (bits)	E. value
ISAba1	IS4	IS10	*Acinetobacter baumannii*	638	0.0
ISAba33	IS4	IS10	*Acinetobacter baumannii*	416	9E-117

Query= MT-1_S8_L001_R1_001_(paired)_trimmed_(paired)_contig_98
Length=267

Sequences producing significant alignments	*IS Family*	*Group*	*Origin*	Score (bits)	E. value
ISAjo2	ISNCY	IS1202	*Acinetobacter johnsonii*	355	2E-98
ISAlw22	ISNCY	IS1202	*Acinetobacter lwoffii*	54.0	0,0000001
ISAba32	ISNCY	IS1202	*Acinetobacter baumannii*	54.0	0,0000001

Query= MT-1_S8_L001_R1_001_(paired)_trimmed_(paired)_contig_110
Length=222

Sequences producing significant alignments	*IS Family*	*Group*	*Origin*	Score (bits)	E. value
ISAba125	IS30		*Acinetobacter baumannii*	440	4E-124
ISC1041	IS30		*Sulfolobus solfataricus*	327	4E-90
ISAbe8	IS30		*Acinetobacter bereziniae*	58.0	0,000000006

Query= MT-1_S8_L001_R1_001_(paired)_trimmed_(paired)_contig_111
Length=220

Sequences producing significant alignments	*IS Family*	*Group*	*Origin*	Score (bits)	E. value
ISAba1	IS4	IS10	*Acinetobacter baumannii*	180	6E-46
ISAba33	IS4	IS10	*Acinetobacter baumannii*	111	5E-25

Query= MT-1_S8_L001_R1_001_(paired)_trimmed_(paired)_contig_116
Length=204

Sequences producing significant alignments	*IS Family*	*Group*	*Origin*	Score (bits)	E. value
TnAs1	Tn3		*Aeromonas salmonicida*	250	7E-67
ISPa40	Tn3		*Pseudomonas aeruginosa*	170	5E-43

Query= MT-1_S8_L001_R1_001_(paired)_trimmed_(paired)_contig_117
Length=203

Sequences producing significant alignments	*IS Family*	*Group*	*Origin*	Score (bits)	E. value
ISAjo2	ISNCY	IS1202	*Acinetobacter johnsonii*	228	3E-60
ISAlw22	ISNCY	IS1202	*Acinetobacter lwoffii*	54.0	0,00000008
ISAba32	ISNCY	IS1202	*Acinetobacter baumannii*	54.0	0,00000008

Query= MT-1_S8_L001_R1_001_(paired)_trimmed_(paired)_contig_121
Length=268

Sequences producing significant alignments	*IS Family*	*Group*	*Origin*	Score (bits)	E. value
ISAjo2	ISNCY	IS1202	*Acinetobacter johnsonii*	131	6E-31
ISCARN112	ISNCY	IS1202	*Metagenomic data*	48.1	0,000007

Database: ISfinder_Nucl
5,559 sequences; 8,208,285 total letters

Query= MT-2_S8_L001_R1_001_(paired)_trimmed_(paired)_contig_1
Length=308335

Sequences producing significant alignments	*IS Family*	*Group*	*Origin*	*Score (bits)*	*E. value*
ISAjo2	IS*NCY*	*IS1202*	*Acinetobacter johnsonii*	151	8E-34
ISAba1	*IS4*	*IS10*	*Acinetobacter baumannii*	129	3E-27
ISAba33	*IS4*	*IS10*	*Acinetobacter baumannii*	69.9	0,000000002

Query= MT-2_S8_L001_R1_001_(paired)_trimmed_(paired)_contig_3
Length=202737

Sequences producing significant alignments	*IS Family*	*Group*	*Origin*	*Score (bits)*	*E. value*
ISAba1	*IS4*	*IS10*	*Acinetobacter baumannii*	248	3E-63
ISAba33	*IS4*	*IS10*	*Acinetobacter baumannii*	163	1E-37
ISAba13	*IS5*	*IS903*	*Acinetobacter baumannii*	119	2E-24
ISAha1	*IS5*	*IS903*	*Acinetobacter haemolyticus*	111	4E-22
ISAba53	*IS5*	*IS903*	*Acinetobacter baumannii*	103	1E-19
IS17	*IS5*	*IS903*	*Acinetobacter haemolyticus*	77.8	6E-12
ISAba12	*IS5*	*IS903*	*Acinetobacter baumannii*	69.9	0,000000002
ISAba40	*IS5*	*IS903*	*Acinetobacter baumannii*	56.0	0,00002
ISAjo1	*IS5*	*IS903*	*Acinetobacter johnsonii*	54.0	0,00009
ISAba5	*IS5*	*IS903*	*Acinetobacter baumannii*	54.0	0,00009

Query= MT-2_S8_L001_R1_001_(paired)_trimmed_(paired)_contig_4
Length=200470

Sequences producing significant alignments	*IS Family*	*Group*	*Origin*	*Score (bits)*	*E. value*
ISAba1	*IS4*	*IS10*	*Acinetobacter baumannii*	248	3E-63
ISAba33	*IS4*	*IS10*	*Acinetobacter baumannii*	163	1E-37
ISPpu12	*ISL3*		*Pseudomonas putida*	131	5E-28
IS1396	*ISL3*		*Serratia marcescens*	131	5E-28
ISPst2	*ISL3*		*Pseudomonas stutzeri*	85.7	3E-14
ISPst9	*ISL3*		*Pseudomonas stutzeri*	75.8	2E-11

Query= MT-2_S8_L001_R1_001_(paired)_trimmed_(paired)_contig_6
Length=184508

Sequences producing significant alignments	*IS Family*	*Group*	*Origin*	*Score (bits)*	*E. value*
ISAba1	*IS4*	*IS10*	*Acinetobacter baumannii*	129	2E-27
ISAba13	*IS5*	*IS903*	*Acinetobacter baumannii*	119	2E-24
ISAha1	*IS5*	*IS903*	*Acinetobacter haemolyticus*	111	4E-22
ISAba53	*IS5*	*IS903*	*Acinetobacter baumannii*	103	1E-19
IS17	*IS5*	*IS903*	*Acinetobacter haemolyticus*	77.8	6E-12
ISAba33	*IS4*	*IS10*	*Acinetobacter baumannii*	69.9	0,000000001
ISAba12	*IS5*	*IS903*	*Acinetobacter baumannii*	69.9	0,000000001
ISAba40	*IS5*	*IS903*	*Acinetobacter baumannii*	56.0	0,00002
ISAjo1	*IS5*	*IS903*	*Acinetobacter johnsonii*	54.0	0,00008
ISAba5	*IS5*	*IS903*	*Acinetobacter baumannii*	54.0	0,00008

Query= MT-2_S8_L001_R1_001_(paired)_trimmed_(paired)_contig_7
Length=170837

Sequences producing significant alignments	*IS Family*	*Group*	*Origin*	*Score (bits)*	*E. value*
ISAba1	*IS4*	*IS10*	*Acinetobacter baumannii*	129	2E-27
ISAba33	*IS4*	*IS10*	*Acinetobacter baumannii*	69.9	0,000000001

Query= MT-2_S8_L001_R1_001_(paired)_trimmed_(paired)_contig_8
Length=163458

Sequences producing significant alignments	*IS Family*	*Group*	*Origin*	*Score (bits)*	*E. value*
ISAba1	*IS4*	*IS10*	*Acinetobacter baumannii*	248	2E-63
ISAjo2	IS*NCY*	*IS1202*	*Acinetobacter johnsonii*	186	8E-45
ISAba33	*IS4*	*IS10*	*Acinetobacter baumannii*	163	1E-37

Query= MT-2_S8_L001_R1_001_(paired)_trimmed_(paired)_contig_9
Length=153580

Sequences producing significant alignments	*IS Family*	*Group*	*Origin*	*Score (bits)*	*E. value*
ISAjo2	IS*NCY*	*IS1202*	*Acinetobacter johnsonii*	151	4E-34
ISAba13	*IS5*	*IS903*	*Acinetobacter baumannii*	121	4E-25
ISAlw34	*IS66*		*Acinetobacter lwoffii*	58.0	0,000004
ISAlw16	*IS66*		*Acinetobacter lwoffii*	58.0	0,000004
ISAba16	*IS66*		*Acinetobacter baumannii*	58.0	0,000004
ISAba17	*IS66*		*Acinetobacter baumannii*	58.0	0,000004

Query= MT-2_S8_L001_R1_001_(paired)_trimmed_(paired)_contig_10
Length=148266

Sequences producing significant alignments	*IS Family*	*Group*	*Origin*	*Score (bits)*	*E. value*
ISAba1	*IS4*	*IS10*	*Acinetobacter baumannii*	248	2E-63
ISAba33	*IS4*	*IS10*	*Acinetobacter baumannii*	163	1E-37
ISAba13	*IS5*	*IS903*	*Acinetobacter baumannii*	119	1E-24
ISAha1	*IS5*	*IS903*	*Acinetobacter haemolyticus*	111	3E-22
ISAba53	*IS5*	*IS903*	*Acinetobacter baumannii*	103	8E-20
IS17	*IS5*	*IS903*	*Acinetobacter haemolyticus*	77.8	5E-12
ISAba12	*IS5*	*IS903*	*Acinetobacter baumannii*	69.9	0,000000001
ISAba40	*IS5*	*IS903*	*Acinetobacter baumannii*	56.0	0,00002
ISAjo1	*IS5*	*IS903*	*Acinetobacter johnsonii*	54.0	0,00007
ISAba5	*IS5*	*IS903*	*Acinetobacter baumannii*	54.0	0,00007

Query= MT-2_S8_L001_R1_001_(paired)_trimmed_(paired)_contig_11
Length=140873

Sequences producing significant alignments	IS Family	Group	Origin	Score (bits)	E. value
ISAba1	*IS4*	*IS10*	*Acinetobacter baumannii*	248	2E-63
ISAba33	*IS4*	*IS10*	*Acinetobacter baumannii*	163	9E-38
ISAjo2	*ISNCY*	*IS1202*	*Acinetobacter johnsonii*	151	4E-34

Query= MT-2_S8_L001_R1_001_(paired)_trimmed_(paired)_contig_12
Length=137934

Sequences producing significant alignments	IS Family	Group	Origin	Score (bits)	E. value
ISAba1	*IS4*	*IS10*	*Acinetobacter baumannii*	248	2E-63
ISAba33	*IS4*	*IS10*	*Acinetobacter baumannii*	163	9E-38
ISAba13	*IS5*	*IS903*	*Acinetobacter baumannii*	119	1E-24
ISAha1	*IS5*	*IS903*	*Acinetobacter haemolyticus*	111	3E-22
ISAba53	*IS5*	*IS903*	*Acinetobacter baumannii*	103	7E-20
IS17	*IS5*	*IS903*	*Acinetobacter haemolyticus*	77.8	4E-12
ISAba12	*IS5*	*IS903*	*Acinetobacter baumannii*	69.9	0,000000001
ISAba40	*IS5*	*IS903*	*Acinetobacter baumannii*	56.0	0,00002
ISAjo1	*IS5*	*IS903*	*Acinetobacter johnsonii*	54.0	0,00006
ISAba5	*IS5*	*IS903*	*Acinetobacter baumannii*	54.0	0,00006

Query= MT-2_S8_L001_R1_001_(paired)_trimmed_(paired)_contig_13
Length=125442

Sequences producing significant alignments	IS Family	Group	Origin	Score (bits)	E. value
ISAba1	*IS4*	*IS10*	*Acinetobacter baumannii*	248	2E-63
ISAba33	*IS4*	*IS10*	*Acinetobacter baumannii*	163	8E-38

Query= MT-2_S8_L001_R1_001_(paired)_trimmed_(paired)_contig_14
Length=120407

Sequences producing significant alignments	IS Family	Group	Origin	Score (bits)	E. value
ISAba13	*IS5*	*IS903*	*Acinetobacter baumannii*	2060	0.0
ISAba12	*IS5*	*IS903*	*Acinetobacter baumannii*	848	0.0
IS17	*IS5*	*IS903*	*Acinetobacter haemolyticus*	835	0.0
ISAba40	*IS5*	*IS903*	*Acinetobacter baumannii*	803	0.0
ISAha1	*IS5*	*IS903*	*Acinetobacter haemolyticus*	801	0.0
ISAlw1	*IS5*	*IS903*	*Acinetobacter lwoffii*	704	0.0
ISAjo1	*IS5*	*IS903*	*Acinetobacter johnsonii*	636	2E-180
ISAba53	*IS5*	*IS903*	*Acinetobacter baumannii*	617	2E-174
ISAha3	*IS5*	*IS903*	*Acinetobacter haemolyticus*	517	1E-144
ISAha2	*IS5*	*IS903*	*Acinetobacter haemolyticus*	428	9E-118
ISAba7	*IS5*	*IS903*	*Acinetobacter baumannii*	420	2E-115
ISAba5	*IS5*	*IS903*	*Acinetobacter baumannii*	387	3E-105

Query= MT-2_S8_L001_R1_001_(paired)_trimmed_(paired)_contig_15
Length=120155

Sequences producing significant alignments	IS Family	Group	Origin	Score (bits)	E. value
ISAba1	*IS4*	*IS10*	*Acinetobacter baumannii*	129	1E-27
ISAba33	*IS4*	*IS10*	*Acinetobacter baumannii*	69.9	9E-10

Query= MT-2_S8_L001_R1_001_(paired)_trimmed_(paired)_contig_16
Length=117863

Sequences producing significant alignments	IS Family	Group	Origin	Score (bits)	E. value
ISAjo2	*ISNCY*	*IS1202*	*Acinetobacter johnsonii*	186	5E-45

Query= MT-2_S8_L001_R1_001_(paired)_trimmed_(paired)_contig_17
Length=87981

Sequences producing significant alignments	IS Family	Group	Origin	Score (bits)	E. value
ISAba26	*IS256*		*Acinetobacter baumannii*	2605	0.0
ISAba13	*IS5*	*IS903*	*Acinetobacter baumannii*	121	2E-25
ISAbe10	*IS256*		*Acinetobacter bereziniae*	111	2E-22
ISAba42	*IS256*		*Acinetobacter baumannii*	83.8	4E-14
ISAba50	*IS256*		*Acinetobacter baumannii*	56.0	0,00001

Query= MT-2_S8_L001_R1_001_(paired)_trimmed_(paired)_contig_18
Length=75932

Sequences producing significant alignments	IS Family	Group	Origin	Score (bits)	E. value
ISAba13	*IS5*	*IS903*	*Acinetobacter baumannii*	119	7E-25
ISAha1	*IS5*	*IS903*	*Acinetobacter haemolyticus*	111	2E-22
ISAba53	*IS5*	*IS903*	*Acinetobacter baumannii*	103	4E-20
IS17	*IS5*	*IS903*	*Acinetobacter haemolyticus*	77.8	2E-12
ISAba12	*IS5*	*IS903*	*Acinetobacter baumannii*	69.9	6E-10
ISAba40	*IS5*	*IS903*	*Acinetobacter baumannii*	56.0	0,000009
ISAjo1	*IS5*	*IS903*	*Acinetobacter johnsonii*	54.0	0,00003
ISAba5	*IS5*	*IS903*	*Acinetobacter baumannii*	54.0	0,00003

Query= MT-2_S8_L001_R1_001_(paired)_trimmed_(paired)_contig_19
Length=74553

Sequences producing significant alignments	IS Family	Group	Origin	Score (bits)	E. value
ISAjo2	*ISNCY*	*IS1202*	*Acinetobacter johnsonii*	151	2E-34

Query= MT-2_S8_L001_R1_001_(paired)_trimmed_(paired)_contig_20
Length=73064

Sequences producing significant alignments	IS Family	Group	Origin	Score (bits)	E. value
ISAba1	*IS4*	*IS10*	*Acinetobacter baumannii*	129	7E-28
ISAba33	*IS4*	*IS10*	*Acinetobacter baumannii*	69.9	5E-10

Query= MT-2_S8_L001_R1_001_(paired)_trimmed_(paired)_contig_21
Length=68579

Sequences producing significant alignments	IS Family	Group	Origin	Score (bits)	E. value	
ISAjo2	ISNCY	IS1202	Acinetobacter johnsonii		151	2E-34

Query= MT-2_S8_L001_R1_001_(paired)_trimmed_(paired)_contig_22
Length=66289

Sequences producing significant alignments	IS Family	Group	Origin	Score (bits)	E. value	
ISAba1	IS4	IS10	Acinetobacter baumannii		129	6E-28
ISAba33	IS4	IS10	Acinetobacter baumannii	69.9		5E-10

Query= MT-2_S8_L001_R1_001_(paired)_trimmed_(paired)_contig_23
Length=53671

Sequences producing significant alignments	IS Family	Group	Origin	Score (bits)	E. value	
ISAba1	IS4	IS10	Acinetobacter baumannii		129	5E-28
ISAba33	IS4	IS10	Acinetobacter baumannii	69.9		4E-10

Query= MT-2_S8_L001_R1_001_(paired)_trimmed_(paired)_contig_24
Length=50233

Sequences producing significant alignments	IS Family	Group	Origin	Score (bits)	E. value	
ISAba125	IS30		Acinetobacter baumannii		119	5E-25
ISC1041	IS30		Sulfolobus solfataricus	61.9		0,00000009

Query= MT-2_S8_L001_R1_001_(paired)_trimmed_(paired)_contig_25
Length=50074

Sequences producing significant alignments	IS Family	Group	Origin	Score (bits)	E. value	
ISAba1	IS4	IS10	Acinetobacter baumannii		129	5E-28
ISAba33	IS4	IS10	Acinetobacter baumannii	69.9		4E-10

Query= MT-2_S8_L001_R1_001_(paired)_trimmed_(paired)_contig_26
Length=49801

Sequences producing significant alignments	IS Family	Group	Origin	Score (bits)	E. value	
ISAba1	IS4	IS10	Acinetobacter baumannii		129	5E-28
ISAba125	IS30		Acinetobacter baumannii		119	4E-25
ISAba33	IS4	IS10	Acinetobacter baumannii	69.9		4E-10
ISC1041	IS30		Sulfolobus solfataricus	61.9		0,00000009

Query= MT-2_S8_L001_R1_001_(paired)_trimmed_(paired)_contig_27
Length=44919

Sequences producing significant alignments	IS Family	Group	Origin	Score (bits)	E. value	
ISAjo2	ISNCY	IS1202	Acinetobacter johnsonii		186	2E-45

Query= MT-2_S8_L001_R1_001_(paired)_trimmed_(paired)_contig_28
Length=42689

Sequences producing significant alignments	IS Family	Group	Origin	Score (bits)	E. value	
ISAjo2	ISNCY	IS1202	Acinetobacter johnsonii		151	1E-34
ISAba13	IS5	IS903	Acinetobacter baumannii		121	1E-25
ISAjo1	IS5	IS903	Acinetobacter johnsonii	52.0		0,00007
ISAha2	IS5	IS903	Acinetobacter haemolyticus	52.0		0,00007

Query= MT-2_S8_L001_R1_001_(paired)_trimmed_(paired)_contig_29
Length=40868

Sequences producing significant alignments	IS Family	Group	Origin	Score (bits)	E. value	
ISAba1	IS4	IS10	Acinetobacter baumannii		248	6E-64
ISAba33	IS4	IS10	Acinetobacter baumannii		163	3E-38
ISAba13	IS5	IS903	Acinetobacter baumannii		121	9E-26
ISAjo1	IS5	IS903	Acinetobacter johnsonii	52.0		0,00007
ISAha2	IS5	IS903	Acinetobacter haemolyticus	52.0		0,00007

Query= MT-2_S8_L001_R1_001_(paired)_trimmed_(paired)_contig_31
Length=37160

Sequences producing significant alignments	IS Family	Group	Origin	Score (bits)	E. value	
ISAba1	IS4	IS10	Acinetobacter baumannii		248	5E-64
ISAjo2	ISNCY	IS1202	Acinetobacter johnsonii		186	2E-45
ISAba33	IS4	IS10	Acinetobacter baumannii		163	2E-38

Query= MT-2_S8_L001_R1_001_(paired)_trimmed_(paired)_contig_32
Length=36858

Sequences producing significant alignments	IS Family	Group	Origin	Score (bits)	E. value	
ISAba1	IS4	IS10	Acinetobacter baumannii		129	3E-28
ISAba13	IS5	IS903	Acinetobacter baumannii		119	3E-25
ISAha1	IS5	IS903	Acinetobacter haemolyticus		111	8E-23
ISAba53	IS5	IS903	Acinetobacter baumannii		103	2E-20
IS17	IS5	IS903	Acinetobacter haemolyticus	77.8		1E-12
ISAba33	IS4	IS10	Acinetobacter baumannii	69.9		3E-10
ISAba12	IS5	IS903	Acinetobacter baumannii	69.9		3E-10
ISAba40	IS5	IS903	Acinetobacter baumannii	56.0		0,000004
ISAjo1	IS5	IS903	Acinetobacter johnsonii	54.0		0,00002
ISAba5	IS5	IS903	Acinetobacter baumannii	54.0		0,00002

Query= MT-2_S8_L001_R1_001_(paired)_trimmed_(paired)_contig_33
Length=34472

Sequences producing	IS Family	Group		Score		

significant alignments			Origin	(bits)	E. value
ISAjo2	ISNCY	IS1202	*Acinetobacter johnsonii*	186	2E-45

Query= MT-2_S8_L001_R1_001_(paired)_trimmed_(paired)_contig_35
Length=30907

Sequences producing significant alignments	IS Family	Group	Origin	Score (bits)	E. value
ISAlw4	IS3	IS51	*Acinetobacter lwoffii*	2044	0.0
ISAlw5	IS3	IS51	*Acinetobacter lwoffii*	1941	0.0
ISAlw23	IS3	IS51	*Acinetobacter lwoffii*	1885	0.0
ISAba2	IS3	IS51	*Acinetobacter baumannii*	1721	0.0
ISAba29	IS3	IS51	*Acinetobacter baumannii*	1552	0.0
ISAba34	IS3	IS51	*Acinetobacter baumannii*	959	0.0
ISAba19	IS3	IS51	*Acinetobacter baumannii*	910	0.0
ISAba18	IS3	IS51	*Acinetobacter baumannii*	739	0.0
ISAba27	IS5	ISL2	*Acinetobacter baumannii*	345	3E-93
ISAlw1	IS5	IS903	*Acinetobacter lwoffii*	161	8E-38
ISAba13	IS5	IS903	*Acinetobacter baumannii*	153	2E-35
ISAha1	IS5	IS903	*Acinetobacter haemolyticus*	153	2E-35
ISAjo2	ISNCY	IS1202	*Acinetobacter johnsonii*	151	8E-35
ISAjo1	IS5	IS903	*Acinetobacter johnsonii*	127	1E-27
ISAlw35	IS481		*Acinetobacter lwoffii*	107	1E-21
ISAba40	IS5	IS903	*Acinetobacter baumannii*	107	1E-21
ISAlw24	IS481		*Acinetobacter lwoffii*	99.6	3E-19
ISAba53	IS5	IS903	*Acinetobacter baumannii*	95.6	4E-18
ISSde2	IS481		*Shewanella denitrificans*	83.8	2E-14
ISAba7	IS5	IS903	*Acinetobacter baumannii*	83.8	2E-14
ISAha3	IS5	IS903	*Acinetobacter haemolyticus*	69.9	2E-10
ISAba12	IS5	IS903	*Acinetobacter baumannii*	69.9	2E-10
IS1006	IS6		*Acinetobacter junii*	63.9	0,00000001
ISRtr2	IS3	IS51	*Rhizobium tropici*	58.0	0,0000009
ISAba52	IS6		*Acinetobacter baumannii*	56.0	0,000003
ISPpu33	IS3	IS51	*Pseudomonas putida*	56.0	0,000003
ISStma17	IS3	IS51	*Stenotrophomonas maltophilia*	56.0	0,000003
ISMca3	IS3	IS51	*Methylococcus capsulatus*	56.0	0,000003
ISOur1	IS6		*Oligella urethralis*	56.0	0,000003
IS1416	IS3	IS51	*Burkholderia glumae*	56.0	0,000003
ISAli4	IS3	IS51	*Azospirillum lipoferum*	54.0	0,00001
ISRme15	IS3	IS51	*Ralstonia metallidurans*	54.0	0,00001
ISPmi1	IS3	IS51	*Proteus mirabilis*	52.0	0,00005

Query= MT-2_S8_L001_R1_001_(paired)_trimmed_(paired)_contig_36
Length=30126

Sequences producing significant alignments	IS Family	Group	Origin	Score (bits)	E. value
ISAba13	IS5	IS903	*Acinetobacter baumannii*	121	7E-26
ISAjo1	IS5	IS903	*Acinetobacter johnsonii*	52.0	0,00005
ISAha2	IS5	IS903	*Acinetobacter haemolyticus*	52.0	0,00005

Query= MT-2_S8_L001_R1_001_(paired)_trimmed_(paired)_contig_38
Length=23224

Sequences producing significant alignments	IS Family	Group	Origin	Score (bits)	E. value
ISAjo2	ISNCY	IS1202	*Acinetobacter johnsonii*	151	6E-35
ISAba13	IS5	IS903	*Acinetobacter baumannii*	119	2E-25
ISAha1	IS5	IS903	*Acinetobacter haemolyticus*	111	5E-23
ISAba53	IS5	IS903	*Acinetobacter baumannii*	103	1E-20
IS17	IS5	IS903	*Acinetobacter haemolyticus*	77.8	7E-13
ISAba12	IS5	IS903	*Acinetobacter baumannii*	69.9	2E-10
ISAba40	IS5	IS903	*Acinetobacter baumannii*	56.0	0,000003
ISAjo1	IS5	IS903	*Acinetobacter johnsonii*	54.0	0,00001
ISAba5	IS5	IS903	*Acinetobacter baumannii*	54.0	0,00001

Query= MT-2_S8_L001_R1_001_(paired)_trimmed_(paired)_contig_40
Length=19221

Sequences producing significant alignments	IS Family	Group	Origin	Score (bits)	E. value
Tn2	Tn3		*Escherichia coli*	8851	0.0
Tn3	Tn3		*Salmonella enterica*	8806	0.0
TnAs1	Tn3		*Aeromonas salmonicida*	2962	0.0
TnAs2	Tn3		*Aeromonas salmonicida*	1838	0.0
ISPa40	Tn3		*Pseudomonas aeruginosa*	1758	0.0
IS6100	IS6		*Mycobacterium fortuitum*	1744	0.0
IS26	IS6		*Proteus vulgaris*	1626	0.0
IS15DII	IS6		*Salmonella panama*	1618	0.0
IS15DIV	IS6		*Salmonella typhimurium*	1616	0.0
IS15	IS6		*Salmonella panama*	1604	0.0
IS15DI	IS6		*Salmonella panama*	1602	0.0
IS1R	IS1		*Escherichia coli*	1451	0.0
IS1B	IS1		*Escherichia coli*	1435	0.0
IS1D	IS1		*Escherichia coli*	1419	0.0
IS1G	IS1		*Escherichia coli*	1396	0.0
IS1A	IS1		*Escherichia coli*	1396	0.0
IS1SD	IS1		*Shigella dysenteriae*	1388	0.0
IS1S	IS1		*Shigella sonnei*	1372	0.0
TnAs3	Tn3		*Aeromonas salmonicida*	1338	0.0
ISShes11	Tn3		*Shewanella sp.*	1322	0.0
ISPa38	Tn3		*Pseudomonas aeruginosa*	1164	0.0
ISSen9	IS1		*Salmonella enterica*	1150	0.0
IS1X2	IS1		*Escherichia vulneris*	1102	0.0
IS1X4	IS1		*Escherichia hermannii*	991	0.0
IS1X3	IS1		*Escherichia fergusonii*	912	0.0
IS1X1	IS1		*Shigella flexneri*	864	0.0
IS1F	IS1		*Escherichia coli*	864	0.0
ISKpn14	IS1		*Klebsiella pneumoniae*	729	0.0
TnShfr1	Tn3		*Shewanella frigidimarina*	549	6E-155
ISStma11	ISL3		*Stenotrophomonas maltophilia*	492	1E-137
ISEc63	Tn3		*Escherichia coli*	486	7E-136
ISSod9	Tn3		*Shewanella oneidensis*	371	3E-101
IS1006	IS6		*Acinetobacter junii*	172	1E-41
ISAba52	IS6		*Acinetobacter baumannii*	137	7E-31

IS1007	*IS6*		*Acinetobacter sp.*	137	7E-31
ISOur1	*IS6*		*Oligella urethralis*	121	4E-26
ISArsp6	*Tn3*		*Arthrobacter sp.*	117	7E-25
IS1008	*IS6*		*Acinetobacter calcoaceticus*	113	1E-23
ISAcsp1	*Tn3*		*Acinetobacter species*	75.8	2E-12
ISStag1	*IS6*		*Stappia aggregata*	71.9	4E-11
Tn5393	*Tn3*		*Erwinia amylovora*	65.9	0,000000002
ISSf1	*IS5*	*IS1031*	*Shigella flexneri*	61.9	0,00000004
ISAcr2	*IS6*		*Acidiphilium cryptum*	58.0	0,0000005

Query= MT-2_S8_L001_R1_001_(paired)_trimmed_(paired)_contig_41
Length=18940

Sequences producing significant alignments	IS Family	Group	Origin	Score (bits)	E. value
ISAba13	*IS5*	*IS903*	*Acinetobacter baumannii*	2060	0.0
ISAba12	*IS5*	*IS903*	*Acinetobacter baumannii*	848	0.0
IS17	*IS5*	*IS903*	*Acinetobacter haemolyticus*	835	0.0
ISAba40	*IS5*	*IS903*	*Acinetobacter baumannii*	803	0.0
ISAha1	*IS5*	*IS903*	*Acinetobacter haemolyticus*	801	0.0
ISAlw1	*IS5*	*IS903*	*Acinetobacter lwoffii*	704	0.0
ISAjo1	*IS5*	*IS903*	*Acinetobacter johnsonii*	636	0.0
ISAba53	*IS5*	*IS903*	*Acinetobacter baumannii*	617	3E-175
ISAha3	*IS5*	*IS903*	*Acinetobacter haemolyticus*	517	2E-145
ISAha2	*IS5*	*IS903*	*Acinetobacter haemolyticus*	428	1E-118
ISAba7	*IS5*	*IS903*	*Acinetobacter baumannii*	420	3E-116
ISAba5	*IS5*	*IS903*	*Acinetobacter baumannii*	387	5E-106

Query= MT-2_S8_L001_R1_001_(paired)_trimmed_(paired)_contig_42
Length=17554

Sequences producing significant alignments	IS Family	Group	Origin	Score (bits)	E. value
ISAba1	*IS4*	*IS10*	*Acinetobacter baumannii*	129	2E-28
ISAba13	*IS5*	*IS903*	*Acinetobacter baumannii*	121	4E-26
ISAba33	*IS4*	*IS10*	*Acinetobacter baumannii*	69.9	1E-10
ISAjo1	*IS5*	*IS903*	*Acinetobacter johnsonii*	52.0	0,00003
ISAha2	*IS5*	*IS903*	*Acinetobacter haemolyticus*	52.0	0,00003

Query= MT-2_S8_L001_R1_001_(paired)_trimmed_(paired)_contig_43
Length=16573

Sequences producing significant alignments	IS Family	Group	Origin	Score (bits)	E. value
ISAjo2	*ISNCY*	*IS1202*	*Acinetobacter johnsonii*	186	8E-46
ISAba13	*IS5*	*IS903*	*Acinetobacter baumannii*	119	1E-25
ISAha1	*IS5*	*IS903*	*Acinetobacter haemolyticus*	111	4E-23
ISAba53	*IS5*	*IS903*	*Acinetobacter baumannii*	103	9E-21
IS17	*IS5*	*IS903*	*Acinetobacter haemolyticus*	77.8	5E-13
ISAba12	*IS5*	*IS903*	*Acinetobacter baumannii*	69.9	1E-10
ISAba40	*IS5*	*IS903*	*Acinetobacter baumannii*	56.0	0,000002
ISAjo1	*IS5*	*IS903*	*Acinetobacter johnsonii*	54.0	0,000007
ISAba5	*IS5*	*IS903*	*Acinetobacter baumannii*	54.0	0,000007

Query= MT-2_S8_L001_R1_001_(paired)_trimmed_(paired)_contig_44
Length=16158

Sequences producing significant alignments	IS Family	Group	Origin	Score (bits)	E. value
ISAba1	*IS4*	*IS10*	*Acinetobacter baumannii*	248	2E-64
ISAba33	*IS4*	*IS10*	*Acinetobacter baumannii*	163	1E-38
ISPpu12	*ISL3*		*Pseudomonas putida*	103	9E-21

Query= MT-2_S8_L001_R1_001_(paired)_trimmed_(paired)_contig_45
Length=11589

Sequences producing significant alignments	IS Family	Group	Origin	Score (bits)	E. value
ISAba125	*IS30*		*Acinetobacter baumannii*	119	1E-25
ISC1041	*IS30*		*Sulfolobus solfataricus*	103	6E-21

Query= MT-2_S8_L001_R1_001_(paired)_trimmed_(paired)_contig_46
Length=10398

Sequences producing significant alignments	IS Family	Group	Origin	Score (bits)	E. value
ISAba13	*IS5*	*IS903*	*Acinetobacter baumannii*	121	2E-26
ISAjo1	*IS5*	*IS903*	*Acinetobacter johnsonii*	52.0	0,00002
ISAha2	*IS5*	*IS903*	*Acinetobacter haemolyticus*	52.0	0,00002
ISAlw1	*IS5*	*IS903*	*Acinetobacter lwoffii*	50.1	0,00007

Query= MT-2_S8_L001_R1_001_(paired)_trimmed_(paired)_contig_47
Length=10272

Sequences producing significant alignments	IS Family	Group	Origin	Score (bits)	E. value
ISPpu12	*ISL3*		*Pseudomonas putida*	103	6E-21

Query= MT-2_S8_L001_R1_001_(paired)_trimmed_(paired)_contig_48
Length=9499

Sequences producing significant alignments	IS Family	Group	Origin	Score (bits)	E. value
ISAba1	*IS4*	*IS10*	*Acinetobacter baumannii*	2323	0.0
ISAba33	*IS4*	*IS10*	*Acinetobacter baumannii*	1279	0.0
ISAjo2	*ISNCY*	*IS1202*	*Acinetobacter johnsonii*	186	4E-46

Query= MT-2_S8_L001_R1_001_(paired)_trimmed_(paired)_contig_49
Length=7724

Sequences producing significant alignments	IS Family	Group	Origin	Score (bits)	E. value
ISAba1	*IS4*	*IS10*	*Acinetobacter baumannii*	111	2E-23
ISAba33	*IS4*	*IS10*	*Acinetobacter baumannii*	63.9	0,000000004

Query= MT-2_S8_L001_R1_001_(paired)_trimmed_(paired)_contig_50
Length=6652

Sequences producing significant alignments	IS Family	Group	Origin	Score (bits)	E. value
TnAs3	Tn3		Aeromonas salmonicida	1364	0.0
IS15DI	IS6		Salmonella panama	119	6E-26
IS15	IS6		Salmonella panama	119	6E-26
IS26	IS6		Proteus vulgaris	119	6E-26
IS15DIV	IS6		Salmonella typhimurium	117	2E-25
IS15DII	IS6		Salmonella panama	111	1E-23

Query= MT-2_S8_L001_R1_001_(paired)_trimmed_(paired)_contig_52
Length=5379

Sequences producing significant alignments	IS Family	Group	Origin	Score (bits)	E. value
ISAlw22	ISNCY	IS1202	Acinetobacter lwoffii	1891	0.0
ISAba32	ISNCY	IS1202	Acinetobacter baumannii	1891	0.0
ISAjo2	ISNCY	IS1202	Acinetobacter johnsonii	325	4E-88
ISEsa1	ISNCY	IS1202	Enterobacter sakazakii	60.0	0,00000004
ISV ISp7	ISNCY	IS1202	Vibrio splendidus	50.1	0,00004

Query= MT-2_S8_L001_R1_001_(paired)_trimmed_(paired)_contig_53
Length=5310

Sequences producing significant alignments	IS Family	Group	Origin	Score (bits)	E. value
ISAlw34	IS66		Acinetobacter lwoffii	198	6E-50
ISAba46	IS66		Acinetobacter baumannii	198	6E-50
ISAba16	IS66		Acinetobacter baumannii	192	4E-48
ISAlw16	IS66		Acinetobacter lwoffii	190	2E-47
ISAba25	IS66		Acinetobacter baumannii	167	2E-40
ISAjo2	ISNCY	IS1202	Acinetobacter johnsonii	151	1E-35
ISAba49	IS66		Acinetobacter baumannii	121	1E-26
ISAba17	IS66		Acinetobacter baumannii	58.0	0,0000002
ISSod17	IS3	IS51	Shewanella oneidensis	52.0	0,000009

Query= MT-2_S8_L001_R1_001_(paired)_trimmed_(paired)_contig_55
Length=4589

Sequences producing significant alignments	IS Family	Group	Origin	Score (bits)	E. value
TnAs3	Tn3		Aeromonas salmonicida	2575	0.0
TnAs2	Tn3		Aeromonas salmonicida	270	2E-71
ISShes11	Tn3		Shewanella sp.	182	3E-45
IS15DI	IS6		Salmonella panama	119	4E-26
IS15	IS6		Salmonella panama	119	4E-26
IS26	IS6		Proteus vulgaris	119	4E-26
IS15DIV	IS6		Salmonella typhimurium	117	2E-25
IS15DII	IS6		Salmonella panama	111	1E-23

Query= MT-2_S8_L001_R1_001_(paired)_trimmed_(paired)_contig_57
Length=4546

Sequences producing significant alignments	IS Family	Group	Origin	Score (bits)	E. value
ISPpu12	ISL3		Pseudomonas putida	131	1E-29
IS1396	ISL3		Serratia marcescens	131	1E-29
TnAs3	Tn3		Aeromonas salmonicida	119	4E-26
ISPst2	ISL3		Pseudomonas stutzeri	85.7	6E-16
ISPst9	ISL3		Pseudomonas stutzeri	75.8	5E-13

Query= MT-2_S8_L001_R1_001_(paired)_trimmed_(paired)_contig_58
Length=4354

Sequences producing significant alignments	IS Family	Group	Origin	Score (bits)	E. value
ISAba14	IS3	IS150	Acinetobacter baumannii	605	2E-172
ISCfr1	IS1182		Citrobacter freundii	577	6E-164
ISPsp5	IS1182		Pseudomonas sp.	454	6E-127
ISAlw27	IS3	IS150	Acinetobacter lwoffii	383	2E-105
ISPa94	IS1182		Pseudomonas aeruginosa	309	2E-83
IS15DI	IS6		Salmonella panama	119	4E-26
IS15	IS6		Salmonella panama	119	4E-26
IS26	IS6		Proteus vulgaris	119	4E-26
ISAba52	IS6		Acinetobacter baumannii	117	2E-25
IS1008	IS6		Acinetobacter calcoaceticus	117	2E-25
ISOur1	IS6		Oligella urethralis	117	2E-25
IS15DIV	IS6		Salmonella typhimurium	117	2E-25
IS1007	IS6		Acinetobacter sp.	117	2E-25
IS15DII	IS6		Salmonella panama	111	1E-23
ISAcsp5	IS3	IS150	Acinetobacter sp.	58.0	0,0000001

Query= MT-2_S8_L001_R1_001_(paired)_trimmed_(paired)_contig_59
Length=4029

Sequences producing significant alignments	IS Family	Group	Origin	Score (bits)	E. value
IS15DI	IS6		Salmonella panama	1626	0.0
IS15	IS6		Salmonella panama	1626	0.0
IS15DIV	IS6		Salmonella typhimurium	1608	0.0
IS26	IS6		Proteus vulgaris	1602	0.0
IS15DII	IS6		Salmonella panama	1594	0.0
ISAjo2	ISNCY	IS1202	Acinetobacter johnsonii	400	7E-111
IS1006	IS6		Acinetobacter junii	180	1E-44
ISAba52	IS6		Acinetobacter baumannii	137	2E-31
IS1007	IS6		Acinetobacter sp.	137	2E-31
ISOur1	IS6		Oligella urethralis	121	9E-27
IS1008	IS6		Acinetobacter calcoaceticus	113	2E-24
ISStag1	IS6		Stappia aggregata	65.9	5E-10
ISAcr2	IS6		Acidiphilium cryptum	58.0	0,0000001
ISAba32	ISNCY	IS1202	Acinetobacter baumannii	50.1	0,00003

Query= MT-2_S8_L001_R1_001_(paired)_trimmed_(paired)_contig_60
Length=3979

Sequences producing significant alignments	IS Family	Group	Origin	Score (bits)	E. value
ISAba1	IS4	IS10	Acinetobacter baumannii	248	6E-65
ISAba33	IS4	IS10	Acinetobacter baumannii	163	3E-39
ISAba125	IS30		Acinetobacter baumannii	119	4E-26
ISC1041	IS30		Sulfolobus solfataricus	103	2E-21

Query= MT-2_S8_L001_R1_001_(paired)_trimmed_(paired)_contig_61
Length=3953

Sequences producing significant alignments	IS Family	Group	Origin	Score (bits)	E. value
ISAlw4	IS3	IS51	Acinetobacter lwoffii	1875	0.0
ISAba2	IS3	IS51	Acinetobacter baumannii	1574	0.0
ISAlw23	IS3	IS51	Acinetobacter lwoffii	1538	0.0
ISAba19	IS3	IS51	Acinetobacter baumannii	1463	0.0
ISAlw5	IS3	IS51	Acinetobacter lwoffii	1439	0.0
ISAba29	IS3	IS51	Acinetobacter baumannii	1352	0.0
ISAba34	IS3	IS51	Acinetobacter baumannii	1308	0.0
ISAba18	IS3	IS51	Acinetobacter baumannii	1304	0.0
IS1008	IS6		Acinetobacter calcoaceticus	504	6E-142
ISOur1	IS6		Oligella urethralis	448	3E-125
ISAba52	IS6		Acinetobacter baumannii	432	2E-120
IS1006	IS6		Acinetobacter junii	424	4E-118
IS1007	IS6		Acinetobacter sp.	129	4E-29
IS15DIV	IS6		Salmonella typhimurium	95.6	5E-19
IS15DII	IS6		Salmonella panama	95.6	5E-19
IS15DI	IS6		Salmonella panama	95.6	5E-19
IS15	IS6		Salmonella panama	95.6	5E-19
IS26	IS6		Proteus vulgaris	95.6	5E-19
IS1489v1	ISL3		Pseudomonas putida	83.8	2E-15
ISPpu33	IS3	IS51	Pseudomonas putida	56.0	0,0000004
ISStma17	IS3	IS51	Stenotrophomonas maltophilia	56.0	0,0000004
ISMca3	IS3	IS51	Methylococcus capsulatus	56.0	0,0000004
IS1416	IS3	IS51	Burkholderia glumae	56.0	0,0000004
ISAli4	IS3	IS51	Azospirillum lipoferum	54.0	0,000002
ISRme15	IS3	IS51	Ralstonia metallidurans	54.0	0,000002
ISPmi1	IS3	IS51	Proteus mirabilis	52.0	0,000007
ISRtr2	IS3	IS51	Rhizobium tropici	52.0	0,000007

Query= MT-2_S8_L001_R1_001_(paired)_trimmed_(paired)_contig_62
Length=3688

Sequences producing significant alignments	IS Family	Group	Origin	Score (bits)	E. value
Tn5393	Tn3		Erwinia amylovora	1358	0.0
ISEc63	Tn3		Escherichia coli	307	7E-83
Tn3	Tn3		Salmonella enterica	121	8E-27
IS15DIV	IS6		Salmonella typhimurium	119	3E-26
IS15DII	IS6		Salmonella panama	119	3E-26
IS15DI	IS6		Salmonella panama	119	3E-26
IS15	IS6		Salmonella panama	119	3E-26
IS26	IS6		Proteus vulgaris	119	3E-26
Tn2	Tn3		Escherichia coli	105	5E-22
ISMex22	Tn3		Methylobacterium extorquens	54.0	0,000002

Query= MT-2_S8_L001_R1_001_(paired)_trimmed_(paired)_contig_63
Length=3459

Sequences producing significant alignments	IS Family	Group	Origin	Score (bits)	E. value
ISAjo2	ISNCY	IS1202	Acinetobacter johnsonii	186	2E-46

Query= MT-2_S8_L001_R1_001_(paired)_trimmed_(paired)_contig_64
Length=3225

Sequences producing significant alignments	IS Family	Group	Origin	Score (bits)	E. value
ISPpu12	ISL3		Pseudomonas putida	5513	0.0
IS1396	ISL3		Serratia marcescens	2591	0.0
ISPst2	ISL3		Pseudomonas stutzeri	2313	0.0
ISPst9	ISL3		Pseudomonas stutzeri	1620	0.0

Query= MT-2_S8_L001_R1_001_(paired)_trimmed_(paired)_contig_65
Length=2804

Sequences producing significant alignments	IS Family	Group	Origin	Score (bits)	E. value
IS1008	IS6		Acinetobacter calcoaceticus	519	7E-147
ISOur1	IS6		Oligella urethralis	464	4E-130
ISAba52	IS6		Acinetobacter baumannii	448	2E-125
IS1006	IS6		Acinetobacter junii	440	5E-123
IS1007	IS6		Acinetobacter sp.	145	4E-34
IS15DIV	IS6		Salmonella typhimurium	87.7	9E-17
IS15DII	IS6		Salmonella panama	87.7	9E-17
IS15DI	IS6		Salmonella panama	87.7	9E-17
IS15	IS6		Salmonella panama	87.7	9E-17
IS26	IS6		Proteus vulgaris	87.7	9E-17
IS1489v1	ISL3		Pseudomonas putida	83.8	1E-15

Query= MT-2_S8_L001_R1_001_(paired)_trimmed_(paired)_contig_66
Length=2667

Sequences producing significant alignments	IS Family	Group	Origin	Score (bits)	E. value
ISAba1	IS4	IS10	Acinetobacter baumannii	212	2E-54
ISAba33	IS4	IS10	Acinetobacter baumannii	135	4E-31

Query= MT-2_S8_L001_R1_001_(paired)_trimmed_(paired)_contig_68

Length=2286

Sequences producing significant alignments	*IS Family*	*Group*	*Origin*	*Score (bits)*	*E. value*
ISPpu12	*ISL3*		*Pseudomonas putida*	361	3E-99
TnAs3	*Tn3*		*Aeromonas salmonicida*	119	2E-26
ISXc4	*Tn3*		*Xanthomonas campestris*	73.8	1E-12
ISPsy42	*Tn3*		*Pseudomonas syringae*	63.9	0,000000001
ISThsp9	*Tn3*		*Thiomonas sp.*	54.0	0,000001
ISMex22	*Tn3*		*Methylobacterium extorquens*	52.0	0,000004

Query= MT-2_S8_L001_R1_001_(paired)_trimmed_(paired)_contig_71
Length=1597

Sequences producing significant alignments	*IS Family*	*Group*	*Origin*	*Score (bits)*	*E. value*
TnAs3	*Tn3*		*Aeromonas salmonicida*	3166	0.0

Query= MT-2_S8_L001_R1_001_(paired)_trimmed_(paired)_contig_72
Length=1578

Sequences producing significant alignments	*IS Family*	*Group*	*Origin*	*Score (bits)*	*E. value*
TnAs3	*Tn3*		*Aeromonas salmonicida*	860	0.0
ISAba125	*IS30*		*Acinetobacter baumannii*	448	1E-125
ISC1041	*IS30*		*Sulfolobus solfataricus*	343	5E-94
IS15DIV	*IS6*		*Salmonella typhimurium*	119	1E-26
IS15DII	*IS6*		*Salmonella panama*	119	1E-26
IS15DI	*IS6*		*Salmonella panama*	119	1E-26
IS15	*IS6*		*Salmonella panama*	119	1E-26
IS26	*IS6*		*Proteus vulgaris*	119	1E-26
ISAbe8	*IS30*		*Acinetobacter bereziniae*	58.0	0,00000004

Query= MT-2_S8_L001_R1_001_(paired)_trimmed_(paired)_contig_73
Length=1549

Sequences producing significant alignments	*IS Family*	*Group*	*Origin*	*Score (bits)*	*E. value*
IS15	*IS6*		*Salmonella panama*	466	5E-131
IS15DII	*IS6*		*Salmonella panama*	347	3E-95
IS15DI	*IS6*		*Salmonella panama*	339	8E-93
IS26	*IS6*		*Proteus vulgaris*	339	8E-93
IS15DIV	*IS6*		*Salmonella typhimurium*	337	3E-92
ISAba52	*IS6*		*Acinetobacter baumannii*	61.9	0,000000003
IS1008	*IS6*		*Acinetobacter calcoaceticus*	61.9	0,000000003
ISOur1	*IS6*		*Oligella urethralis*	61.9	0,000000003
IS1006	*IS6*		*Acinetobacter junii*	61.9	0,000000003
IS1007	*IS6*		*Acinetobacter sp.*	52.0	0,000003

Query= MT-2_S8_L001_R1_001_(paired)_trimmed_(paired)_contig_75
Length=1038

Sequences producing significant alignments	*IS Family*	*Group*	*Origin*	*Score (bits)*	*E. value*
ISAba13	*IS5*	*IS903*	*Acinetobacter baumannii*	2058	0.0
ISAba12	*IS5*	*IS903*	*Acinetobacter baumannii*	848	0.0
IS17	*IS5*	*IS903*	*Acinetobacter haemolyticus*	835	0.0
ISAba40	*IS5*	*IS903*	*Acinetobacter baumannii*	803	0.0
ISAha1	*IS5*	*IS903*	*Acinetobacter haemolyticus*	801	0.0
ISAlw1	*IS5*	*IS903*	*Acinetobacter lwoffii*	702	0.0
ISAjo1	*IS5*	*IS903*	*Acinetobacter johnsonii*	634	0.0
ISAba53	*IS5*	*IS903*	*Acinetobacter baumannii*	617	2E-176
ISAha3	*IS5*	*IS903*	*Acinetobacter haemolyticus*	517	1E-146
ISAha2	*IS5*	*IS903*	*Acinetobacter haemolyticus*	428	7E-120
ISAba7	*IS5*	*IS903*	*Acinetobacter baumannii*	420	2E-117
ISAba5	*IS5*	*IS903*	*Acinetobacter baumannii*	387	3E-107

Query= MT-2_S8_L001_R1_001_(paired)_trimmed_(paired)_contig_76
Length=1030

Sequences producing significant alignments	*IS Family*	*Group*	*Origin*	*Score (bits)*	*E. value*
ISAba125	*IS30*		*Acinetobacter baumannii*	1810	0.0
ISC1041	*IS30*		*Sulfolobus solfataricus*	668	0.0
ISAbe8	*IS30*		*Acinetobacter bereziniae*	317	2E-86

Query= MT-2_S8_L001_R1_001_(paired)_trimmed_(paired)_contig_77
Length=905

Sequences producing significant alignments	*IS Family*	*Group*	*Origin*	*Score (bits)*	*E. value*
ISAba125	*IS30*		*Acinetobacter baumannii*	119	8E-27
ISC1041	*IS30*		*Sulfolobus solfataricus*	103	5E-22

Query= MT-2_S8_L001_R1_001_(paired)_trimmed_(paired)_contig_78
Length=705

Sequences producing significant alignments	*IS Family*	*Group*	*Origin*	*Score (bits)*	*E. value*
ISAjo2	*ISNCY*	*IS1202*	*Acinetobacter johnsonii*	894	0.0
ISAba32	*ISNCY*	*IS1202*	*Acinetobacter baumannii*	159	7E-39
ISAlw22	*ISNCY*	*IS1202*	*Acinetobacter lwoffii*	145	1E-34
ISEsa1	*ISNCY*	*IS1202*	*Enterobacter sakazakii*	65.9	8E-11
ISLad2	*ISNCY*	*IS1202*	*Leclercia adecarboxylata*	65.9	8E-11

Query= MT-2_S8_L001_R1_001_(paired)_trimmed_(paired)_contig_80
Length=619

Sequences producing significant alignments	*IS Family*	*Group*	*Origin*	*Score (bits)*	*E. value*
IS1008	*IS6*		*Acinetobacter calcoaceticus*	1209	0.0
ISOur1	*IS6*		*Oligella urethralis*	1162	0.0
ISAba52	*IS6*		*Acinetobacter baumannii*	662	0.0
IS1007	*IS6*		*Acinetobacter sp.*	525	3E-149
IS1006	*IS6*		*Acinetobacter junii*	502	4E-142
IS15DIV	*IS6*		*Salmonella typhimurium*	113	3E-25

IS15DII	*IS6*		*Salmonella panama*	113	3E-25
IS15DI	*IS6*		*Salmonella panama*	113	3E-25
IS15	*IS6*		*Salmonella panama*	113	3E-25
IS26	*IS6*		*Proteus vulgaris*	113	3E-25

Query= MT-2_S8_L001_R1_001_(paired)_trimmed_(paired)_contig_84
Length=550

Sequences producing significant alignments	*IS Family*	*Group*	*Origin*	*Score (bits)*	*E. value*
ISAba125	*IS30*		*Acinetobacter baumannii*	119	5E-27
ISC1041	*IS30*		*Sulfolobus solfataricus*	103	3E-22
ISAbe8	*IS30*		*Acinetobacter bereziniae*	46.1	0,00006

Query= MT-2_S8_L001_R1_001_(paired)_trimmed_(paired)_contig_86
Length=451

Sequences producing significant alignments	*IS Family*	*Group*	*Origin*	*Score (bits)*	*E. value*
ISAba125	*IS30*		*Acinetobacter baumannii*	878	0.0
ISC1041	*IS30*		*Sulfolobus solfataricus*	581	4E-166
ISAbe8	*IS30*		*Acinetobacter bereziniae*	196	2E-50

Query= MT-2_S8_L001_R1_001_(paired)_trimmed_(paired)_contig_87
Length=441

Sequences producing significant alignments	*IS Family*	*Group*	*Origin*	*Score (bits)*	*E. value*
ISAjo2	*ISNCY*	*IS1202*	*Acinetobacter johnsonii*	708	0.0
ISAlw22	*ISNCY*	*IS1202*	*Acinetobacter lwoffii*	117	2E-26
ISAba32	*ISNCY*	*IS1202*	*Acinetobacter baumannii*	111	9E-25
ISEsa1	*ISNCY*	*IS1202*	*Enterobacter sakazakii*	65.9	5E-11
ISLad2	*ISNCY*	*IS1202*	*Leclercia adecarboxylata*	65.9	5E-11

Query= MT-2_S8_L001_R1_001_(paired)_trimmed_(paired)_contig_88
Length=405

Sequences producing significant alignments	*IS Family*	*Group*	*Origin*	*Score (bits)*	*E. value*
ISAba1	*IS4*	*IS10*	*Acinetobacter baumannii*	248	6E-66
ISAba33	*IS4*	*IS10*	*Acinetobacter baumannii*	163	3E-40

Query= MT-2_S8_L001_R1_001_(paired)_trimmed_(paired)_contig_91
Length=392

Sequences producing significant alignments	*IS Family*	*Group*	*Origin*	*Score (bits)*	*E. value*
ISAjo2	*ISNCY*	*IS1202*	*Acinetobacter johnsonii*	422	2E-118
ISAlw22	*ISNCY*	*IS1202*	*Acinetobacter lwoffii*	61.9	7E-10
ISAba32	*ISNCY*	*IS1202*	*Acinetobacter baumannii*	61.9	7E-10

Query= MT-2_S8_L001_R1_001_(paired)_trimmed_(paired)_contig_92
Length=315

Sequences producing significant alignments	*IS Family*	*Group*	*Origin*	*Score (bits)*	*E. value*
ISAjo2	*ISNCY*	*IS1202*	*Acinetobacter johnsonii*	509	7E-145
ISAlw22	*ISNCY*	*IS1202*	*Acinetobacter lwoffii*	135	5E-32
ISAba32	*ISNCY*	*IS1202*	*Acinetobacter baumannii*	105	4E-23
ISLad2	*ISNCY*	*IS1202*	*Leclercia adecarboxylata*	50.1	0,000002

Query= MT-2_S8_L001_R1_001_(paired)_trimmed_(paired)_contig_93
Length=309

Sequences producing significant alignments	*IS Family*	*Group*	*Origin*	*Score (bits)*	*E. value*
ISAjo2	*ISNCY*	*IS1202*	*Acinetobacter johnsonii*	391	5E-109
ISAba32	*ISNCY*	*IS1202*	*Acinetobacter baumannii*	63.9	1E-10
ISAlw22	*ISNCY*	*IS1202*	*Acinetobacter lwoffii*	61.9	5E-10

Query= MT-2_S8_L001_R1_001_(paired)_trimmed_(paired)_contig_94
Length=309

Sequences producing significant alignments	*IS Family*	*Group*	*Origin*	*Score (bits)*	*E. value*
ISAjo2	*ISNCY*	*IS1202*	*Acinetobacter johnsonii*	391	5E-109
ISAlw22	*ISNCY*	*IS1202*	*Acinetobacter lwoffii*	56.0	0,00000003
ISAba32	*ISNCY*	*IS1202*	*Acinetobacter baumannii*	56.0	0,00000003

Query= MT-2_S8_L001_R1_001_(paired)_trimmed_(paired)_contig_95
Length=309

Sequences producing significant alignments	*IS Family*	*Group*	*Origin*	*Score (bits)*	*E. value*
ISAjo2	*ISNCY*	*IS1202*	*Acinetobacter johnsonii*	391	5E-109
ISAlw22	*ISNCY*	*IS1202*	*Acinetobacter lwoffii*	56.0	0,00000003
ISAba32	*ISNCY*	*IS1202*	*Acinetobacter baumannii*	56.0	0,00000003

Query= MT-2_S8_L001_R1_001_(paired)_trimmed_(paired)_contig_96
Length=309

Sequences producing significant alignments	*IS Family*	*Group*	*Origin*	*Score (bits)*	*E. value*
ISAjo2	*ISNCY*	*IS1202*	*Acinetobacter johnsonii*	383	1E-106
ISAba32	*ISNCY*	*IS1202*	*Acinetobacter baumannii*	56.0	0,00000003
ISAlw22	*ISNCY*	*IS1202*	*Acinetobacter lwoffii*	54.0	0,0000001

Query= MT-2_S8_L001_R1_001_(paired)_trimmed_(paired)_contig_97
Length=309

Sequences producing significant alignments	*IS Family*	*Group*	*Origin*	*Score (bits)*	*E. value*
ISAjo2	*ISNCY*	*IS1202*	*Acinetobacter johnsonii*	391	5E-109

Sequences producing significant alignments	IS Family	Group	Origin	Score (bits)	E. value
ISAlw22	ISNCY	IS1202	*Acinetobacter lwoffii*	61.9	5E-10
ISAba32	ISNCY	IS1202	*Acinetobacter baumannii*	61.9	5E-10

Query= MT-2_S8_L001_R1_001_(paired)_trimmed_(paired)_contig_98
Length=309

Sequences producing significant alignments	IS Family	Group	Origin	Score (bits)	E. value
ISAjo2	ISNCY	IS1202	*Acinetobacter johnsonii*	391	5E-109
ISAba32	ISNCY	IS1202	*Acinetobacter baumannii*	56.0	0,00000003
ISAlw22	ISNCY	IS1202	*Acinetobacter lwoffii*	54.0	0,0000001

Query= MT-2_S8_L001_R1_001_(paired)_trimmed_(paired)_contig_99
Length=309

Sequences producing significant alignments	IS Family	Group	Origin	Score (bits)	E. value
ISAjo2	ISNCY	IS1202	*Acinetobacter johnsonii*	391	5E-109
ISAba32	ISNCY	IS1202	*Acinetobacter baumannii*	63.9	1E-10
ISAlw22	ISNCY	IS1202	*Acinetobacter lwoffii*	61.9	5E-10

Query= MT-2_S8_L001_R1_001_(paired)_trimmed_(paired)_contig_100
Length=309

Sequences producing significant alignments	IS Family	Group	Origin	Score (bits)	E. value
ISAjo2	ISNCY	IS1202	*Acinetobacter johnsonii*	391	5E-109
ISAlw22	ISNCY	IS1202	*Acinetobacter lwoffii*	61.9	5E-10
ISAba32	ISNCY	IS1202	*Acinetobacter baumannii*	61.9	5E-10

Query= MT-2_S8_L001_R1_001_(paired)_trimmed_(paired)_contig_102
Length=306

Sequences producing significant alignments	IS Family	Group	Origin	Score (bits)	E. value
ISAjo2	ISNCY	IS1202	*Acinetobacter johnsonii*	367	7E-102
ISAlw22	ISNCY	IS1202	*Acinetobacter lwoffii*	117	1E-26
ISAba32	ISNCY	IS1202	*Acinetobacter baumannii*	99.6	2E-21
ISEsa1	ISNCY	IS1202	*Enterobacter sakazakii*	65.9	3E-11
ISLad2	ISNCY	IS1202	*Leclercia adecarboxylata*	65.9	3E-11

Query= MT-2_S8_L001_R1_001_(paired)_trimmed_(paired)_contig_103
Length=298

Sequences producing significant alignments	IS Family	Group	Origin	Score (bits)	E. value
ISAjo2	ISNCY	IS1202	*Acinetobacter johnsonii*	361	4E-100
ISAlw22	ISNCY	IS1202	*Acinetobacter lwoffii*	54.0	0,0000001
ISAba32	ISNCY	IS1202	*Acinetobacter baumannii*	54.0	0,0000001

Query= MT-2_S8_L001_R1_001_(paired)_trimmed_(paired)_contig_105
Length=290

Sequences producing significant alignments	IS Family	Group	Origin	Score (bits)	E. value
ISAjo2	ISNCY	IS1202	*Acinetobacter johnsonii*	392	1E-109
ISAlw22	ISNCY	IS1202	*Acinetobacter lwoffii*	117	1E-26
ISAba32	ISNCY	IS1202	*Acinetobacter baumannii*	99.6	2E-21
ISEsa1	ISNCY	IS1202	*Enterobacter sakazakii*	65.9	3E-11
ISLad2	ISNCY	IS1202	*Leclercia adecarboxylata*	65.9	3E-11

Query= MT-2_S8_L001_R1_001_(paired)_trimmed_(paired)_contig_106
Length=284

Sequences producing significant alignments	IS Family	Group	Origin	Score (bits)	E. value
ISAjo2	ISNCY	IS1202	*Acinetobacter johnsonii*	349	1E-96
ISAba32	ISNCY	IS1202	*Acinetobacter baumannii*	71.9	5E-13

Query= MT-2_S8_L001_R1_001_(paired)_trimmed_(paired)_contig_107
Length=284

Sequences producing significant alignments	IS Family	Group	Origin	Score (bits)	E. value
ISAjo2	ISNCY	IS1202	*Acinetobacter johnsonii*	349	1E-96
ISAba32	ISNCY	IS1202	*Acinetobacter baumannii*	71.9	5E-13

Query= MT-2_S8_L001_R1_001_(paired)_trimmed_(paired)_contig_108
Length=284

Sequences producing significant alignments	IS Family	Group	Origin	Score (bits)	E. value
ISAjo2	ISNCY	IS1202	*Acinetobacter johnsonii*	349	1E-96
ISAba32	ISNCY	IS1202	*Acinetobacter baumannii*	71.9	5E-13

Query= MT-2_S8_L001_R1_001_(paired)_trimmed_(paired)_contig_109
Length=283

Sequences producing significant alignments	IS Family	Group	Origin	Score (bits)	E. value
ISAjo2	ISNCY	IS1202	*Acinetobacter johnsonii*	333	9E-92
ISAba32	ISNCY	IS1202	*Acinetobacter baumannii*	71.9	5E-13

Query= MT-2_S8_L001_R1_001_(paired)_trimmed_(paired)_contig_110
Length=282

Sequences producing significant alignments	IS Family	Group	Origin	Score (bits)	E. value
ISAjo2	ISNCY	IS1202	*Acinetobacter johnsonii*	295	2E-80
ISAba32	ISNCY	IS1202	*Acinetobacter baumannii*	63.9	1E-10
ISAlw22	ISNCY	IS1202	*Acinetobacter lwoffii*	61.9	5E-10

Query= MT-2_S8_L001_R1_001_(paired)_trimmed_(paired)_contig_113
Length=270

Sequences producing significant alignments	IS Family	Group	Origin	Score (bits)	E. value
ISAjo2	ISNCY	IS1202	*Acinetobacter johnsonii*	470	5E-133
ISAlw22	ISNCY	IS1202	*Acinetobacter lwoffii*	111	6E-25
ISAba32	ISNCY	IS1202	*Acinetobacter baumannii*	111	6E-25
ISLad2	ISNCY	IS1202	*Leclercia adecarboxylata*	56.0	0,00000003
ISRel10	ISNCY	IS1202	*Rhizobium etli*	46.1	0,00003

Query= MT-2_S8_L001_R1_001_(paired)_trimmed_(paired)_contig_114
Length=269

Sequences producing significant alignments	IS Family	Group	Origin	Score (bits)	E. value
ISAjo2	ISNCY	IS1202	*Acinetobacter johnsonii*	329	1E-90
ISAba32	ISNCY	IS1202	*Acinetobacter baumannii*	58.0	0,000000007
ISAlw22	ISNCY	IS1202	*Acinetobacter lwoffii*	56.0	0,00000003

Query= MT-2_S8_L001_R1_001_(paired)_trimmed_(paired)_contig_115
Length=268

Sequences producing significant alignments	IS Family	Group	Origin	Score (bits)	E. value
ISAjo2	ISNCY	IS1202	*Acinetobacter johnsonii*	278	4E-75
ISAba32	ISNCY	IS1202	*Acinetobacter baumannii*	56.0	0,00000003
ISAlw22	ISNCY	IS1202	*Acinetobacter lwoffii*	54.0	0,0000001

Query= MT-2_S8_L001_R1_001_(paired)_trimmed_(paired)_contig_116
Length=266

Sequences producing significant alignments	IS Family	Group	Origin	Score (bits)	E. value
ISAjo2	ISNCY	IS1202	*Acinetobacter johnsonii*	311	3E-85
ISAlw22	ISNCY	IS1202	*Acinetobacter lwoffii*	117	9E-27
ISAba32	ISNCY	IS1202	*Acinetobacter baumannii*	101	5E-22
ISEsa1	ISNCY	IS1202	*Enterobacter sakazakii*	65.9	3E-11
ISLad2	ISNCY	IS1202	*Leclercia adecarboxylata*	65.9	3E-11

Query= MT-2_S8_L001_R1_001_(paired)_trimmed_(paired)_contig_117
Length=265

Sequences producing significant alignments	IS Family	Group	Origin	Score (bits)	E. value
ISAjo2	ISNCY	IS1202	*Acinetobacter johnsonii*	303	7E-83
ISAlw22	ISNCY	IS1202	*Acinetobacter lwoffii*	61.9	5E-10
ISAba32	ISNCY	IS1202	*Acinetobacter baumannii*	61.9	5E-10

Query= MT-2_S8_L001_R1_001_(paired)_trimmed_(paired)_contig_118
Length=258

Sequences producing significant alignments	IS Family	Group	Origin	Score (bits)	E. value
ISAjo2	ISNCY	IS1202	*Acinetobacter johnsonii*	337	5E-93
ISAlw22	ISNCY	IS1202	*Acinetobacter lwoffii*	117	9E-27
ISAba32	ISNCY	IS1202	*Acinetobacter baumannii*	101	5E-22
ISEsa1	ISNCY	IS1202	*Enterobacter sakazakii*	65.9	3E-11
ISLad2	ISNCY	IS1202	*Leclercia adecarboxylata*	65.9	3E-11

Query= MT-2_S8_L001_R1_001_(paired)_trimmed_(paired)_contig_119
Length=248

Sequences producing significant alignments	IS Family	Group	Origin	Score (bits)	E. value
ISAjo2	ISNCY	IS1202	*Acinetobacter johnsonii*	317	4E-87
ISAlw22	ISNCY	IS1202	*Acinetobacter lwoffii*	117	8E-27
ISAba32	ISNCY	IS1202	*Acinetobacter baumannii*	101	5E-22
ISEsa1	ISNCY	IS1202	*Enterobacter sakazakii*	65.9	3E-11
ISLad2	ISNCY	IS1202	*Leclercia adecarboxylata*	65.9	3E-11

Query= MT-2_S8_L001_R1_001_(paired)_trimmed_(paired)_contig_121
Length=246

Sequences producing significant alignments	IS Family	Group	Origin	Score (bits)	E. value
ISAjo2	ISNCY	IS1202	*Acinetobacter johnsonii*	270	9E-73
ISAlw22	ISNCY	IS1202	*Acinetobacter lwoffii*	61.9	4E-10
ISAba32	ISNCY	IS1202	*Acinetobacter baumannii*	61.9	4E-10

Query= MT-2_S8_L001_R1_001_(paired)_trimmed_(paired)_contig_122
Length=242

Sequences producing significant alignments	IS Family	Group	Origin	Score (bits)	E. value
ISAjo2	ISNCY	IS1202	*Acinetobacter johnsonii*	305	2E-83
ISAlw22	ISNCY	IS1202	*Acinetobacter lwoffii*	117	8E-27
ISAba32	ISNCY	IS1202	*Acinetobacter baumannii*	99.6	2E-21
ISEsa1	ISNCY	IS1202	*Enterobacter sakazakii*	65.9	3E-11
ISLad2	ISNCY	IS1202	*Leclercia adecarboxylata*	65.9	3E-11

Query= MT-2_S8_L001_R1_001_(paired)_trimmed_(paired)_contig_126
Length=238

Sequences producing significant alignments	IS Family	Group	Origin	Score (bits)	E. value
ISAjo2	ISNCY	IS1202	*Acinetobacter johnsonii*	297	4E-81
ISAba32	ISNCY	IS1202	*Acinetobacter baumannii*	60.0	0,000000002
ISAlw22	ISNCY	IS1202	*Acinetobacter lwoffii*	58.0	0,000000006

Query= MT-2_S8_L001_R1_001_(paired)_trimmed_(paired)_contig_127
Length=237

Sequences producing significant alignments	IS Family	Group	Origin	Score (bits)	E. value
ISAjo2	*ISNCY*	*IS1202*	*Acinetobacter johnsonii*	287	4E-78
ISAlw22	*ISNCY*	*IS1202*	*Acinetobacter lwoffii*	61.9	4E-10
ISAba32	*ISNCY*	*IS1202*	*Acinetobacter baumannii*	61.9	4E-10

Query= MT-2_S8_L001_R1_001_(paired)_trimmed_(paired)_contig_128
Length=236

Sequences producing significant alignments	IS Family	Group	Origin	Score (bits)	E. value
ISAjo2	*ISNCY*	*IS1202*	*Acinetobacter johnsonii*	428	2E-120
ISAlw22	*ISNCY*	*IS1202*	*Acinetobacter lwoffii*	111	5E-25
ISAba32	*ISNCY*	*IS1202*	*Acinetobacter baumannii*	111	5E-25
ISLad2	*ISNCY*	*IS1202*	*Leclercia adecarboxylata*	56.0	0,00000002
ISRel10	*ISNCY*	*IS1202*	*Rhizobium etli*	46.1	0,00002

Query= MT-2_S8_L001_R1_001_(paired)_trimmed_(paired)_contig_129
Length=234

Sequences producing significant alignments	IS Family	Group	Origin	Score (bits)	E. value
ISAjo2	*ISNCY*	*IS1202*	*Acinetobacter johnsonii*	289	9E-79
ISAba32	*ISNCY*	*IS1202*	*Acinetobacter baumannii*	63.9	1E-10
ISAlw22	*ISNCY*	*IS1202*	*Acinetobacter lwoffii*	61.9	4E-10

Query= MT-2_S8_L001_R1_001_(paired)_trimmed_(paired)_contig_130
Length=233

Sequences producing significant alignments	IS Family	Group	Origin	Score (bits)	E. value
ISAjo2	*ISNCY*	*IS1202*	*Acinetobacter johnsonii*	276	1E-74
ISAlw22	*ISNCY*	*IS1202*	*Acinetobacter lwoffii*	61.9	4E-10
ISAba32	*ISNCY*	*IS1202*	*Acinetobacter baumannii*	61.9	4E-10

Query= MT-2_S8_L001_R1_001_(paired)_trimmed_(paired)_contig_131
Length=231

Sequences producing significant alignments	IS Family	Group	Origin	Score (bits)	E. value
ISAjo2	*ISNCY*	*IS1202*	*Acinetobacter johnsonii*	276	1E-74
ISAba32	*ISNCY*	*IS1202*	*Acinetobacter baumannii*	115	3E-26
ISAlw22	*ISNCY*	*IS1202*	*Acinetobacter lwoffii*	91.7	4E-19
ISEsa1	*ISNCY*	*IS1202*	*Enterobacter sakazakii*	50.1	0,000001

Query= MT-2_S8_L001_R1_001_(paired)_trimmed_(paired)_contig_132
Length=231

Sequences producing significant alignments	IS Family	Group	Origin	Score (bits)	E. value
ISAjo2	*ISNCY*	*IS1202*	*Acinetobacter johnsonii*	283	6E-77

Query= MT-2_S8_L001_R1_001_(paired)_trimmed_(paired)_contig_133
Length=231

Sequences producing significant alignments	IS Family	Group	Origin	Score (bits)	E. value
ISAjo2	*ISNCY*	*IS1202*	*Acinetobacter johnsonii*	339	1E-93

Query= MT-2_S8_L001_R1_001_(paired)_trimmed_(paired)_contig_135
Length=230

Sequences producing significant alignments	IS Family	Group	Origin	Score (bits)	E. value
ISAba125	*IS30*		*Acinetobacter baumannii*	456	7E-129
ISC1041	*IS30*		*Sulfolobus solfataricus*	335	2E-92
ISAbe8	*IS30*		*Acinetobacter bereziniae*	58.0	0,000000006

Query= MT-2_S8_L001_R1_001_(paired)_trimmed_(paired)_contig_136
Length=228

Sequences producing significant alignments	IS Family	Group	Origin	Score (bits)	E. value
ISAjo2	*ISNCY*	*IS1202*	*Acinetobacter johnsonii*	335	2E-92
ISAlw22	*ISNCY*	*IS1202*	*Acinetobacter lwoffii*	111	5E-25
ISAba32	*ISNCY*	*IS1202*	*Acinetobacter baumannii*	111	5E-25
ISLad2	*ISNCY*	*IS1202*	*Leclercia adecarboxylata*	56.0	0,00000002
ISRel10	*ISNCY*	*IS1202*	*Rhizobium etli*	46.1	0,00002

Query= MT-2_S8_L001_R1_001_(paired)_trimmed_(paired)_contig_137
Length=227

Sequences producing significant alignments	IS Family	Group	Origin	Score (bits)	E. value
ISAlw22	*ISNCY*	*IS1202*	*Acinetobacter lwoffii*	194	4E-50
ISAba32	*ISNCY*	*IS1202*	*Acinetobacter baumannii*	178	2E-45
ISAjo2	*ISNCY*	*IS1202*	*Acinetobacter johnsonii*	75.8	3E-14
ISEsa1	*ISNCY*	*IS1202*	*Enterobacter sakazakii*	60.0	0,000000002

Query= MT-2_S8_L001_R1_001_(paired)_trimmed_(paired)_contig_139
Length=225

Sequences producing significant alignments	IS Family	Group	Origin	Score (bits)	E. value
ISAba1	*IS4*	*IS10*	*Acinetobacter baumannii*	414	2E-116
ISAba33	*IS4*	*IS10*	*Acinetobacter baumannii*	216	1E-56

Query= MT-2_S8_L001_R1_001_(paired)_trimmed_(paired)_contig_140
Length=223

Sequences producing significant alignments	IS Family	Group	Origin	Score (bits)	E. value
ISAjo2	*ISNCY*	*IS1202*	*Acinetobacter johnsonii*	414	2E-116
ISAlw22	*ISNCY*	*IS1202*	*Acinetobacter lwoffii*	111	5E-25
ISAba32	*ISNCY*	*IS1202*	*Acinetobacter baumannii*	111	5E-25
ISLad2	*ISNCY*	*IS1202*	*Leclercia adecarboxylata*	56.0	0,00000002

ISRel10	ISNCY	IS1202	*Rhizobium etli*	46.1	0,00002

Query= MT-2_S8_L001_R1_001_(paired)_trimmed_(paired)_contig_141
Length=222

Sequences producing significant alignments	IS Family	Group	Origin	Score (bits)	E. value
ISAjo2	ISNCY	IS1202	*Acinetobacter johnsonii*	206	1E-53
ISAlw22	ISNCY	IS1202	*Acinetobacter lwoffii*	117	7E-27
ISAba32	ISNCY	IS1202	*Acinetobacter baumannii*	99.6	2E-21
ISEsa1	ISNCY	IS1202	*Enterobacter sakazakii*	65.9	2E-11
ISLad2	ISNCY	IS1202	*Leclercia adecarboxylata*	65.9	2E-11

Query= MT-2_S8_L001_R1_001_(paired)_trimmed_(paired)_contig_142
Length=222

Sequences producing significant alignments	IS Family	Group	Origin	Score (bits)	E. value
ISAjo2	ISNCY	IS1202	*Acinetobacter johnsonii*	147	8E-36
ISAba32	ISNCY	IS1202	*Acinetobacter baumannii*	85.7	3E-17

Query= MT-2_S8_L001_R1_001_(paired)_trimmed_(paired)_contig_144
Length=221

Sequences producing significant alignments	IS Family	Group	Origin	Score (bits)	E. value
ISAjo2	ISNCY	IS1202	*Acinetobacter johnsonii*	327	4E-90
ISAba32	ISNCY	IS1202	*Acinetobacter baumannii*	50.1	0,000001

Query= MT-2_S8_L001_R1_001_(paired)_trimmed_(paired)_contig_147
Length=218

Sequences producing significant alignments	IS Family	Group	Origin	Score (bits)	E. value
ISAjo2	ISNCY	IS1202	*Acinetobacter johnsonii*	276	1E-74

Query= MT-2_S8_L001_R1_001_(paired)_trimmed_(paired)_contig_149
Length=217

Sequences producing significant alignments	IS Family	Group	Origin	Score (bits)	E. value
ISAba1	IS4	IS10	*Acinetobacter baumannii*	414	2E-11

Query= MT-2_S8_L001_R1_001_(paired)_trimmed_(paired)_contig_150
Length=215

Sequences producing significant alignments	IS Family	Group	Origin	Score (bits)	E. value
ISAjo2	ISNCY	IS1202	*Acinetobacter johnsonii*	295	1E-80
ISCARN112	ISNCY	IS1202	*Metagenomic data*	48.1	0,000006

Query= MT-2_S8_L001_R1_001_(paired)_trimmed_(paired)_contig_151
Length=215

Sequences producing significant alignments	IS Family	Group	Origin	Score (bits)	E. value
ISAjo2	ISNCY	IS1202	*Acinetobacter johnsonii*	295	1E-80

Query= MT-2_S8_L001_R1_001_(paired)_trimmed_(paired)_contig_152
Length=214

Sequences producing significant alignments	IS Family	Group	Origin	Score (bits)	E. value
ISAjo2	ISNCY	IS1202	*Acinetobacter johnsonii*	281	2E-76
ISAlw22	ISNCY	IS1202	*Acinetobacter lwoffii*	109	2E-24
ISAba32	ISNCY	IS1202	*Acinetobacter baumannii*	93.7	1E-19
ISEsa1	ISNCY	IS1202	*Enterobacter sakazakii*	58.0	0,000000006
ISLad2	ISNCY	IS1202	*Leclercia adecarboxylata*	58.0	0,000000006

Query= MT-2_S8_L001_R1_001_(paired)_trimmed_(paired)_contig_153
Length=213

Sequences producing significant alignments	IS Family	Group	Origin	Score (bits)	E. value
ISAjo2	ISNCY	IS1202	*Acinetobacter johnsonii*	335	2E-92

Query= MT-2_S8_L001_R1_001_(paired)_trimmed_(paired)_contig_155
Length=213

Sequences producing significant alignments	IS Family	Group	Origin	Score (bits)	E. value
ISAjo2	ISNCY	IS1202	*Acinetobacter johnsonii*	335	2E-92

Query= MT-2_S8_L001_R1_001_(paired)_trimmed_(paired)_contig_156
Length=213

Sequences producing significant alignments	IS Family	Group	Origin	Score (bits)	E. value
ISAjo2	ISNCY	IS1202	*Acinetobacter johnsonii*	335	2E-92

Query= MT-2_S8_L001_R1_001_(paired)_trimmed_(paired)_contig_157
Length=213

Sequences producing significant alignments	IS Family	Group	Origin	Score (bits)	E. value
ISAba1	IS4	IS10	*Acinetobacter baumannii*	406	5E-114
ISAba33	IS4	IS10	*Acinetobacter baumannii*	226	1E-59

Query= MT-2_S8_L001_R1_001_(paired)_trimmed_(paired)_contig_158
Length=213

Sequences producing significant alignments	IS Family	Group	Origin	Score (bits)	E. value
ISAjo2	ISNCY	IS1202	*Acinetobacter johnsonii*	151	5E-37
ISCARN112	ISNCY	IS1202	*Metagenomic data*	48.1	0,000005

Query= MT-2_S8_L001_R1_001_(paired)_trimmed_(paired)_contig_159
Length=213

Sequences producing significant alignments	IS Family	Group	Origin	Score (bits)	E. value
ISAjo2	ISNCY	IS1202	Acinetobacter johnsonii	343	7E-95

Query= MT-2_S8_L001_R1_001_(paired)_trimmed_(paired)_contig_160
Length=213

Sequences producing significant alignments	IS Family	Group	Origin	Score (bits)	E. value
ISAba1	IS4	IS10	Acinetobacter baumannii	414	2E-116
ISAba33	IS4	IS10	Acinetobacter baumannii	218	3E-57

Query= MT-2_S8_L001_R1_001_(paired)_trimmed_(paired)_contig_162
Length=212

Sequences producing significant alignments	IS Family	Group	Origin	Score (bits)	E. value
ISAjo2	ISNCY	IS1202	Acinetobacter johnsonii	319	1E-87

Query= MT-2_S8_L001_R1_001_(paired)_trimmed_(paired)_contig_164
Length=208

Sequences producing significant alignments	IS Family	Group	Origin	Score (bits)	E. value
ISAjo2	ISNCY	IS1202	Acinetobacter johnsonii	262	2E-70
ISAlw22	ISNCY	IS1202	Acinetobacter lwoffii	117	7E-27
ISAba32	ISNCY	IS1202	Acinetobacter baumannii	99.6	2E-21
ISEsa1	ISNCY	IS1202	Enterobacter sakazakii	65.9	2E-11
ISLad2	ISNCY	IS1202	Leclercia adecarboxylata	65.9	2E-11

Query= MT-2_S8_L001_R1_001_(paired)_trimmed_(paired)_contig_168
Length=206

Sequences producing significant alignments	IS Family	Group	Origin	Score (bits)	E. value
ISAjo2	ISNCY	IS1202	Acinetobacter johnsonii	258	3E-69
ISAba32	ISNCY	IS1202	Acinetobacter baumannii	50.1	0,000001

Query= MT-2_S8_L001_R1_001_(paired)_trimmed_(paired)_contig_169
Length=206

Sequences producing significant alignments	IS Family	Group	Origin	Score (bits)	E. value
ISAjo2	ISNCY	IS1202	Acinetobacter johnsonii	254	5E-68

Query= MT-2_S8_L001_R1_001_(paired)_trimmed_(paired)_contig_171
Length=205

Sequences producing significant alignments	IS Family	Group	Origin	Score (bits)	E. value
ISAjo2	ISNCY	IS1202	Acinetobacter johnsonii	192	1E-49
ISAlw22	ISNCY	IS1202	Acinetobacter lwoffii	61.9	3E-10
ISAba32	ISNCY	IS1202	Acinetobacter baumannii	61.9	3E-10

Query= MT-2_S8_L001_R1_001_(paired)_trimmed_(paired)_contig_172
Length=204

Sequences producing significant alignments	IS Family	Group	Origin	Score (bits)	E. value
ISAjo2	ISNCY	IS1202	Acinetobacter johnsonii	254	5E-68
ISAba32	ISNCY	IS1202	Acinetobacter baumannii	85.7	2E-17

Query= MT-2_S8_L001_R1_001_(paired)_trimmed_(paired)_contig_173
Length=204

Sequences producing significant alignments	IS Family	Group	Origin	Score (bits)	E. value
ISAjo2	ISNCY	IS1202	Acinetobacter johnsonii	270	8E-73
ISAba32	ISNCY	IS1202	Acinetobacter baumannii	77.8	6E-15

Query= MT-2_S8_L001_R1_001_(paired)_trimmed_(paired)_contig_174
Length=204

Sequences producing significant alignments	IS Family	Group	Origin	Score (bits)	E. value
ISAjo2	ISNCY	IS1202	Acinetobacter johnsonii	246	1E-65
ISAba32	ISNCY	IS1202	Acinetobacter baumannii	81.8	4E-16

Query= MT-2_S8_L001_R1_001_(paired)_trimmed_(paired)_contig_175
Length=204

Sequences producing significant alignments	IS Family	Group	Origin	Score (bits)	E. value
ISAjo2	ISNCY	IS1202	Acinetobacter johnsonii	161	5E-40
ISAlw22	ISNCY	IS1202	Acinetobacter lwoffii	52.0	0,0000003
ISAba32	ISNCY	IS1202	Acinetobacter baumannii	52.0	0,0000003

Query= MT-2_S8_L001_R1_001_(paired)_trimmed_(paired)_contig_177
Length=204

Sequences producing significant alignments	IS Family	Group	Origin	Score (bits)	E. value
ISAjo2	ISNCY	IS1202	Acinetobacter johnsonii	262	2E-70
ISAba32	ISNCY	IS1202	Acinetobacter baumannii	77.8	6E-15

Query= MT-2_S8_L001_R1_001_(paired)_trimmed_(paired)_contig_179
Length=203

Sequences producing significant alignments	IS Family	Group	Origin	Score (bits)	E. value

IS*Ajo2*	IS*NCY*	IS*1202*	*Acinetobacter johnsonii*	394	2E-110
IS*Alw22*	IS*NCY*	IS*1202*	*Acinetobacter lwoffii*	111	4E-25
IS*Aba32*	IS*NCY*	IS*1202*	*Acinetobacter baumannii*	111	4E-25
IS*Lad2*	IS*NCY*	IS*1202*	*Leclercia adecarboxylata*	56.0	0,00000002
IS*Rel10*	IS*NCY*	IS*1202*	*Rhizobium etli*	46.1	0,00002

Query= MT-2_S8_L001_R1_001_(paired)_trimmed_(paired)_contig_180
Length=203

Sequences producing significant alignments	*IS Family*	*Group*	*Origin*	Score (bits)	E. value
IS*Aba1*	IS*4*	IS*10*	*Acinetobacter baumannii*	379	1E-105
IS*Aba33*	IS*4*	IS*10*	*Acinetobacter baumannii*	192	1E-49

Query= MT-2_S8_L001_R1_001_(paired)_trimmed_(paired)_contig_181
Length=203

Sequences producing significant alignments	*IS Family*	*Group*	*Origin*	Score (bits)	E. value
IS*Ajo2*	IS*NCY*	IS*1202*	*Acinetobacter johnsonii*	313	6E-86

Query= MT-2_S8_L001_R1_001_(paired)_trimmed_(paired)_contig_182
Length=202

Sequences producing significant alignments	*IS Family*	*Group*	*Origin*	Score (bits)	E. value
IS*Ppu12*	IS*L3*		*Pseudomonas putida*	283	5E-77

Query= MT-2_S8_L001_R1_001_(paired)_trimmed_(paired)_contig_185
Length=200

Sequences producing significant alignments	*IS Family*	*Group*	*Origin*	Score (bits)	E. value
IS*Ajo2*	IS*NCY*	IS*1202*	*Acinetobacter johnsonii*	262	2E-70
IS*Aba32*	IS*NCY*	IS*1202*	*Acinetobacter baumannii*	52.0	0,0000003
IS*Alw22*	IS*NCY*	IS*1202*	*Acinetobacter lwoffii*	44.1	0,00008
